# Survival motor neuron protein-independent amelioration of spinal muscular atrophy by pharmacological inhibition of c-Jun-NH_2_ terminal kinase

**DOI:** 10.1093/braincomms/fcag111

**Published:** 2026-03-26

**Authors:** Annapoorna Kannan, Kanchan Bhatia, Xiaoting Jiang, Olivia Lesnik, Sabahat B Asad, Kailyn Fiocca, Saif Ahmad, Yangbo Feng, Dana Branzei, Laxman Gangwani

**Affiliations:** Center for Human Genetics, University of Oxford, Oxford OX3 7BN, UK; School of Mathematical and Natural Sciences, NCIAS, Arizona State University, Glendale, AZ 85306, USA; Department of Immunology, Houston Methodist Research Institute, Houston, TX 77030, USA; Bond Life Sciences Center, University of Missouri, Columbia, MO 65211, USA; Department of Pathobiology and Integrative Biomedical Sciences, University of Missouri, Columbia, MO 65211, USA; Bond Life Sciences Center, University of Missouri, Columbia, MO 65211, USA; Department of Pathobiology and Integrative Biomedical Sciences, University of Missouri, Columbia, MO 65211, USA; Hunt School of Dental Medicine, Texas Tech University Health Sciences Center El Paso, El Paso, TX 79905, USA; Department of Neurosurgery, Barrow Neurological Institute, St.Joseph’s Hospital and Medical Center, Dignity Health, Phoenix, AZ 85013, USA; Department of Molecular and Cellular Pharmacology, Sylvester Comprehensive Cancer Center, Miller School of Medicine, University of Miami, Miami, FL 33136, USA; The AIRC Institute of Molecular Oncology Foundation, IFOM ETS, Milan 20139, Italy; University of Duisburg-Essen, Research Center One Health Ruhr, University Alliance Ruhr, , Essen 45141, Germany; Bond Life Sciences Center, University of Missouri, Columbia, MO 65211, USA; Department of Pathobiology and Integrative Biomedical Sciences, University of Missouri, Columbia, MO 65211, USA

**Keywords:** SMA, JNK, SMN-independent, ZPR1, neurodegeneration

## Abstract

Spinal muscular atrophy (SMA) is an autosomal recessive neurodegenerative disorder caused by mutation of the *survival motor neuron 1* (*SMN1*) gene. SMA is characterized by degeneration of the spinal cord motor neurons caused by chronic low levels of survival motor neuron (SMN) protein. Prevention or slowing of neurodegeneration has been shown to ameliorate SMA disease severity. Significant progress has been made to develop SMN-dependent treatments that increase SMN levels. However, there is an unmet need to develop alternative therapeutic methods that are SMN-independent. The c-Jun-NH_2_ terminal kinase (JNK) signalling pathway mediates motor neuron degeneration in SMA. Genetic inactivation of the neuron-specific isoform, JNK3, ameliorates the disease phenotype in SMA mice without affecting SMN protein levels, indicating that JNK3 may represent a promising SMN-independent target for pharmacological intervention. We report that pharmacological inhibition of JNK using novel drug compounds based on three distinct chemical scaffolds, Anthrapyrazolone, Pyrimidinyl, and Pyridopyrimidine, prevents degeneration of SMN-deficient *in vitro* cultured primary cerebellum neurons and the spinal cord motor neurons derived from SMA mice. Furthermore, *in vivo* treatment with JNK inhibitors leads to a systemic improvement in the disease phenotype, promoting enhanced overall growth, including increased body weight and extended postnatal growth, alongside improved gross motor functions such as righting reflexes and the ability to walk until the later stages of survival. Notably, it also results in a significant and sustained increase in the lifespan of both male and female SMA mice. The sex-based analysis reveals male- and female-specific improvements that depend on the type and efficacy of inhibitors targeting distinct JNK isoforms. Importantly, treatment with JNK inhibitors did not affect SMN levels in the spinal cord or skeletal muscle, indicating that the observed rescue of the SMA phenotype occurs independently of SMN restoration. Collectively, these findings suggest that pharmacological inhibition of JNK may serve as a therapeutic strategy to prevent neurodegeneration, either in combination with SMN-enhancing approaches for treating severe forms of SMA, or as a stand-alone, SMN-independent intervention for moderate and mild SMA cases.

## Teaser

Kannan *et al*. report that the pharmacological inhibition of the c-Jun-NH_2_ terminal kinase pathway using three drug compounds with distinct chemical scaffolds ameliorates spinal muscular atrophy (SMA) disease in mice without altering survival motor neuron (SMN) protein levels. This study provides a pharmacological proof-of-concept for developing an SMN-independent therapeutic method for the treatment of SMA.

## Graphical Abstract

**Graphical Abstract:**
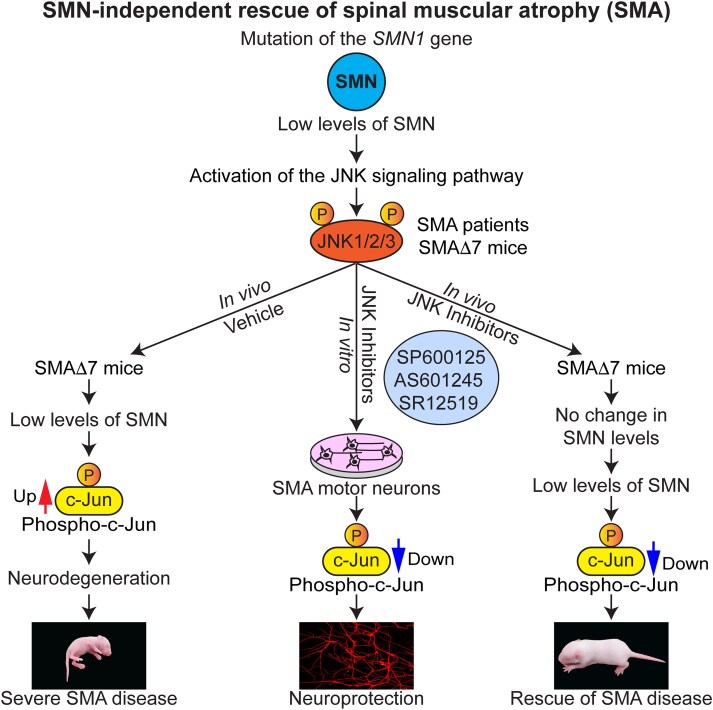

## Introduction

Spinal muscular atrophy (SMA) is an autosomal recessive neuromuscular disorder caused by a homozygous mutation in the telomeric copy of the *survival motor neuron 1* (*SMN1*) gene.^1^ SMA is also a developmental disorder with onset during infant and early childhood stages and stable course, characterized by atrophy of skeletal muscle due to the degeneration of the spinal cord motor neurons caused by the low levels of SMN protein.^2^ Humans harbour two copies of the *SMN* gene, *SMN1* and *SMN2*. *SMN2*, the centromeric copy of the gene, undergoes alternative splicing due to a single point mutation in coding exon 7, resulting in predominant production (90%) of a truncated isoform lacking exon 7 (SMNΔ7).^3^ The low levels (10%) of SMN protein produced by *SMN2* are sufficient for development during embryogenesis, but result in degeneration of the spinal cord motor neurons during postnatal development leading to muscle atrophy, symmetric limb paralysis, respiratory failure, and death in SMA patients.^4^

SMA disease phenotype is modulated through both SMN-dependent and SMN-independent mechanisms, indicating that therapeutic strategies targeting either pathway could be effective for treating SMA. Impressive progress has been made towards developing SMN-dependent methods for the treatment of SMA. Currently, there are three SMN-dependent methods of treatment for SMA that are approved by the Food and Drug Administration (FDA) in the USA; (i) Nusinersen or Spinraza, a drug that utilizes an antisense oligonucleotide (ASO)-based method to correct *SMN2* mRNA splicing by including exon 7, (ii) AVXS-101 or Zolegensma (omasemnogene abeparvovec), a gene therapy-based method using non-replicating self-complementary adeno-associated viral vector carrying *SMN1* gene (scAAV9-*SMN1*) to restore SMN protein levels, and (iii) Evrysdi or Risdiplam, a small, orally deliverable drug compound, which corrects *SMN2* splicing and increases inclusion of coding exon 7 to produce full-length normal SMN protein.^5,6^ However, there are many challenges and gaps in the knowledge of important aspects of current SMA treatments, including side effects and post-treatment complications that are emerging with the aging of patients. Furthermore, considerations include time points of treatment such as pre- or post-symptomatic windows, responders and non-responders, systemic rescue of organs, and sufficient increase in SMN levels in a genetically heterogenous population with a broad spectrum of SMA disease severity.^6-10^ Recently approved SMA treatments have noticeably improved the health, lifestyle and lifespan of SMA patients. However, current treatments are subject to post-treatment follow up studies aimed to identify milestones of achievement and complications and evaluate the possibility of life-long rescue from the pathogenesis of SMA disease.^11-13^ Nonetheless, available SMA treatments have provided an insight into developing advanced strategies to achieve a full and systemic rescue of SMA disease. Pre-clinical studies with animal models and clinical trials of human patients suggest that full systemic rescue or cure of SMA disease may require the development of additional SMN-dependent, and importantly SMN-independent methods that could be used in combination with SMN-dependent methods.^14^ Currently identified SMN-dependent modifier genes include *SMN2* and *zinc finger protein 1* (*ZPR1*).^15-19^ Interestingly, SMN-independent modifiers of SMA have also been identified such as *plastin 3* (*PLS3*),^20,21^  *neurocalcin delta* (*NCALD*),^22^  *stathmin-*1 (*STMN1*),^23^  *Z*  *+*  *Agrin*,^24^ and *senataxin* (*SETX*).^25^ We identified the *c-Jun NH_2_-terminal kinase 3* (*JNK3*) gene as a potential SMN-independent target for the development of a therapeutic strategy. Genetic inhibition of neuron-specific isoform *Jnk3* resulted in systemic amelioration of disease phenotype and improved overall growth, gross motor function, neuromuscular junctions (NMJs) innervation and increased skeletal muscle fibre size and lifespan without altering SMN protein levels in SMA mice.^26^

In this study, we investigated the therapeutic potential of pharmacological inhibition of the JNK signalling pathway in preventing neurodegeneration and in ameliorating neuromuscular disease severity using the SMAΔ7 mouse model.^27^ Activation of the JNK signalling pathway was identified as a mediator of degeneration of SMN-deficient neurons.^26,28^ The ZPR1 binds to SMN and is required for the nuclear accumulation of SMN, has been shown to be downregulated in SMA patients. ZPR1 deficiency contributes to activation of the JNK pathway and neurodegeneration in SMA.^16,28,29^ An earlier study showed that JNK inhibition using pan-JNK peptide inhibitor D-JNKI1 with broad specificity for all JNK isoforms resulted in the amelioration of disease severity with only a very small but statistically significant increase in the survival of SMA mice.^30^ This finding supports the conceptual advance made by Genabai *et al*.^26^ and calls for further investigation of pharmacological JNK inhibition for the treatment of SMA. Recent findings show that activation of the JNK pathway may be connected with R-loop-mediated DNA damage and neurodegeneration in SMA.^31^ Targeting of the JNK pathway has demonstrated therapeutic potential for treating diverse diseases, including neurodegenerative disorders such as amyotrophic lateral sclerosis (ALS), Huntington’s disease, Parkinson’s disease, and Alzheimer’s disease.^32-43^ Initial clinical trials had limited success due to off-target effects and cellular toxicity of JNK inhibitor drug compounds. Despite these challenges the therapeutic potential of targeting JNK kinases remains promising.^44-46^ These problems identified a need for developing better classes of chemical compounds with minimal off-target effects and toxicity. Recent advances in chemistry have allowed for the synthesis of novel classes of JNK inhibitor compounds with specificity for JNK inhibition and minimum off-target effects. However, one of the critical aspects of pharmacological research investigation requires *in vitro* testing of drug compounds in relevant primary cell types before proceeding for *in vivo* testing. In general, culturing of primary spinal cord neurons for routine experiments from normal and disease mice has been very challenging. To overcome this limitation, we have developed method for culturing primary spinal cord neurons from postnatal normal and symptomatic SMA mice.^16,25,47^ We examined the effects of JNK inhibitors with different chemical scaffolds on JNK inhibition and neuroprotection *in vitro* using cultured primary spinal cord neurons derived from SMA mice. We investigated the *in vivo* effects of our top-three drug compounds with distinct chemical scaffolds, anthrapyrazolone (SP600125),^48^ pyrimidinyl (AS601245),^49^ and pyridopyrimidine (SR12519),^50^ with broad or targeted specificity for three kinase isoforms, JNK1, JNK2, and JNK3, using a severe SMA mouse model. We compared improvements in the sex-based disease phenotypes of SMA mice treated with different drug compounds. The findings of this study offer important insights into the feasibility of developing an SMN-independent pharmacological approach for treating both severe and mild forms of SMA.

## Materials and methods

### Mice

The wild-type mice on FVB/N genetic background and severe SMA mouse model with genotype (*Smn*^−/+^; *SMN2^+/+^*; *SMNΔ7^+/+^*) (mouse line #4299)^27^ on FVB/N genetic background were purchased from the Jackson Laboratory and maintained in the our laboratory. All research animals (mice) were housed in a facility accredited by the Association for Assessment and Accreditation of Laboratory Animal Care (AAALAC). Experiments with animals, including behavioural analysis and use of mouse tissues for biochemical methods were approved by the Institutional Animal Care and Use Committee and by the Institutional Biosafety Committee of the Texas Tech University Health Sciences Center El Paso (TTUHSC EP) and the University of Missouri, Columbia. Animal studies were carried out at TTUHSCEP. Animals were treated humanely, and euthanasia was performed using methods approved by the American Veterinary Medical Association. SMA carrier mice (*Smn*^−/+^; *SMN2^+/+^*; *SMNΔ7^+/+^*) were bred to generate mice with SMA (*Smn*^−/−^; *SMN2^+/+^*; *SMNΔ7^+/+^*). SMA mice littermates at postnatal day 2 (PND2) were injected intraperitoneally (IP) with a single dose of vehicle dimethyl sulfoxide (DMSO) or JNK inhibitor, SP600125 (20 mg/kg) or AS6011245 (20 mg/kg) or SR12519 (10 mg/kg) every other day. The international union of pure and applied chemistry chemical names of the JNK inhibitors are, SP600125—Anthra[1,9-cd]pyrazol-6(2H)-one (1,9-pyrazoloanthrone), AS601245—1,3-benzothiazol-2-yl(2-((2-(3-pyridinyl)ethyl)amino)-4-pyrimidinyl) acetonitrile, and SR12519—1-isopropyl-3-((1r,4r)-4-((8-isopropyl-6-methyl-7-oxo-7,8-dihydropyrido[2,3-d]pyrimidin-2-yl)amino)cyclohexyl)urea. All pups that died in different groups at PND1 and PND2 were excluded from the analysis. *In vivo* testing of three inhibitors was performed by three different individuals at different times with no overlap. Littermates for injections of vehicle or drug compounds were selected randomly by one person and data was collect by another person. Analysis of phenotypes, including whole body weight, time-to-right (TTR), hindlimb suspension test (HLST) and survival were blinded, and performed with littermates. The genotyping of littermates was performed after collection of all behavioural data by PCR using tail DNA that contributed to the blinding of experiments.

### Behavioural analysis

#### Growth analysis

Mice weight of littermates was measured and recorded daily using a digital balance in the laminar flow hood in the animal facility.

#### Motor function analysis

Righting reflexes of littermates were measured as a TTR with a test time limit of 30 s.^16,26^ Average time of three recoding/per day/mice was used for quantitative and statistical analysis.

#### Muscle strength test

To measure the muscle strength, we used hindlimb suspension test (HLST) to evaluate improvement in the proximal hind-limb muscle strength.^16,26,51,52^ Mice littermates with age range of 5–9 days were hanged on both hind legs on the edge of a 50 ml plastic conical tube fitted with soft cotton pad at the bottom and time was recorded until their fall from the edge of tube with a cut-off time limit of 30 s. Average time of three recoding/per day/mice was used for quantitative and statistical analysis.

### Primary neuron culture

#### Cerebellar granule neurons

Primary cerebellar granule neurons (CGN) were isolated from the cerebellum of 7-day-old wild-type FVB/N mice and cultured *in vitro* for 8 days in a 96-well plate or 8-well chambers microscope slides (Corning), coated with poly-D-lysine (PDL)/laminin in neurobasal medium supplemented with B-27, glutamine, 700 mM Glucose, Glutamine, 2.5 mM KCl and penicillin/streptomycin as described previously.^28,53^ Neurons ∼150 000/well in 8-well chambers were plated and transfected with 100 nM siRNA specific for mouse *Smn* (M-044280-00) or Scramble II (Control, 5′-GCGCGCTTTGTAGGATTCG-3′) oligos from Dharmacon using Lipofectamine®2000.^26,54,55^

#### Cell viability assay

Purified CGN from wild-type mice were counted using Bio-Rad cell counter and 50 000 cells/well were plated onto PDL coated 96-well plates. Neurons were cultured for 6 days and treated with pan-JNK or JNK3-specific inhibitors: SP600125, AS601245, AS602801, SR12272, SR11935, SR12055, SR12735, SR12519, SR12370, SR12275, JNK-In-8, JNK-In-7, F15, F13M, F14A, F23C, F3A, F4A, F9A, and SR3562 for 48 h with final concentrations (μM) of 0.25, 0.50, 1.0, 2.0, 4.0, 8.0.^50,56-60^ All inhibitor compounds were dissolved in DMSO and stocks were prepared in 50% DMSO (v/v). Cell viability was assessed using Trevigen’s TACS® MTT Cell Proliferation Assay as per manufacturer’s instructions and absorbance was read using a microplate reader (BioTek Instruments, Inc.) at 570 and 690 nm. Each sample was assayed in triplicate in three independent experiments and relative cell viability was calculated as % of control.

#### Testing JNK inhibitor compounds for their efficacy in suppressing JNK activity in cultured SMN-deficient neurons

The effect of JNK inhibitors was examined by treating transfected (siRNA) neurons with DMSO or inhibitors. Neurons transfected with SMN specific siRNA (siSMN) were treated with DMSO or JNK inhibitors (1.0 μM) after 24 h (post-transfection). Neurons were incubated with inhibitors for additional 48 h, washed with phosphate buffer saline (PBS) and fixed with 4% paraformaldehyde (PFA) for examination by immunofluorescence (IF) analysis.

#### Spinal cord motor neurons

The spinal cords were dissected from 7-day-old normal and SMA mice littermates and cut into small pieces. The explants were cultured and differentiated *in vitro* for 12–14 days in serum-free neurobasal medium supplemented with B-27 (1×), 25 mM glucose, 25 mM KCl, 200 mM glutamine and penicillin/streptomycin using 8-well chamber microscope slides coated with laminin and PDL (Corning).^16,25,26^ The morphology and identity of the spinal cord motor neurons were established by staining with specific markers, including neuron-specific β-tubulin-III, choline acetyltransferase and homeobox Hlxb9 (Hb9).^25,26^ The spinal cord neurons were treated with JNK inhibitors (1.0 μM) for 6 days, with the culture medium replaced and supplemented with fresh inhibitor every 48 h. After treatment with inhibitors, neurons were washed with PBS and fixed with 4% PFA for IF analysis.^16^

#### Immunofluorescence analysis

CGN or spinal cord motor neurons cultured in 8-well chamber microscope slides were washed with 1× PBS and fixed with 4% PFA for examination by IF analysis. PFA fixed primary neurons were washed with PBS, permeabilized with 0.1% Triton-X100 for 5 min, washed 3 × 5 min with PBS with 0.2% Tween-20 (PBS-T), blocked with 3% BSA in PBS-T for 30 min and double labelled using sequential incubation (1 h each) with primary antibodies phospho-c-Jun (p-c-Jun) (Ser63) #9261, (Cell Signaling) and followed by secondary antibody Alexa 488-conjugated IgG (green), washed 3 × 5 min with PBS-T and incubated with second primary antibody, anti-β-tubulin class-III neuron-specific antibody anti-β-tubulin class-III neuron-specific, (TUJ1, R&D systems), washed 3 × 5 min with PBS-T followed by incubation with Alexa 594-conjugated IgG (red) anti-mouse or anti-rabbit IgG secondary antibody.^25^ After staining, the plastic frames of the 8-well culture chambers were removed, and mounting medium containing DAPI (Vectashield) was added to the wells on the glass slide, followed by placement of glass coverslips.^25,61^ IF of stained cells was examined using confocal microscope Leica SP5 equipped with acousto-optical beam splitter and UV (405 nm) laser.^26^

#### Immunoblot analysis

Protein extracts for immunoblot (IB) analysis were prepared from mouse spinal cord, and muscle tissues collected from 10-day-old (PND11) normal (untreated) and SMA mice littermates treated with DMSO and drug compounds. Tissue lysates were prepared using 1× Triton-X100 lysis buffer.^62^ Proteins of interest, SMN, p-c-Jun, c-Jun, and tubulin were detected by automated western blot system, Wes System (ProteinSimple), which utilizes capillary based electrophoretic separation and detection of proteins using antibodies, as previously described.^16,25,47^ The following primary phospho and non-phospho antibodies were used for IB analysis, SMN (610647, BD Biosciences), p-c-Jun (Ser63) #9261 and c-Jun (60A8) #9165 (Cell Signaling) and α-tubulin (T8203, Sigma). Signal intensity (area) of the protein was normalized to the peak area of loading control α-tubulin. Data analysis and Quantitation of protein levels was performed by measuring signal intensity (area) of each protein and was normalized to the peak area of loading control α-tubulin using Compass Software (ProteinSimple).^25^ The relative amounts of proteins normalized to tubulin [mean ± standard error mean (SEM)] are presented.

#### Statistical analysis

The quantitative analysis of continuously distributed data [mean ± SEM and mean ± standard deviation (SD)] from different experiments is presented as growth curves, survival curves, box-and-whisker, scattered plots, and violin plots with quantitative elements, including median with interquartile interval and minimum and maximum ranges. The SEM and SD are used to quantify variability. The sample size (*n* = 15/group) for Kaplan–Meier analysis was estimated with statistical power equal or >80% with significance set at 0.01 level using PASS 12 software.^63,64^ For sex-based analysis, the sample size (9.99 = 10)/sex was estimated using software R version 4.2, and ANOVA power estimation (80%) using three groups (normal, SMA and SMA + JNK inhibitor), Cohen’s *d* = 0.6 (standardized mean difference) for a moderate-strong effect, and a significance level of 0.05 with the assumption of keeping male and female mice partitioned. Statistical analysis was performed using appropriate statistical methods, including one-way ANOVA, Student’s *t*-test (unpaired, two-tailed) and Log-rank (Mantel–Cox) test with GraphPad Prism. A *P-*value equal or <0.05 was considered statistically significant. Data collection for *in vivo* phenotypic analysis of mice was performed with a minimum of *n* = 3 mice/group. In all *in vitro* experiments with cells, ‘*n*’ represents the number of times an experiment was performed and in experiments with tissues ‘*n*’ represents the number of animals used per group. A minimum of *n* = 3 was used in all the experiments, unless otherwise specified in specific experiments.

## Results

### Prevention of degeneration of SMN-deficient neurons by JNK inhibition

We have previously shown that the JNK signalling pathway mediates neurodegeneration in SMA.^26^ We also identified a neuron-specific isoform of JNK, JNK3, which mediates spinal cord motor neuron degeneration in SMA using spinal cord tissues from SMA mice and patients.^26^ Furthermore, we demonstrated that the genetic inactivation of JNK3, achieved by deleting the *Jnk3* gene in SMA mice reduced neurodegeneration, improved overall growth and motor function, reduced the severity of disease, and increased the lifespan of severe SMA mice.^26^ Together, these findings provided a rationale for examining the effects of pharmacological inhibition of JNK on the prevention of neurodegeneration and the amelioration of SMA disease severity.

#### Identification of promising JNK inhibitors drug candidates

**Figure 1:**
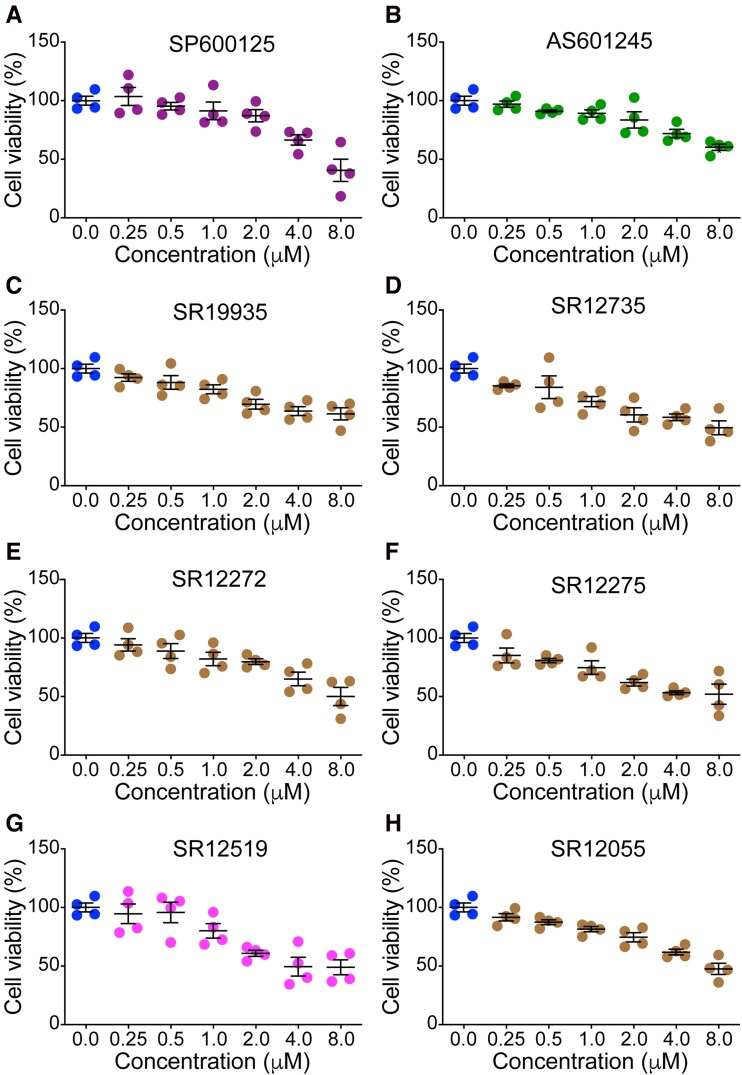
**The effect of JNK inhibitors on the cell viability of cultured primary neurons.** Cerebellums were isolated from 7-day-old mice and CGN were purified and cultured *in vitro* for 8 days. Cell viability was determined using MTT assay after treatment of neurons with drug compounds for 24 h. Twenty drug compounds were screened for cell viability and toxicity. Relative cell viability (%) is presented as scatter plots with individual points for the different concentrations of JNK inhibitors used for treatment. Each data point represents an average of three technical replicates. The cell viability assay data for eight promising compounds with low toxicity and high neuron survival (>70–90%) upon treatment with inhibitors at 1.0 μM is, SP600125 (91.32 ± 7.48), AS601245 (89.14 ± 3.05), SR11935 (82.37 ± 3.79), SR12735 (71.90 ± 4.30), SR12272 (82.10 ± 5.73), SR12275 (74.75 ± 5.68), SR12519 (80.02 ± 6.17) and SR12055 (81.41 ± 2.62). The results are presented as (mean ± SEM, *n* = 4). The cell viability data at other concentrations of drug compounds are presented in Supplementary Table 1. Data from the testing of 12 other drug compounds is presented in Supplementary Fig. 1 and Supplementary Table 2.

To identify the most promising JNK inhibitor compounds with low toxicity, potent pan-JNK or JNK3-selective activity, and assess their ability to prevent degeneration of SMN-deficient neurons, we conducted a screening of 20 drug compounds with different chemical scaffolds (Supplementary Fig. 1). The screen included SP600125, a well-studied JNK inhibitor previously used in clinical trials of cancer, as a positive control in our assays for comparison.^48,65-67^ First, we tested the toxicity of increasing concentrations of inhibitors on cultured primary CGN, due to a large number of neurons required for testing. Neurons were treated with DMSO (control) and compounds for 48 h with final concentrations (μM) of 0.25, 0.50, 1.0, 2.0, 4.0, 8.0. Analysis of cell viability assay data identified eight promising compounds with low toxicity and high neuron survival (>70–90%) when 1.0 μM concentration of inhibitors, SP600125 (91.32 ± 7.48), AS601245 (89.14 ± 3.05), SR11935 (82.37 ± 7.39), SR12735 (71.90 ± 4.30), SR12272 (82.10 ± 5.73), SR12275 (74.75 ± 5.68), SR12519 (80.02 ± 6.17), SR12055 (81.41 ± 2.62), was used (Fig. 1A–H, Supplementary Fig. 1, Supplementary Table 1).

#### Effect of JNK inhibitors on protecting SMN-deficient neurons

**Figure 2:**
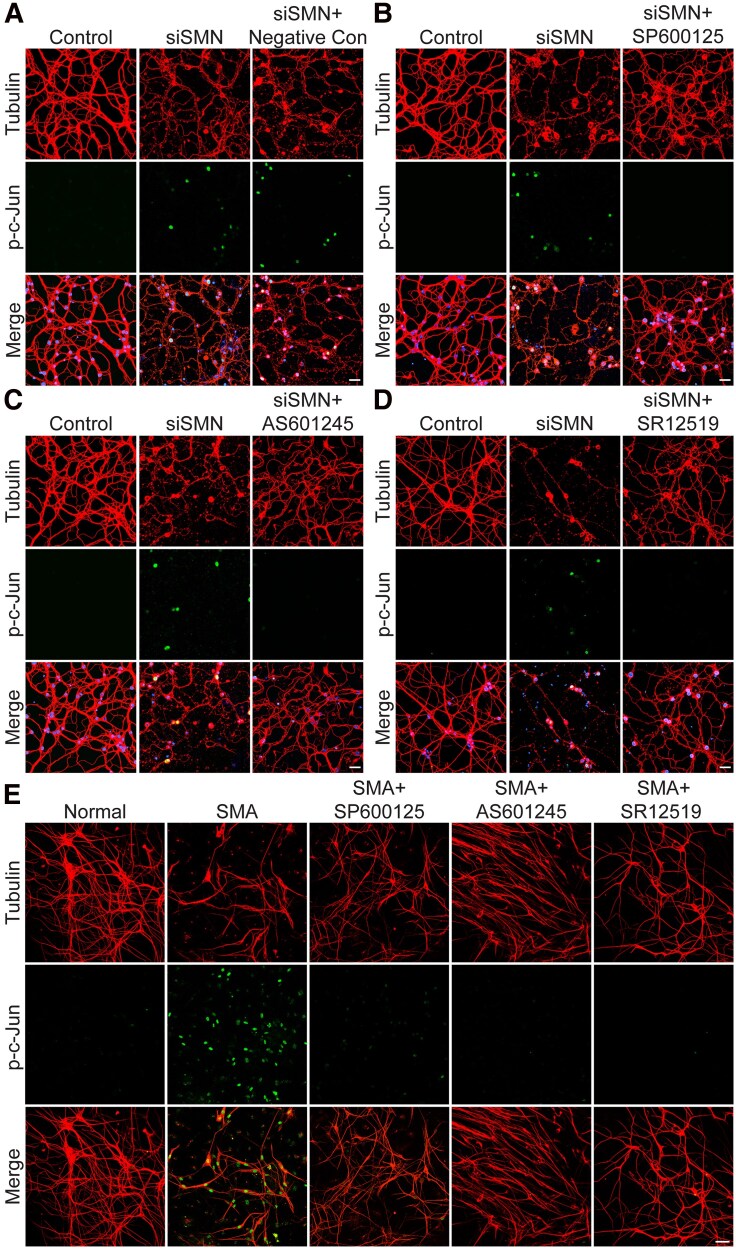
**The effect of JNK inhibitors on preventing the degeneration of SMN-deficient cultured primary neurons and spinal cord motor neurons derived from SMA mice.** Cultured primary neurons (CGN) were transfected with siRNA (siSMN) 100 nM and incubated for 24 h before treatment with inhibitors (1.0 μM). Neurons were fixed after 24 h treatment of inhibitor with 4% PFA and stained with antibodies to neuron-specific β-tubulin class III (red) and p-c-Jun (green) and IF examined by confocal laser scanning microscopy. Degeneration of neurons caused by SMN knockdown (siSMN) is shown by axonal degeneration compared with control neurons stained with tubulin (red). Nuclei were stained with DAPI (blue). (**A**) Treatment with JNKII, a negative control for JNK inhibition, did not cause decrease in p-c-Jun (green) levels (siSMN + JNKII) and did not reduce the degeneration of neurons compared with SMN-deficient neurons (siSMN). Treatments with (**B**) SP600125, (**C**) AS601245, and (**D**) SR12519 show JNK inhibition causes a decrease in p-c-Jun (green) levels (siSMN + SP600125) compared with SMN-deficient neurons (siSMN) and results in a reduction in the degeneration of neurons (red) showing protection of SMN-deficient neurons by pharmacological JNK inhibition. (**E**) Pharmacological inhibition of JNK prevents degeneration and improves the growth of spinal cord motor neurons derived from SMA mice. Primary spinal cord neurons were cultured from 7-day-old normal and SMA mice littermates. Neurons were differentiated *in vitro* for 14 days, treated with JNK inhibitors (1.0 μM) for 6 days with a change of medium supplemented with fresh inhibitors every 48 h and fixed with 4% PFA. Fixed neurons were stained with antibodies against neuron-specific β-tubulin-III (red), and p-c-Jun (green), and IF was examined by confocal microscopy. Neurons from normal mice show healthy motor neurons with very low staining for p-c-Jun (green). Neurons from SMA mice (SMA + DMSO) show signs of degeneration (arrows) and high staining for p-c-Jun (green) compared with neurons from normal mice. Treatments with JNK inhibitors, SP600125, AS601245, and SR12519 reduces JNK and causes a decrease in p-c-Jun (green) levels (SMA + SP600125 or AS601245 or SR12519) compared with SMA neurons treated with DMSO (SMA + DMSO), leading to protection and improved growth of SMA spinal cord motor neurons by pharmacological JNK inhibition. Scale bar is 50 μm.

To identify potent JNK inhibitor compounds that can prevent degeneration of SMN-deficient neurons, we examined the effects of eight, short-listed compounds on the protection of primary CGN neurons with knockdown of SMN using RNAi.^26^ We have previously shown that the knockdown of the *Smn* gene expression in mouse CGN results in a reduction of SMN levels to ∼30%, which causes JNK activation and the degeneration of neurons.^26^ The specificity of knockdown by siRNA-Smn was established by performing control experiments using transfections with siRNA-Scramble with IB and IF analysis.^26^ Previous studies have shown defects in the cerebellum, including neurodegeneration in SMA patients and mice suggesting CGN as relevant cell type for SMA studies.^68-70^ We employed this neuron-based SMA model to examine the effects of nine compounds, eight inhibitors (SP600125, AS601245, SR12272, SR11935, SR12055, SR12735, SR12519, SR12275) and JNKII, a negative control drug compound for JNK inhibition.^71^ Neurons were treated with inhibitors (1.0 μM) after 24 h of transfection with siRNA and incubated for 48 h with inhibitors. Neurons were fixed and stained with antibodies against p-c-Jun and neuron-specific tubulin for IF analysis. Knockdown of SMN results in the activation of JNK as shown by the p-c-Jun, a downstream target of JNK (p-c-Jun, green) and the degeneration of SMN-deficient neurons (tubulin, red) (siSMN) (Fig. 2A). Treatment with negative control for JNK inhibition did not inhibit JNK and was unable to prevent neuron degeneration suggesting that JNK inhibition may be required for neuroprotection (Fig. 2A). Treatment of SMN-deficient neurons (siSMN) with SP600125, AS601245, and SR12519 causes efficient inhibition of JNK, a marked reduction in the p-c-Jun, and prevention of neuron degeneration (Fig. 2B–D). Data on treatment of the additional five JNK inhibitors (SR12272, SR11935, SR12055, SR12735, SR12275) on JNK inhibition and the prevention of neuron degeneration show that these compounds are not potent enough to completely inhibit JNK (p-c-Jun, green) and reduce the degeneration of SMN-deficient neurons (tubulin, red) (Supplementary Fig. 2). A quantitative analysis of tubulin positive (total neurons) and pc-Jun positive neurons show statistically significant reduction in number of p-c-Jun positive neurons (mean ± SEM, *n* = 4)%, which indicates JNK inhibition, in SMN-deficient neurons treated with siSMN (41.97 ± 4.90)% upon treatment with SP600125 (3.59 ± 0.25, *P* = 0.0042), AS601245 (4.05 ± 0.31, *P* = 0.0043), and SR12519 (8.20 ± 0.36, *P* = 0.0061). These results are included in Supplementary Fig. 3. These initial testing experiments with a neuron-based SMA model allowed for the selection of three top drug candidates (SP600125, AS601245, and SR12519) for further testing.

#### Effect of JNK inhibitors on protection of the spinal cord motor neurons derived from SMA mice

SMA disease is characterized by degeneration of the spinal cord motor neurons. Preventing or slowing motor neuron degeneration ameliorates disease severity.^72-74^ To test the potential of top three compounds, SP600125, AS601245, and SR12519, we used cultured primary spinal cord neurons derived from 7-day-old SMA mice. We have developed and established a method for culturing the spinal cord motor neurons from wild-type and diseased mouse models and routinely use it for our *in vitro* studies.^16,25,28,47,51^ Spinal cord neurons were treated with 1.0 μM JNK inhibitors for 6 days, with the medium replaced every 48 h using fresh inhibitor-supplemented medium. Comparison of neurons from normal and SMA mice littermates stained with antibodies against neuron-specific β-tubulin III (tubulin, red) and p-c-Jun (green) show low numbers of neurons in SMA (Fig. 2E). Notably, all spinal cord motor neurons from SMA mice show an accumulation of p-c-Jun in the nucleus (green) compared with motor neurons from normal mice suggesting activation of the JNK signalling pathway in SMA motor neurons (Fig. 2E). This may be caused by low levels of SMN, consistent with data from neurons with SMN knockdown (Fig. 2A–D). Of interest, treatment of SMA motor neuron with all three inhibitors resulted in JNK inhibition associated with a marked reduction in p-c-Jun accumulation in the nucleus compared with untreated SMA neurons (SMA + DMSO), indicating that these JNK inhibitors have the potential to inhibit JNK in motor neurons under SMA disease condition (Fig. 2E). Furthermore, analysis of the growth and quality of SMA neurons (tubulin, red) shows that treatment with all JNK inhibitors resulted in increased growth and better health of cultured neurons compared with SMA neurons treated with DMSO (Fig. 2E). Together, these *in vitro* data suggest that JNK is activated by the low levels of SMN in motor neurons and that JNK inhibition results in prevention of neuron degeneration and improved growth of cultured motor neurons from SMA mice.

### Pharmacological inhibition of JNK rescues SMA phenotype in mice

The systematic *in vitro* analysis of JNK inhibitor drug compounds on neuronal toxicity, neurodegeneration, JNK inhibition and neuroprotection using cultured primary neurons, SMN-deficient neurons and SMA motor neurons allowed for the identification of three top JNK inhibitors for *in vivo* testing using an SMA mouse model. We used the SMAΔ7 model with genotype [*Smn*^-/-^; *SMN2^+/+^*; *SMNΔ7^+/+^*] that show disease phenotype and survival age range corresponding to human severe SMA type-I (subtype Ib and Ic) for examining the effects of pharmacological inhibition of JNK on the amelioration of disease severity. Mice littermates were injected with DMSO and JNK inhibitors at PND2 IP with a single dose of vehicle DMSO (SMA) or JNK inhibitor SP600125 (20 mg/kg) or AS6011245 (20 mg/kg) or SR12519 (10 mg/kg) every other day. Preliminary experiments were performed using normal or wild-type mice (*n* = 3 mice/compound) to test for any adverse effect or toxicity of drug compounds on the health and lifespan of mice. All mice injected with inhibitors until weaning were healthy and lived a normal lifespan. The optimal drug concentrations for this *in vivo* study were determined based on the doses required to protect SMN-deficient neurons and neurons derived from SMA mice *in vitro*, the known potency of the compounds as indicated by their IC_50_ values (50% inhibition of JNK activity), blood-brain-barrier permeability, and data from previous *in vivo* studies using JNK inhibitors in other disease mouse models.^50,58,65,75-81^

### Treatment with pan-JNK inhibitor SP600125 improves overall growth of SMA mice

**Figure 3:**
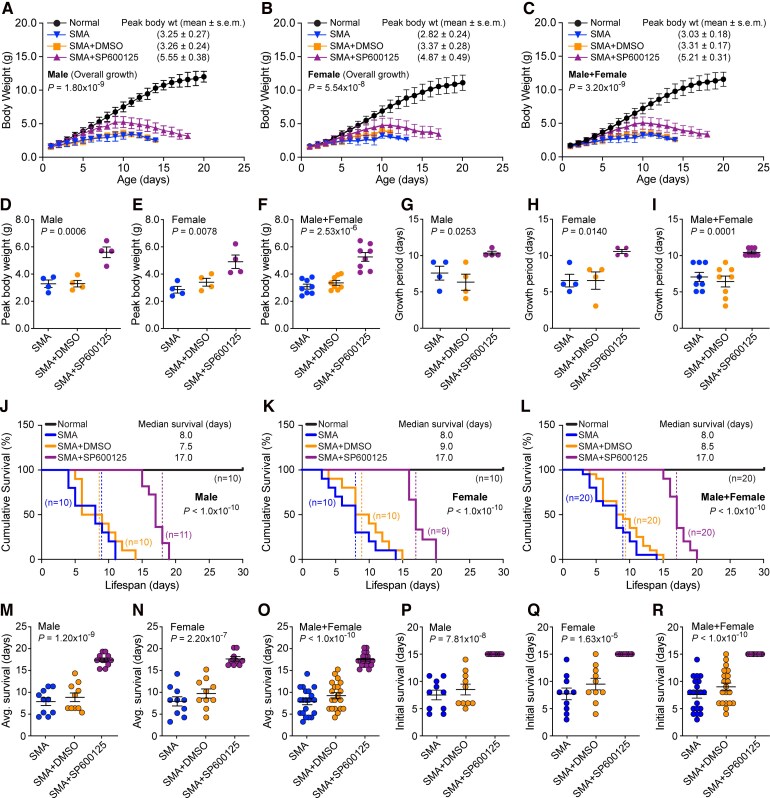
**Pharmacological inhibition of JNK with pan-JNK inhibitor SP600125 improves the overall growth and survival of SMA mice.** SMA (SMAD7) mice littermates starting at PND2 were injected IP with a single dose of vehicle DMSO or JNK inhibitor SP600125 (20 mg/kg) followed by every other day schedule. Amelioration of disease severity, improvement in growth and increase in the lifespan of mice were examined. (**A–C**) Pharmacological inhibition of JNK improves the growth of SMA mice. Overall growth (body weight in grams) curves of normal (black), SMA (blue), SMA + DMSO (orange) and SMA + SP600125 (purple) mice. Mice body weights recorded every day are presented as growth curves with mean ± SD, *n* = minimum 3 mice/group for male, female and combined (male + female) groups. Statistical analysis (ANOVA) shows marked increase in overall growth of mice treated with SP600125, male (*P* = 1.80 × 10^−9^), female (*P* = 5.54 × 10^−8^) and male + female (*P* = 3.20 × 10^−9^). (**D–F**) Average peak body weight (grams) shown as scatter plots with mean ± SEM (*n* = 4/sex/group). Each data point represents a biological replicate. (**D**) Analysis of these data show a statistically significant increase in average peak body weight of SMA + SP600125 males (5.55 ± 0.38) compared with SMA (3.25 ± 0.27) and SMA + DMSO (3.26 ± 0.24) males (*P* = 0.0006, ANOVA), which shows ∼71% increase in the body weight of SMA + SP600125 compared with SMA + DMSO males. (**E**) SMA + SP600125 females (4.87 ± 0.49) (*P* = 0.0078, ANOVA) compared with SMA (2.82 ± 0.24) and SMA + DMSO (3.37 ± 0.28) females show an increase of ∼72% (SMA) and ∼45% (SMA + DMSO) and an average increase of ∼59% in the peak body weight of SMA + SP600125 females. (**F**) Comparison of combined (male + female) peak body weight in SMA + SP600125 (5.21 ± 0.31, *n* = 8) with SMA (3.03 ± 0.18) and SMA + DMSO (3.31 ± 0.17) (*P* = 2.53 × 10^−6^, ANOVA) shows an increase (∼65%) in the average peak body weight of SMA + SP600125 mice. (**G–I**) JNK inhibition improves postnatal growth period (days) (mean ± SEM, *n* = 4/sex/group) of SMA mice. Growth period of (**G**) SMA + SP600125 (10.25 ± 0.25) males compared with SMA + DMSO (6.25 ± 1.10) and SMA (7.50 ± 0.95) male mice shows ∼64% (*P* = 0.0253, ANOVA) increase in postnatal growth period, (**H**) SMA + SP600125 (10.50 ± 0.28) females compared with SMA + DMSO (6.50 ± 1.19) and SMA (6.50 ± 0.86) female mice show ∼62% (*P* = 0.0140, ANOVA) increase in postnatal growth period, (**I**) the comparison of combined (males + females) SMA + SP600125 (10.38 ± 0.18) to SMA + DMSO (6.37 ± 0.75) and SMA (7.0 ± 0.62) males + females shows statistically significant (*P* = 0.0001, ANOVA) increase in postnatal growth period. Growth analysis was blinded with colour coding. Gender and genotypes were confirmed by PCR-based method after euthanasia of pups. (**J–R**) Pharmacological inhibition of JNK improves the survival of SMA mice. (**J–L**) Kaplan–Meier survival curves of normal (black), SMA (blue), SMA + DMSO (orange) and SMA + SP600125 (purple) mice littermates. Dotted lines show median survival. Survival analysis show ∼2-fold increase in median survival of SMA mice treated with the drug SP600125 compared with control SMA mice (Log-rank test, *P* < 1.0 × 10^−10^). (**M–O**) The scatter plots show average survival (days) (mean ± SEM) of SMA mice untreated (SMA), treated with DMSO (SMA + DMSO) and JNK inhibitor SP600125 (SMA + SP600125). (**M**) Sex-based analysis shows average survival of SMA + SP600125 males (17.09 ± 0.41, *n* = 11, *P* = 1.20 × 10^−9^) is higher than SMA + DMSO (8.50 ± 0.99, *n* = 10) and SMA (7.50 ± 0.88, *n* = 10) males. (**N**) Average survival of SMA + SP600125 females (17.44 ± 0.53, *n* = 9, *P* = 2.20 × 10^−7^) is also higher compared with SMA + DMSO (9.50 ± 1.05, *n* = 10) and SMA (7.70 ± 1.06, *n* = 10) females. (**O**) Statistical analysis of average male and female survival shows an increase in average survival (mean ± SEM days, *n* = 20) of SMA + SP600125 (17.25 ± 0.32) (ANOVA, *P* < 1.0 × 10^−10^) compared with SMA + DMSO (9.0 ± 0.71) and SMA (7.60 ± 0.67) mice. (**P–R**) Scatter plots show a marked increase (5-fold) in the initial (minimum) survival of SMA mice treated with JNK inhibitor (SMA + SP600125) compared with SMA and SMA + DMSO mice. All males and females (*n* = 20) treated with SP600125 survived at least 15 days compared with SMA males and females that survived at least 3 days. Survival and growth (body weight) analyses were blinded with colour coding. Sex and genotypes were confirmed by PCR-based method after euthanasia of mice.

To understand the contribution of the JNK group of kinases in the pathogenesis of SMA, we tested three inhibitors with diverse potency towards different JNK isoforms. We started with the testing of the pan-JNK inhibitor SP600125 with IC_50_ of 40 nM (JNK1), 40 nM (JNK2), and 90 nM (JNK3), which has high potency of inhibition for JNK1/2 and a moderate potency for JNK3 and shows the lowest toxicity on cultured primary neurons (Figs 1 and 2). SMA mice treated with pan-JNK inhibitor SP600125 were healthier, and better able to stand and walk compared with control SMA mice treated with vehicle between the ages of 6–15 days. Initial videographic observations suggested that JNK inhibition might be beneficial in improving the overall health and gross motor functions of SMA mice as early as PND6, as shown in video clips of littermates treated with DMSO (black dots) and SP600125 (red dots) at the ages of 6, 10, and 15 days (Supplementary Videos 1–3). To examine any negative effects of treatment with DMSO (vehicle) on the overall health of SMA mice, we compared the overall growth of SMA (untreated) and SMA + DMSO mice littermates. Mice body weights were recorded every day and are presented as growth curves with mean ± SD, *n* = minimum 3 mice/group/day for male, female and male + female groups (Fig. 3A–C). The comparison of overall growth (body weight) of mice among SMA, SMA + DMSO and SMA + SP600125 groups shows statistically significant increase in the combined (male + female) overall growth of SMA mice treated with JNK inhibitor (*P* = 3.20 × 10^−9^) (Fig. 3C). The comparison of average peak body weight (grams) (mean ± SEM, *n* = 4) of males SMA (3.25 ± 0.27) and males SMA + DMSO (3.26 ± 0.24) with *P* = 0.9894, and females SMA (2.82 ± 0.24) and females SMA + DMSO (3.37 ± 0.28) with *P* = 0.1973 did not show any statistically significant difference in the overall growth between SMA and SMA mice treated with DMSO (SMA + DMSO) suggesting that treatment with DMSO did not cause any adverse effect on the health of SMA mice (Fig. 3A and B). Analysis of growth of littermates shows that *in vivo* JNK inhibition with drug compound SP600125 increased average peak body weight (grams) in males SMA + SP600125 (5.55 ± 0.38, *P* = 0.0006) compared with SMA (3.25 ± 0.27) and SMA + DMSO (3.26 ± 0.24) males and in females SMA + SP600125 (4.87 ± 0.49, *P* = 0.0078) compared with SMA (2.82 ± 0.24) and SMA + DMSO (3.37 ± 0.28) females (Fig. 3A and B). Comparison of combined (male + female) peak body weight of SMA + SP600125 (5.21 ± 0.31, *n* = 8) with SMA (3.03 ± 0.18) and SMA + DMSO (3.31 ± 0.17) mice show statistically significant (*P* = 2.53 × 10^−6^) improvement in the growth of SMA mice treated with JNK inhibitor (SMA + SP600125) (Fig. 3C).

The sex-based analysis of overall growth shows significant increase in body weight of male (*P* = 1.80 × 10^−9^) and female (*P* = 5.54 × 10^−8^) SMA mice treated with SP600125 (Fig. 3A and B). Analysis of peak body weight range shows overall growth increase in SMA + SP600125 males is ∼71% (*P* = 0.0006) and in females is ∼59% (*P* = 0.0078) compared with untreated or DMSO treated SMA males and females (Fig. 3D and E). These data show similar improvement in the body weight of male and female mice upon treatment with JNK inhibitor compared with control SMA mice (Fig. 3D–F). JNK inhibition also increased growth period (mean ± SEM, *n* = 4) of male mice from (6.25 ± 1.10) (SMA + DMSO) to (10.25 ± 0.25) (SMA + SP600125) days and female mice from (6.50 ± 1.19) (SMA + DMSO) to (10.50 ± 0.28) (SMA + SP600125) days, showing ∼64% increase in males (*P* = 0.0253) and ∼62% increase in females (*P* = 0.0140) in the postnatal growth periods, respectively (Fig. 3G–I). Together, these data suggest that *in vivo* JNK inhibition improves overall growth as well as postnatal growth period of SMA mice treated with the JNK inhibitor, which contributes to the reduction of disease severity and increases the lifespan of SMA mice.

### JNK inhibition with SP600125 improves the lifespan of SMA mice

To find whether JNK inhibition improves the lifespan of SMA mice, we examined survival of mice untreated (SMA), treated with vehicle (SMA + DMSO) or with JNK inhibitor SP600125 (SMA + SP600125) using Kaplan–Meier analysis. Comparison of median survival of combined males and females shows statistically significant 2.0-fold increase (Log-rank test, *P* < 1.0 × 10^−10^, *n* = 20/group) in the lifespan of SMA + SP600125 (17.0 days) compared with control SMA (8.0 days) and SMA + DMSO (8.5 days) mice (Fig. 3L). JNK inhibition also resulted in >2.0-fold increase in average (male + female) survival of SMA + SP600125 mice (mean ± SEM) (17.25 ± 0.323, *n* = 20) (ANOVA, *P* < 1.0 × 10^−10^) days compared with SMA (7.60 ± 0.67, *n* = 20) and SMA + DMSO (9.00 ± 0.71, *n* = 20) (Fig. 3J–L and O). However, the comparison and statistical analysis of survival of SMA mice (untreated) and SMA treated with DMSO (SMA + DMSO) did not show statistically significant difference for males (*P* = 0.4616), females (*P* = 0.2458), and males + females (*P* = 0.1623), suggesting that DMSO (vehicle) did not influence the severity of the disease and did not affect survival of SMA mice. The survival data are consistent with the overall growth data that also did not show statistically significant difference in male and female SMA and SMA + DMSO mice. The sex-based analysis shows average survival of SMA + SP600125 males (17.09 ± 0.41, *n* = 11, *P* = 1.20 × 10^−9^) and median survival (17.0 days) are higher than SMA males average (7.50 ± 0.88, *n* = 10) and median (8.0 days) survival and SMA + DMSO males average (8.50 ± 0.99, *n* = 10) and median (7.5 days) survival (Fig. 3J and M). Average survival of SMA + SP600125 females (17.44 ± 0.53, *n* = 9, *P* = 2.20 × 10^−7^) and median survival (17.0 days) are also higher than SMA females average (7.70 ± 1.06, *n* = 10) and median (8.0 days) and SMA + DMSO average (9.50 ± 1.05, *n* = 10) and median (9.0 days) survival (Fig. 3K and N). Comparison of average lifespan among SMA + SP600125 males (2.13-fold) and females (2.02-fold) shows JNK inhibition results in similar improvement in the lifespan of both female and male mice (Fig. 3M–O). These data are in alignment with similar increase in growth in male (∼75%) and female (∼53%) mice treated with JNK inhibitor SP600125 (Fig. 3D–F). Notably, 5 days increase in maximum survival (∼34%) of SMA + SP600125 mice includes 4 days of growth (reduced severity), meaning ∼80% increase in the lifespan with reduced severity of disease in JNK inhibitor treated SMA mice (Fig. 3J–L). Analysis of minimum survival shows marked increase (5.0-fold, *P* < 1.0 × 10^−10^) in the initial survival of SMA + SP600125 mice compared with control SMA mice. These data show that every mouse treated with JNK inhibitor, male or female (*n* = 20), survived for at least 15 days after birth compared with SMA and SMA + DMSO that started dying at the age of 3 days (Fig. 3P–R). These data suggest that JNK inhibition ameliorates postnatal SMA disease severity, markedly decreases early mortality and significantly increases the lifespan of SMA mice.

### JNK inhibition with SP600125 improves gross motor function and muscle strength of SMA mice

**Figure 4:**
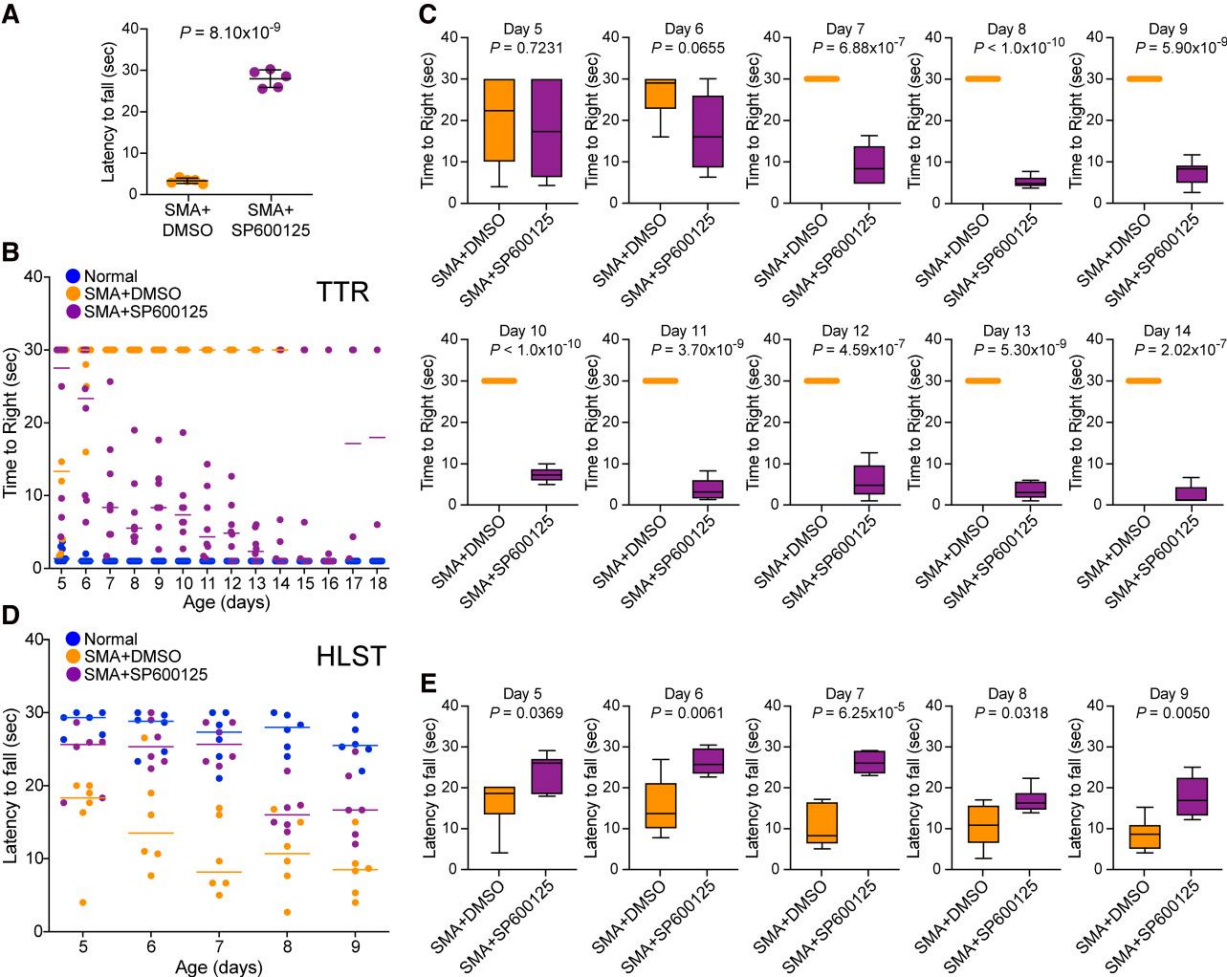
**Pharmacological JNK inhibition improves the gross motor function and muscle strength of SMA mice.** (**A**) JNK inhibition improves the ability to stand on paws and walk. Improvement in the ability of SMA mice to stand on paws and walk was determined by measuring the time of falling after walking on four paws and was recorded in 10-day-old SMA + DMSO and SMA + SP600125 littermates. Comparison of latency to fall (seconds) from paws is presented as a scatter plot with individual data points that represent an average of three recordings per pup/group/day showing that all pups treated with drug compound were able to stand and walk for an average time of ∼28 s, suggesting an improvement in gross motor function. Statistical analysis of (mean ± SEM, *n* = 5 mice/group, male + female) (seconds) between SMA + DMSO (3.13 ± 0.29) and SMA + SP600125 (27.73 ± 0.95), *P* = 8.10 × 10^−9^ (unpaired *t*-test, two-tailed) for 10-day-old mice shows a marked improvement in the ability of SMA mice treated with JNK inhibitor to stand on paws and walk (see Supplementary Videos 1–3). (**B**) Mice righting reflexes were examined for 5–18-day-old normal (blue), SMA + DMSO (orange), and SMA + SP600125 (purple) littermates. TTR with a time limit of 30 s for testing and an average of 3 recordings per pup/day/group are plotted. Data were collected from three groups starting with *n* = 6 mice/group, which are presented as a scatter plot. (**C**) Improvement in the motor function (righting) is demonstrated by an age-dependent increase in the ability of mice to right faster upon treatment with JNK inhibitor SMA + SP600125 compared with control SMA mice treated with DMSO (SMA + DMSO). The results are shown as box-and-whisker plots (median, min, max) starting from postnatal day 5 (PND5) [SMA + DMSO (22.34, 4.0, 30.0) and SMA + SP600125 (17.30, 4.33, 30.0)], to PND14 [SMA + DMSO (30.0, 30.0, 30.0) and SMA + SP600125 (1.16, 1.0, 6.67)]. Statistical analysis (mean ± SEM) shows a slight improvement in TTR at PND5 (*P* = 0.7231) and PND6 (*P* = 0.0655) that is not statistically significant, however analysis of PND7 to PND14 shows a significant improvement in the righting ability and TTR of SMA + SP600125 mice from PND7 [SMA + SP600125 (9.22 ± 1.89, *n* = 6), unpaired *t*-test, *P* = 6.88 × 10^−7^], PND8 (5.16 ± 0.58), *P* < 1.0 × 10^−10^, PND9 (7.48 ± 1.24), *P* = 5.90 × 10^−9^, PND10 (7.38 ± 0.74), *P* < 1.0 × 10^−10^, PND11 (3.83 ± 1.07), *P* = 3.70 × 10^−9^, PND12 (5.83 ± 1.71), *P* = 4.59 × 10^−7^, PND13 (3.44 ± 0.81), *P* = 5.30 × 10^−9^, and PND14 (2.44 ± 0.94), *P* = 2.02 × 10^−7^ compared with SMA + DMSO (30.0 ± 00.0) from PND7-14. (**D**) The HLST shows that JNK inhibition increases muscle strength in mice with SMA. Littermates of 5–9 days of age were hung on both hind legs on the edge of a 50 ml plastic conical tube and time (seconds) was recorded until fall from the edge of the tube. Latency to fall (mean ± SEM, 6 mice/group, male + female) is shown as a scatter plot and (**E**) as box-and-whisker plots with median with interquartile range (median, min, max) for each time point (day), PND5 [SMA + DMSO (18.34, 4.0, 20.0) and SMA + SP600125 (25.64, 17.67, 28.67)], PND6 [SMA + DMSO (13.5, 7.67, 26.56) and SMA + SP600125 (25.34, 22.33, 30.0)], PND7 [SMA + DMSO (8.17, 5.0, 16.94) and SMA + SP600125 (25.67, 22.67, 28.67)], PND8 [SMA + DMSO (10.69, 2.67, 16.77) and SMA + SP600125 (16.0, 13.67, 22.0)], and PND9 [SMA + DMSO (8.50, 4.0, 15.03) and SMA + SP600125 (16.67, 12.0, 24.67)]. Statistical analysis (*t*-test, unpaired) shows a marked and statistically significant increase in the hanging time for JNK inhibitor treated SMA mice compared with DMSO treated SMA mice with increasing age; PND5 [SMA + SP600125 (23.66 ± 1.85) and SMA + DMSO (16.17 ± 2.50), *P* = 0.0369], PND6 [SMA + SP600125 (25.89 ± 1.29) and SMA + DMSO (15.15 ± 2.82), *P* = 0.0061], PND7 [SMA + SP600125 (25.78 ± 1.12) and SMA + DMSO (10.16 ± 2.09), *P* = 6.25 × 10^−5^], PND8 [SMA + SP600125 (16.61 ± 1.22) and SMA + DMSO (10.58 ± 2.09), *P* = 0.0318], and PND9 [SMA + SP600125 (17.44 ± 1.95) and SMA + DMSO (8.44 ± 1.56), *P* = 0.0050] shows a gradual increase in the muscle strength of JNK inhibitor treated mice compared with control SMA mice.

Defects in gross motor functions and the loss of ability to stand and walk are features of clinical phenotypes linked to severe SMA. To determine the beneficial effects of JNK inhibition on these relevant features, we examined and compared the gross motor functions such as ability stand on four paws, ability to right and walk. To evaluate leg strength after JNK inhibition, we compared the ability of treated and untreated SMA mice to stand on four paws with a test time limit of 30 s.^16,26^ The ability to stand on four paws and walk (time in seconds) was improved in 10-day-old SMA + SP600125 mice (27.73 ± 0.77, *n* = 5, *P* = 8.10 × 10^−9^) compared with control (SMA + DMSO) (3.20 ± 0.25) (Fig. 4A). Video clips of 6-, 10-, and 15-day-old littermates treated with DMSO (black dots) and SP600125 (red dots) (Supplementary Videos 1–3) show that control SMA mice have severe defects in their ability to stand on four paws and right themselves, in addition to being unable to walk. In contrast, SMA + SP600125 mice were able to walk and when falling, they were able to right themselves within a few seconds and continue to walk. These data show that JNK inhibition results in marked improvement in the ability of SMA mice to walk.

To gain insight into the gross motor function during the treatment of SMA mice with the JNK inhibitor, we examined TTR for 5–18-day-old mice with a test time limit of 30 s.^16,26^ Analysis of TTR at PND5 (*P* = 0.7231) and PND6 (*P* = 0.0655) did not show improvement in the motor function (Fig. 4C). Notably, analysis of TTR shows statistically significant improvement in the ability to right (gross motor function) of SMA + SP600125 compared with SMA + DMSO mice starting from PND7 [SMA (30.00 ± 0.00, *n* = 6) and SMA + SP600125 (9.223 ± 1.89, *n* = 6), *P* = 6.88 × 10^−7^] to PND14 [SMA (30.00 ± 0.00, *n* = 3) and SMA + SP600125 (2.445 ± 0.94, *n* = 6), *P* = 2.02 × 10^−7^] (Fig. 4B and C). All SMA littermates in this assay were dead by the Day 14. However, JNK inhibitor-treated mice showed continued improvement with aging in their ability to right until PND17 (16.42 ± 7.86, *n* = 4) (Fig. 4C). The ability to right for SMA + SP600125 mice started to decline from PND18, and mice were unable to right by PND19. The increase in ability to right with reduction in TTR of JNK inhibitor treated mice compared with control SMA mice, with age-dependent increase, suggests gradual improvement in the gross motor function and reduction in disease severity of SMA + SP600125 mice with a single dose of treatment on alternate days with the drug compound.

A representative video clip of 15-day-old normal and JNK inhibitor-treated littermates shows that SMA + SP600125 (red mark on tail) mouse was able to right itself and walk actively (Supplementary Video 3). Improvement in multiple motor functions of JNK inhibitor-treated mice, such as the ability to right themselves, stand on four paws, and walk in the late stages of lifespan, points to the possibility of increased muscular strength. To test muscle strength, we used HLST to evaluate improvement in the proximal hind-limb muscle strength.^16,26,51,52^ Mice with age range of 5–9 days were hanged on both hind legs on the edge of a 50 ml plastic conical tube and time was recorded until their fall as described under behavioural analysis in methods section. Comparison of latency to fall (seconds) between SMA + SP600125 (23.66 ± 1.85, *n* = 6) and SMA + DMSO (16.17 ± 2.50, *n* = 6) at PND5 shows statistically significant difference (*P* = 0.0369) suggesting that just two doses of JNK inhibitor resulted in notable increase in muscle strength (Fig. 4D and E). Further analysis showed marked increase in hanging time of SMA + SP600125 compared with SMA + DMSO mice at PND6 [SMA + DMSO (15.15 ± 2.82) and SMA + SP600125 (25.89 ± 1.291, *P* = 0.0061)], PND7 [SMA + DMSO (10.16 ± 2.09) and SMA + SP600125 (25.78 ± 1.124, *P* = 6.25 × 10^−5^)]. The hanging time at PND8 [SMA + DMSO (10.58 ± 2.09) and SMA + SP600125 (16.61 ± 1.22, *P* = 0.0318)] and PND9 [SMA + DMSO (8.448 ± 1.56) and SMA + SP600125 (17.44 ± 1.95, *P* = 0.0050)] for JNK inhibitor treated mice reduced but showed statistically significant increase in muscle strength with peak performance at PND7 (Fig. 4D and E). The increase in hanging time on hindlimbs suggests improvement in hindlimbs muscle strength of JNK inhibitor-treated mice compared with control SMA mice. Together, these data suggest that JNK inhibition improves gross motor function and muscle strength of SMA mice.

### Treatment with pan-JNK inhibitor AS601245 improves overall growth of SMA mice

To consolidate the notion on whether pharmacological inhibition of JNK is a viable option for SMA treatment, we tested another pan-JNK inhibitor AS601245 with IC_50_ of 150 nM (JNK1), 220 nM (JNK2) and 70 nM (JNK3), with moderate potency of inhibiting JNK1, lower potency for JNK2 and higher potency for JNK3 and, importantly, shows low toxicity for cultured primary neurons (Figs 1 and 2). SMA mice treated with inhibitor AS601245 were healthier, able to stand and walk compared with control SMA mice treated with vehicle between the ages of 6–18 days. The JNK inhibition with AS601245 also resulted in improvement of the overall growth, health and gross motor function of SMA mice by PND6 as shown in video clips of littermates, one pup treated with DMSO (black dot on tail and head) and two treated with AS601245 (one and two red dots) (Supplementary Video 4). Additional video clips of mice at the ages of 14 and 17 days with normal (black dot on tail) and AS601245 (one red dot on tail and no marking) show that SMA mice treated with JNK inhibitor were able to right and walk (Supplementary Videos 5 and 6). Because the comparison of SMA and SMA + DMSO did not show any statistically significant differences in the overall growth and survival between SMA and SMA mice treated with DMSO (SMA + DMSO) (Fig. 3), we used SMA + DMSO as control mice for evaluating the effects of other two JNK inhibitors.

**Figure 5:**
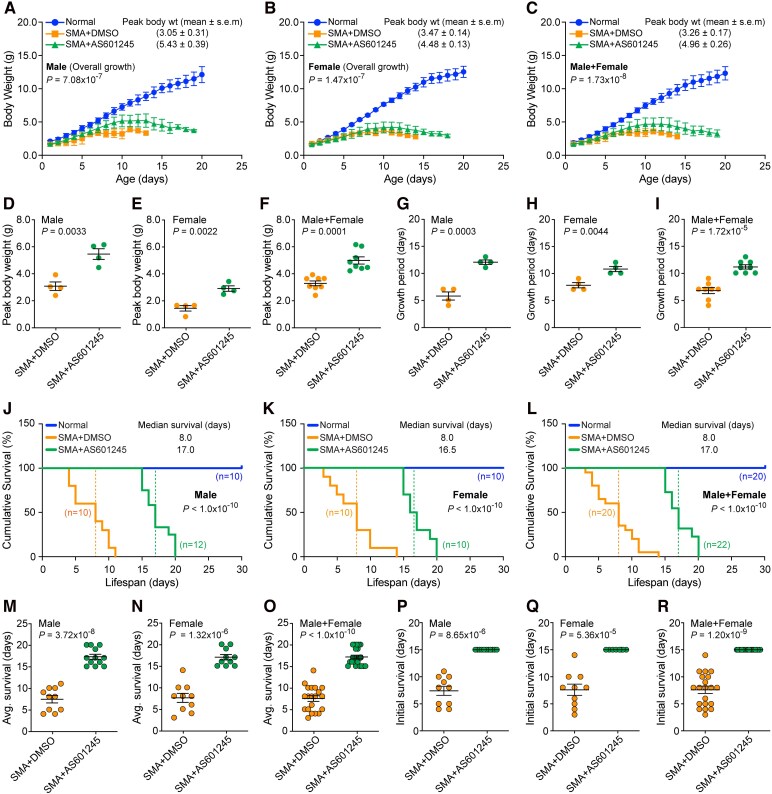
**Pharmacological inhibition of JNK with pan-JNK inhibitor AS601245 improves the overall growth and survival of SMA mice.** SMA (SMAD7) mice littermates starting at PND2 were injected IP with a single dose of vehicle DMSO or JNK inhibitor AS601245 (20 mg/kg) followed by an every-other-day schedule. Amelioration of disease severity, improvement in growth and increase in the lifespan of mice were examined. (**A–C**) Pharmacological inhibition of JNK improves the growth of SMA mice. Overall growth (body weight in g) curves of normal (blue), SMA + DMSO (orange) and SMA + AS601245 (green) mice. Body weights recorded every day are presented as growth curves with mean ± SD, *n* = minimum 3 mice/group for individual male and female groups and combined (male + female) group. Statistical analysis (ANOVA) shows marked increase in overall growth of mice treated with AS601245, male (*P* = 7.08 × 10^−7^), female (*P* = 1.47 × 10^−7^) and male + female (*P* = 1.73 × 10^−8^). (**D–F**) Average peak body weight (grams) shown as scatter plots with mean ± SEM (*n* = 4/sex/group). Each data point represents a biological replicate. (**D**) Analysis of these data show statistically significant increase in average peak body weight (grams) (mean ± SEM) of SMA + AS601245 males (5.43 ± 0.39, *n* = 4) compared with SMA + DMSO (3.05 ± 0.31) males (*P* = 0.0033, unpaired *t*-test), which shows ∼78% increase in body weight of SMA + AS601245 compared with SMA + DMSO males. (**E**) SMA + AS601245 females (4.48 ± 0.13) compared with SMA + DMSO (3.47 ± 0.14) females (*P* = 0.0022) that show ∼29% increase in peak body weight SMA + AS601245 females. (**F**) Comparison of combined (male + female) peak body weight in SMA + AS601245 (4.96 ± 0.26, *n* = 8) with SMA + DMSO (3.26 ± 0.17) (*P* = 0.0001) shows an average peak body weight increase of ∼52% in SMA + AS601245 mice. (**G–I**) JNK inhibition improves postnatal growth period (days) (mean ± SEM, *n* = 4/sex/group) of SMA mice. Growth period of (**G**) SMA + AS601245 (12.00 ± 0.40) males compared with SMA + DMSO (5.75 ± 0.75) male mice shows ∼109% (*P* = 0.0003) increase in postnatal growth period, (**H**) SMA + AS601245 females (10.75 ± 0.47) compared with SMA + DMSO (7.75 ± 0.48) female mice shows ∼39% (*P* = 0.0044) increase in postnatal growth period, (**I**) comparison of combined (males + females), SMA + AS601245 (11.13 ± 0.39) to SMA + DMSO (6.75 ± 0.55) males + females shows a statistically significant (*P* = 1.72 × 10^−5^) increase in the postnatal growth period. Growth analysis was blinded with colour coding. (**J–R**) Pharmacological inhibition of JNK improves the survival of SMA mice. (**J–L**) Kaplan–Meier survival curves of normal (blue), SMA + DMSO (orange) and SMA + AS601245 (green) mice littermates. Vertical dotted lines show median survival. Survival analysis show ∼2.13-fold increase in median survival of SMA mice treated with drug AS601245 compared with control SMA + DMSO mice (Log-rank test, *P* < 1.0 × 10^−10^). (**M–O**) The scatter plots show average survival (days) (mean ± SEM) of SMA mice untreated (SMA), treated with DMSO (SMA + DMSO) and JNK inhibitor AS601245 (SMA + AS6001245). (**M**) Sex-based analysis shows that the average survival of SMA + AS601245 males (17.25 ± 0.57, *n* = 12) (*P* = 3.72 × 10^−8^) is higher than SMA + DMSO (7.40 ± 0.84, *n* = 10) males. (**N**) Average survival of SMA + AS601245 females (17.0 ± 0.63, *n* = 10) (*P* = 1.32 × 10^−6^) is also higher compared with SMA + DMSO (7.60 ± 1.03, *n* = 10) females. (**O**) Statistical analysis of male and female survival shows an increase in average survival (mean ± SEM) days of SMA + AS601245 (17.14 ± 0.41, *n* = 22) (*t*-test, unpaired *P* < 1.0 × 10^−10^) compared with SMA + DMSO (7.50 ± 0.65, *n* = 20) mice. (**P–R**) Scatter plots show a marked increase (5-fold) in initial (minimum) survival of SMA mice treated with JNK inhibitor compared with SMA + DMSO mice. All males and females (*n* = 22) treated with AS601245 survived at least 15 days compared with SMA males and females that survived at least 3 days. Growth and survival analyses were blinded with colour coding. Sex and genotypes were confirmed by PCR-based method after euthanasia of pups.

The sex-based overall growth (body weight) analysis of mice normal (blue), SMA + DMSO (orange) and SMA + AS601245 (green) show statistically significant increase in the growth of male (*P* = 7.08 × 10^−7^), female (*P* = 1.47 × 10^−7^), and male + female (*P* = 1.73 × 10^−8^) SMA mice treated with AS601245 compared DMSO treated mice (Fig. 5A–C). Overall growth analysis of SMA littermates shows that *in vivo* JNK inhibition with drug compound AS601245 increased average (mean ± SEM, *n* = 4) peak body weight (grams) in males SMA + AS601245 (5.43 ± 0.39, *P* = 0.0033) compared with SMA + DMSO (3.05 ± 0.31) males and in females SMA + AS601245 (4.48 ± 0.13, *P* = 0.0022) compared with SMA + DMSO (3.47 ± 0.14) females (Fig. 5A and B). Comparison of combined (male + female) peak body weight of SMA + AS601245 (4.96 ± 0.26, *n* = 8, *P* = 0.0001) with SMA + DMSO (3.26 ± 0.17) mice show statistically significant improvement in the growth of SMA mice treated with JNK inhibitor (SMA + AS601245) (Fig. 5D–F). Further analysis of data shows the overall growth increase in SMA + AS601245 males is ∼78% (*P* = 0.0033) and in females is ∼29% (*P* = 0.0022) compared with DMSO treated SMA males and females, respectively (Fig. 5D–E). These data show better improvement in the body weight of male mice compared with female mice upon treatment with JNK inhibitor (Fig. 5D–F). JNK inhibition also increased the growth period of male mice from (5.75 ± 0.75, *n* = 4) (SMA + DMSO) to (12.00 ± 0.40, *n* = 4) (SMA + AS601245) days and female mice from (7.75 ± 0.48, *n* = 4) (SMA + DMSO) to (10.75 ± 0.47, *n* = 4) (SMA + AS601245) days that shows ∼109% increase (*P* = 0.0003) in males and ∼39% increase (*P* = 0.0044) in females in the postnatal growth periods, respectively (Fig. 5G and H). The increase in growth period of combined male + female mice with AS6001245 compared with DMSO treated mice is highly significant (1.72 × 10^−5^) (Fig. 5I). Together, these data suggest that *in vivo* JNK inhibition with AS601245 show better improvement in overall growth and postnatal growth of period of SMA male mice compared with female mice. These sex-based data suggest that AS601245 may be a better drug (JNK inhibitor) for improving body weight (overall growth) in males with SMA.

### JNK inhibition with AS601245 improves the lifespan of SMA mice

The Kaplan–Meier analysis of median survival of mice treated with vehicle (SMA + DMSO) and JNK inhibitor AS601245 (SMA + AS601245) shows JNK inhibition resulted in ∼2.13-fold increase in combined (male + female) survival of SMA + AS601245 mice (Log-rank test, *P* < 1.0 × 10^−10^). The sex-based comparison of median survival of males (∼2.13-fold, *P* < 1.0 × 10^−10^) and females (∼2.06-fold, *P* < 1.0 × 10^−10^) also shows significant increase in the lifespan of SMA + AS601245 compared with control SMA + DMSO mice (Fig. 5J and K). The analysis of average survival (mean ± SEM) of SMA + AS601245 males (17.25 ± 0.57, *n* = 12, *P* = 3.72 × 10^−8^) and median survival (17.0 days) is higher than SMA + DMSO males average (7.40 ± 0.84, *n* = 10) and median (8.0 days) survival (Fig. 5J and M). Average survival of SMA + AS601245 females (17.00 ± 0.63, *n* = 10, *P* = 1.32 × 10^−6^) and median survival (16.5 days) is also higher than SMA + DMSO average (7.60 ± 1.03, *n* = 10) and median (8.0 days) survival (Fig. 4K and N). Average survival of combined (male + female) SMA + AS601245 (17.14 ± 0.41, *n* = 22, *P* < 1.0 × 10^−10^) days compared with SMA + DMSO (7.50 ± 0.65, *n* = 20) is highly significant (Fig. 5L and O). Comparison of average lifespan among SMA + AS601245 males (2.36-fold) and females (2.30-fold) shows JNK inhibition results in similar improvement in the lifespan of both female and male mice (Fig. 5M–O). Comparison of growth and survival data show that increase in body weight of male (∼109%) and female (∼39%) mice treated with JNK inhibitor AS601245 (Fig. 5D–F) made only a slight difference in the increase of lifespan of males (2.36-fold) compared with females (2.30-fold) (Fig. 5J–O). However, overall increase in the lifespan (∼2.30-fold, *P* < 1.0 × 10^−10^) of male and female SMA mice treated with AS601245 compared control is highly significant (Fig. 5M–O). Notably, 6 days increase in maximum survival of SMA + AS601245 mice (∼43% increase in max survival) includes five additional days of growth (reduced severity) and results in about ∼83% increase in the lifespan with reduced severity of disease (Fig. 5J–L). Analysis of minimum survival shows marked increase (5.0-fold, *P* < 1.20 × 10^−9^) in the initial survival of SMA + AS601245 mice compared with control (SMA + DMSO) mice. These data show that every mouse treated with JNK inhibitor, male or female (*n* = 20), survived for at least 15 days after birth compared with control (SMA + DMSO) mice that started dying at the age of 3 days (Fig. 5P–R). These data suggest that JNK inhibition reduced postnatal disease severity, markedly decreased early mortality and increased the lifespan of SMA mice.

### JNK inhibition with AS601245 improves gross motor function and muscle strength of SMA mice

**Figure 6:**
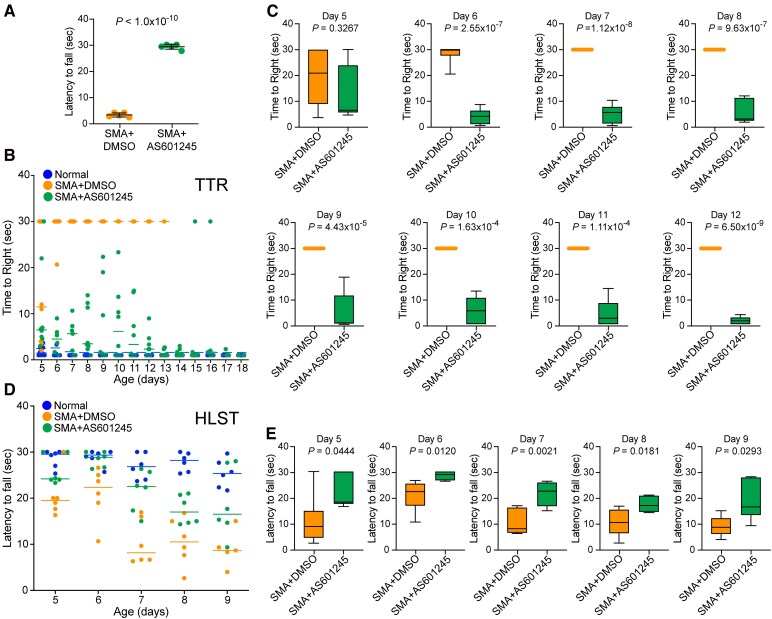
**Pharmacological JNK inhibition with AS601245 improves the gross motor function and muscle strength of SMA mice.** (**A**) JNK inhibition improves the ability to stand on paws and walk. Improvement in the ability of SMA mice to stand on paws and walk was determined by measuring the time of falling after walking on four paws and was recorded in 10-day-old SMA + DMSO and SMA + AS601245 littermates. Comparison of latency to fall (seconds) from paws is presented as a scatter plot with individual data points that represent an average of three recordings per pup/group/day showing all pups treated with the drug compound were able to stand and walk for an average time of >29 s suggesting an improvement in the gross motor function. Statistical analysis of (mean ± SEM, *n* = 5 mice/group, male + female) (seconds) between SMA + DMSO (3.2 ± 0.34) and SMA + AS601245 (29.27 ± 0.42), *P* < 1.0 × 10^−10^ (unpaired *t*-test, two-tailed) for 10-day-old mice shows a marked improvement in the ability of SMA mice treated with JNK inhibitor to stand on paws and walk (see Supplementary Videos 4–6). (**B**) Mice righting reflexes were examined for 5–18-day-old normal (blue), SMA + DMSO (orange), and SMA + AS601245 (green) littermates. TTR with a time limit of 30 s for the test and an average of 3 recordings per pup/day/group are plotted. Data were collected from three groups starting with *n* = 6 mice/group, which are presented as a scatter plot. JNK inhibitor treated mice show improvement in their ability to right until PND18. (**C**) Improvements in the motor function (righting) is demonstrated by an age-dependent increase in the ability of mice to right faster upon treatment with JNK inhibitor SMA + AS601245 compared with control SMA mice treated with DMSO (SMA + DMSO). The results are shown as box-and-whisker plots (median, min, max) starting from PND5 [SMA + DMSO (21.0, 4.0, 30.0) and SMA + AS601245 (6.83, 5.0, 30.0)], to PND12 [SMA + DMSO (30.0, 30.0, 30.0) and SMA + AS601245 (2.33, 1.0, 4.67)]. Statistical analysis (mean ± SEM) shows a slight improvement in the TTR at PND5 [SMA + AS601245 (12.83 ± 4.29, *n* = 6)] compared with control [SMA + DMSO (19.50 ± 4.82)] but it is not statistically significant (unpaired *t*-test, *P* = 0.3267). Analysis of righting ability from PND6 to PND12 shows a statistically significant improvement in the TTR of JNK inhibitor treated mice; PND6 [SMA + AS601245 (4.44 ± 1.21, *n* = 6), SMA + DMSO (28.45 ± 1.55, *n* = 6) *P* = 2.55 × 10^−7^], PND7 (5.49 ± 1.45), *P* = 1.12 × 10^−8^, PND8 (5.99 ± 1.85), *P* = 9.63 × 10^−7^, PND9 (5.66 ± 2.99), *P* = 4.43 × 10^−5^, PND10 (6.38 ± 2.21), *P* = 1.63 × 10^−4^, PND11 (5.11 ± 2.19), *P* = 1.11 × 10^−4^ and PND12 (2.44 ± 0.57), *P* = 6.50 × 10^−9^ compared with SMA + DMSO (30.0 ± 0.0) from PND7-12. (**D**) The HLST shows that JNK inhibition increases muscle strength in mice with SMA. Littermates of 5–9 days of age were hung on both hind legs on the edge of a 50 ml plastic conical tube and time (seconds) was recorded until fall from the edge of the tube. Latency to fall (mean ± SEM, 6 mice/group, male + female) is shown as a scatter plot and (**E**) as box-and-whisker plots with median with interquartile range (median, min, max) for each time point (day), PND5 [SMA + DMSO (19.50, 16.33, 30.0) and SMA + AS601245 (24.17, 23.33, 30.0)], PND6 [SMA + DMSO (22.34, 10.67, 26.58) and SMA + AS601245 (28.84, 26.33, 30.0)], PND7 [SMA + DMSO (8.17, 6.33, 16.94) and SMA + AS601245 (22.50, 15.0, 26.33)], PND8 [SMA + DMSO (10.52, 2.67, 16.77) and SMA + AS601245 (17.0, 14.33, 21.0)], and PND9 [SMA + DMSO (8.67, 4.0, 15.03) and SMA + AS601245 (16.50, 9.33, 28.0)]. Statistical analysis (*t*-test, unpaired) shows marked and statistically significant increase in hanging time (seconds) for JNK inhibitor treated compared with DMSO treated SMA mice with increasing age; PND5 [SMA + AS601245 (25.94 ± 1.29) and SMA + DMSO (20.50 ± 1.98), *P* = 0.0444], PND6 [SMA + AS601245 (28.45 ± 0.65) and SMA + DMSO (20.99 ± 2.34), *P* = 0.0120], PND7 [SMA + AS601245 (21.56 ± 1.84) and SMA + DMSO (10.38 ± 1.99), *P* = 0.0021], PND8 [SMA + AS601245 (17.45 ± 1.27) and SMA + DMSO (10.52 ± 2.09), *P* = 0.0181], and PND9 [SMA + AS601245 (18.78 ± 3.07) and SMA + DMSO (9.07 ± 1.76), *P* = 0.0293] shows gradual increase in the muscle strength of JNK inhibitor treated mice compared with control SMA mice.

To determine the beneficial effects of JNK inhibition with AS601245 on clinically relevant features of gross motor functions such as ability to stand and walk, we examined and compared the gross motor functions; ability to stand on four paws, ability to right and walk. The ability to stand on four paws and walk (time in seconds) was improved in 10-day-old SMA + AS601245 mice (29.26 ± 0.34, *P* < 1.0 × 10^−10^) compared with control (SMA + DMSO) (3.20 ± 0.28) (Fig. 6A). Video clips of 6-, 14-, and 17-day-old littermates treated with DMSO (black dots) and AS601245 (red dots) (Supplementary Videos 4–6) show that control SMA mice have severe inability to stand on four paws and were unable to right themselves and walk. In contrast, SMA + AS601245 mice were able to walk and, if fallen, then they were able to right themselves within a few seconds and continued to walk. Notably, mice treated with AS601245 were able to walk up to the age of 18 days suggesting a remarkable improvement in the gross motor function that could be due to increase in muscle strength.

The analysis of TTR at PND5 did show some improvement in SMA + AS601245 (12.83 ± 4.29, *n* = 6) compared with SMA + DMSO (19.50 ± 4.829) but it was not statistically significant (*P* = 0.3267). However, a steady and statistically significant improvement in the TTR of SMA + AS601245 compared with SMA + DMSO mice was noted starting from PND6 [SMA (28.45 ± 1.55, *n* = 6) and SMA + AS601245 (4.44 ± 1.21, *n* = 6), *P* = 2.55 × 10^−7^] to PND12 [SMA (30.00 ± 0.00, *n* = 3) and SMA + AS601245 (2.44 ± 0.57, *n* = 6), *P* = 6.50 × 10^−9^] (Fig. 6B and C). Interestingly, JNK inhibitor treated mice continued to show improvement in their ability to right until PND18 with TTR time of ∼2–3 s, which is comparable to normal mice (Fig. 6C). The ability to right and walk of SMA mice treated with AS601245 started to decline from PND18, and mice were unable to right itself a day or two before approaching the end of their lifespan. The improved ability to right of SMA mice treated with JNK inhibitor compared with control SMA mice, with increasing age, suggests gradual improvement with aging in the gross motor function and decrease in the severity of the SMA disease. A representative video clip of 17-day-old littermates treated with AS601245 JNK inhibitor shows they were able to right themselves and walk actively (Supplementary Video 6). These data suggest that JNK inhibition helps maintain motor activity and mobility of SMA mice with aging.

Improvement in the gross motor functions of SMA mice treated with AS601245 points to the possibility of increase in muscular strength. The HLST analysis of SMA + AS601245 (25.94 ± 1.29, *n* = 6) and SMA + DMSO (20.50 ± 1.98, *n* = 6) at PND5 shows a statistically significant difference (*P* = 0.0444), suggesting that the initial two doses of JNK inhibitor treatment resulted in moderate increase in muscle strength (Fig. 6D and E). Further analysis of muscle strength showed marked increase in muscle strength of SMA + AS601245 compared with SMA + DMSO mice at PND6 [SMA + DMSO (20.99 ± 2.34) and SMA + AS601245 (28.45 ± 0.65, *P* = 0.0120)], PND7 [SMA + DMSO (10.38 ± 1.99) and SMA + AS601245 (21.56 ± 1.84, *P* = 0.0021)]. The hanging time at PND8 [SMA + DMSO (10.52 ± 2.09) and SMA + AS601245 (17.45 ± 1.27, *P* = 0.0181)] and PND9 [SMA + DMSO (9.07 ± 1.76) and SMA + AS601245 (18.78 ± 3.07, *P* = 0.0293)] for JNK inhibitor treated mice reduced but still shows statistically significant increase in muscle strength (Fig. 6D and E). Increase in hind-limb hanging time suggest improvement in hind-limb muscle strength of AS601245 treated mice compared with control SMA mice. Together, these data suggest that JNK inhibition with AS601245 also improves gross motor function and muscle strength of SMA mice.

### Treatment with high potency JNK3 inhibitor SR12519 improves overall growth of SMA mice

We previously showed that the genetic inhibition of JNK3, neuron-specific isoform, in SMA mice resulted in systemic amelioration of disease severity.^26^ To test whether pharmacological inhibition using JNK3-specific inhibitor results in similar amelioration of the disease, we examined the effect of JNK3-specific inhibitor with high potency and selectivity for JNK3, SR12519 with IC_50_ of 21 nM (JNK1), 66 nM (JNK2), and 15 nM (JNK3).^50^ Because of higher overall potency of SR12519 against all JNK isoforms, including highest potency for JNK3 and lower cell (neuron) viability (∼80%) compared with cell viability of SP600125 and AS601245 (∼90%) at 1.0 mM, we used lower dose (10 mg/kg) to minimize *in vivo* toxicity (Fig. 1G, Supplementary Table 1). Similar and high potency against JNK1 and JNK3 of SR12519 suggest that this inhibitor may be able to target both JNK1 and JNK3 with similar efficacy and provide insight into combined effective inhibition of JNK1 and JNK3 in the context of impact of JNK inhibition in the amelioration of SMA disease severity. As we anticipated, SMA mice treated with JNK3-specific inhibitor SR12519 were healthier, able to stand and walk compared with control SMA mice treated with vehicle between the ages of 6–15 days. Initial visual observations (videography) suggested that selective inhibition of JNK3 also results in improving the overall growth and gross motor function of SMA mice as shown in video clips of littermates treated with DMSO and SR12519 at the ages of 8 and 13 days. The DMSO treated mice were unable to right and could not walk. Notably, mice treated with inhibitor were healthy, showed improved growth, were able to right and walk at the ages of 8, 10, and 13 days (Supplementary Videos 7–9).

**Figure 7:**
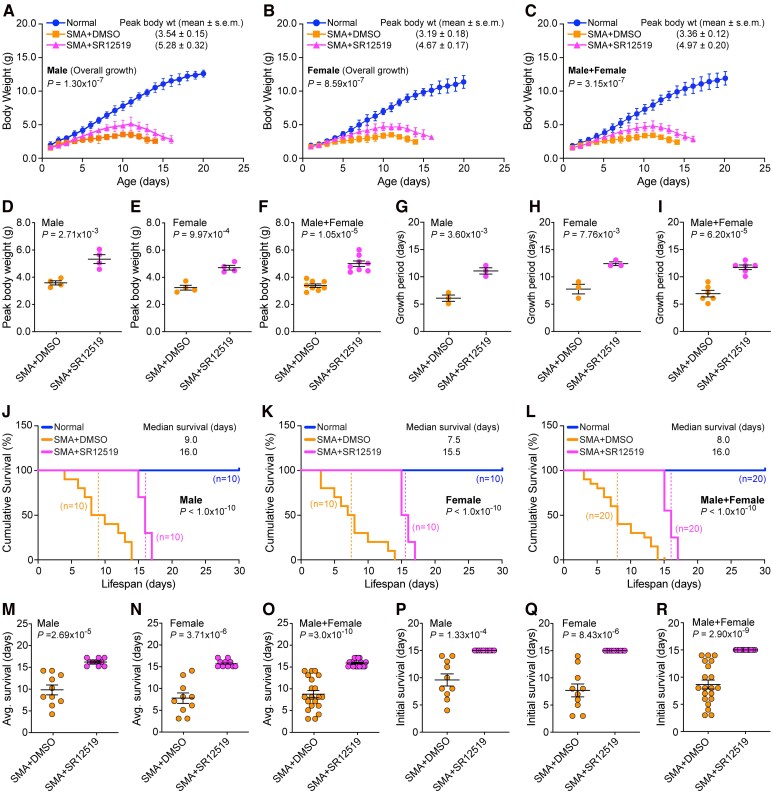
**Pharmacological inhibition of JNK with pan-JNK inhibitor SR12519 improves the overall growth and survival of SMA mice.** SMA (SMAD7) mice littermates starting at PND2 were injected IP with a single dose of vehicle DMSO or JNK inhibitor SR12519 (20 mg/kg) followed by an every-other-day schedule. Amelioration of disease severity, improvement in growth and increase in the lifespan of mice were examined. (**A–C**) Pharmacological inhibition of JNK improves the growth of SMA mice. Overall growth (body weight in g) curves of normal (blue), SMA + DMSO (orange) and SMA + SR12519 (magenta) mice. Body weights recorded every day are presented as growth curves with mean ± SD, *n* = minimum 3 mice/group for individual male and female groups and combined (male + female) group. Statistical analysis (ANOVA) shows marked increase in overall growth of mice treated with SR12519, male (*P* = 1.30 × 10^−7^), female (*P* = 8.59 × 10^−7^) and male + female (*P* = 3.15 × 10^−8^). (**D–F**) Average peak body weight (grams) shown as scatter plots with mean ± SEM (*n* = 4/sex/group). Each data point represents a biological replicate. (**D**) Analysis of these data show statistically significant increase in average peak body weight (grams) (mean ± SEM) of SMA + SR12519 males (5.28 ± 0.32) compared with SMA + DMSO (3.54 ± 0.15) males (*P* = 2.71 × 10^−3^, unpaired *t*-test), which shows ∼49% increase in body weight of SMA + SR12519 compared with SMA + DMSO males. (**E**) SMA + SR12519 females (4.67 ± 0.17) compared with SMA + DMSO (3.19 ± 0.18) females (*P* = 9.97 × 10^−4^) that show ∼46% increase in peak body weight SMA + SR12519 females. (**F**) Comparison of combined (male + female) peak body weight in SMA + SR12519 (4.98 ± 0.20, *n* = 8) with SMA + DMSO (3.36 ± 0.12) (*P* = 1.05 × 10^−5^) shows an average peak body weight increase of ∼48% in SMA + SR12519 mice. (**G–I**) JNK inhibition improves postnatal growth period (days) (mean ± SEM, *n* = 3) of SMA mice. Growth period of (**G**) SMA + SR12519 (11.00 ± 0.57) males compared with SMA + DMSO (6.0 ± 0.57) male mice shows ∼83% increase (*P* = 3.60 × 10^−3^) in postnatal growth period, (**H**) SMA + SR12519 females (12.33 ± 0.33) compared with SMA + DMSO (7.66 ± 0.88) female mice shows ∼61% increase (*P* = 7.76 × 10^−3^) in the postnatal growth period, (**I**) Comparison of combined (males + females), SMA + SR12519 (11.67 ± 0.42) to SMA + DMSO (6.83 ± 0.60) males + females shows a statistically significant (*P* = 6.20 × 10^−5^) increase in postnatal growth period. (**J–R**) Pharmacological inhibition of JNK improves survival of SMA mice. (**J–L**) Kaplan–Meier survival curves of normal (blue), SMA + DMSO (orange) and SMA + SR12519 (magenta) mice littermates. Vertical dotted lines show median survival. Survival analysis shows 2.0-fold increase in median survival of SMA mice treated with drug SR12519 compared with control SMA + DMSO mice (Log-rank test, *P* < 1.0 × 10^−10^). (**M–O**) The scatter plots show average survival (days) (mean ± SEM) of SMA mice untreated (SMA), treated with DMSO (SMA + DMSO) and JNK inhibitor SP600125 (SMA + SR12519). (**M**) Sex-based analysis shows that the average survival of SMA + SR12519 males (16.0 ± 0.25, *n* = 10, *P* = 2.69 × 10^−5^) is higher than SMA + DMSO (9.6 ± 1.18, *n* = 10) males. (**N**) Average survival of SMA + SR12519 females (15.70 ± 0.26, *n* = 10) is also higher compared with SMA + DMSO (7.70 ± 1.19, *n* = 10, *P* = 3.71 × 10^−6^) females. (**O**) Statistical analysis of male and female survival show increase in average survival (mean ± SEM) days of SMA + SR12519 (15.80 ± 0.18, *n* = 20) (unpaired *t*-test, *P* = 3.0 × 10^−10^) compared with SMA + DMSO (8.65 ± 0.82, *n* = 20) mice. (**P–R**) Scatter plots show a marked increase (5-fold) in initial (minimum) survival of SMA mice treated with JNK inhibitor compared with SMA + DMSO mice. All males and females (*n* = 20) treated with AS601245 survived at least 15 days compared with SMA males and females that survived at least 3 days. Survival and growth analyses were blinded with colour coding. Sex and genotypes were confirmed by PCR-based method after euthanasia of pups.

The sex-based analysis of overall growth of mice normal (blue), SMA + DMSO (orange) and SMA + SR12519 (magenta) shows statistically significant increase in the body weight of male (*P* = 1.30 × 10^−7^), female (*P* = 8.59 × 10^−7^), and male + female (*P* = 3.15 × 10^−7^) SMA mice treated with SR12519 compared DMSO treated mice (Fig. 7A–C). Analysis of average peak body weight in males [SMA + SR12519 (5.28 ± 0.32, *P* = 2.71 × 10^−3^) versus SMA + DMSO (3.54 ± 0.15)] and in females [SMA + SR12519 (4.67 ± 0.17, *P* = 9.97 × 10^−4^) versus SMA + DMSO (3.19 ± 0.18)] and combined (male + female) [SMA + SR12519 (4.97 ± 0.20, *P* = 1.05 × 10^−5^) versus SMA + DMSO (3.36 ± 0.12)] mice also shows statistically significant improvement in the growth of SMA mice treated with SR12519 (Fig. 7D–F). These data show that overall growth increase in SMA + SR12519 males is ∼49% (*P* = 2.71 × 10^−3^) and in females is ∼46% (*P* = 9.97 × 10^−4^) compared with DMSO treated SMA males and females (Fig. 7D and E). JNK inhibition also increased the growth period of male mice from (6.000 ± 0.57) (SMA + DMSO) to (11.00 ± 0.57, *P* = 3.60 × 10^−3^) (SMA + SR12519) days and female mice from (7.66 ± 0.88) (SMA + DMSO) to (12.33 ± 0.33, *P* = 7.76 × 10^−3^) (SMA + SR12519) days that shows ∼83% increase in males and ∼61% increase in females in the postnatal growth periods, respectively (Fig. 7G–I). Together, these data suggest that *in vivo* pharmacological JNK3 inhibition improves overall growth and postnatal growth period of SMA mice treated with high potency JNK3 inhibitor SR12519, which may also help reduce severity of disease and increase the lifespan of SMA mice.

### JNK3 inhibitor SR12519 improves the lifespan of SMA mice

The comparison of median survival of control mice (SMA + DMSO) (8 days) and JNK inhibitor SR12519 (SMA + SR12519) shows ∼2.0-fold increase in combined (male + female) median survival (16 days) of SMA + SR12519 mice (Log-rank test, *P* < 1.0 × 10^−10^). The sex-based comparison of median survival of males (∼1.78-fold, *P* < 1.0 × 10^−10^) and females (∼2.07-fold, *P* < 1.0 × 10^−10^) also shows significant increase in the lifespan of SMA + SR12519 compared with control SMA + DMSO mice (Fig. 7J and K). Analysis of average survival of SMA + SR12519 males (16.00 ± 0.25, *P* = 2.69 × 10^−5^) and median survival (16.0 days) are higher than SMA + DMSO males average (9.60 ± 1.11) and median (9.0 days) survival (Fig. 7J and M). Average survival of SMA + SR12519 females (15.70 ± 0.26, *P* = 3.71 × 10^−5^) and median survival (15.5 days) are also higher than SMA + DMSO females average (7.70 ± 1.19) and median (7.5 days) survival (Fig. 7K and N). The combined (male + female) average survival of SMA + SR12519 mice (mean ± SEM) (15.80 ± 0.18, *P* = 3.0 × 10^−10^) days compared with SMA + DMSO (8.65 ± 0.82) (Fig. 7L and O) shows statistically highly significant increase in survival of SMA mice with SR12519. Comparison of average lifespan among SMA + SR12519 males (∼1.78-fold) and females (∼2.06-fold) shows combined JNK1 and JNK3 inhibition results in slightly better improvement in the lifespan of females compared with male SMA mice (Fig. 7M–O). However, the increase in the overall growth of females ∼46% is slightly lower than the males ∼49% mice treated with JNK inhibitor SR12519 (Fig. 7D–F). The 3 days-increase in maximum survival of SMA + SR12519 mice (∼22% increase in max lifespan) includes 5 days of growth (reduced severity), resulting in about a 1.7-fold increase in the lifespan with reduced severity of the disease (Fig. 7J–L). Analysis of minimum survival shows marked increase (5.0-fold, *P* = 2.90 × 10^−9^) in the initial survival of SMA + SR12519 mice compared with control SMA mice. These data show that all mice, male or female (*n* = 20), treated with SR12519, survived for at least 15 days after birth compared with SMA + DMSO (control) that started dying at the age of 3 days (Fig. 7P–R). These data suggest that combined inhibition of JNK1 and JNK3 SR12519 reduced postnatal disease severity, moderately increased the lifespan and markedly increased minimum survival of SMA mice.

### Inhibition with SR12519 improves gross motor function and muscle strength of SMA mice

**Figure 8:**
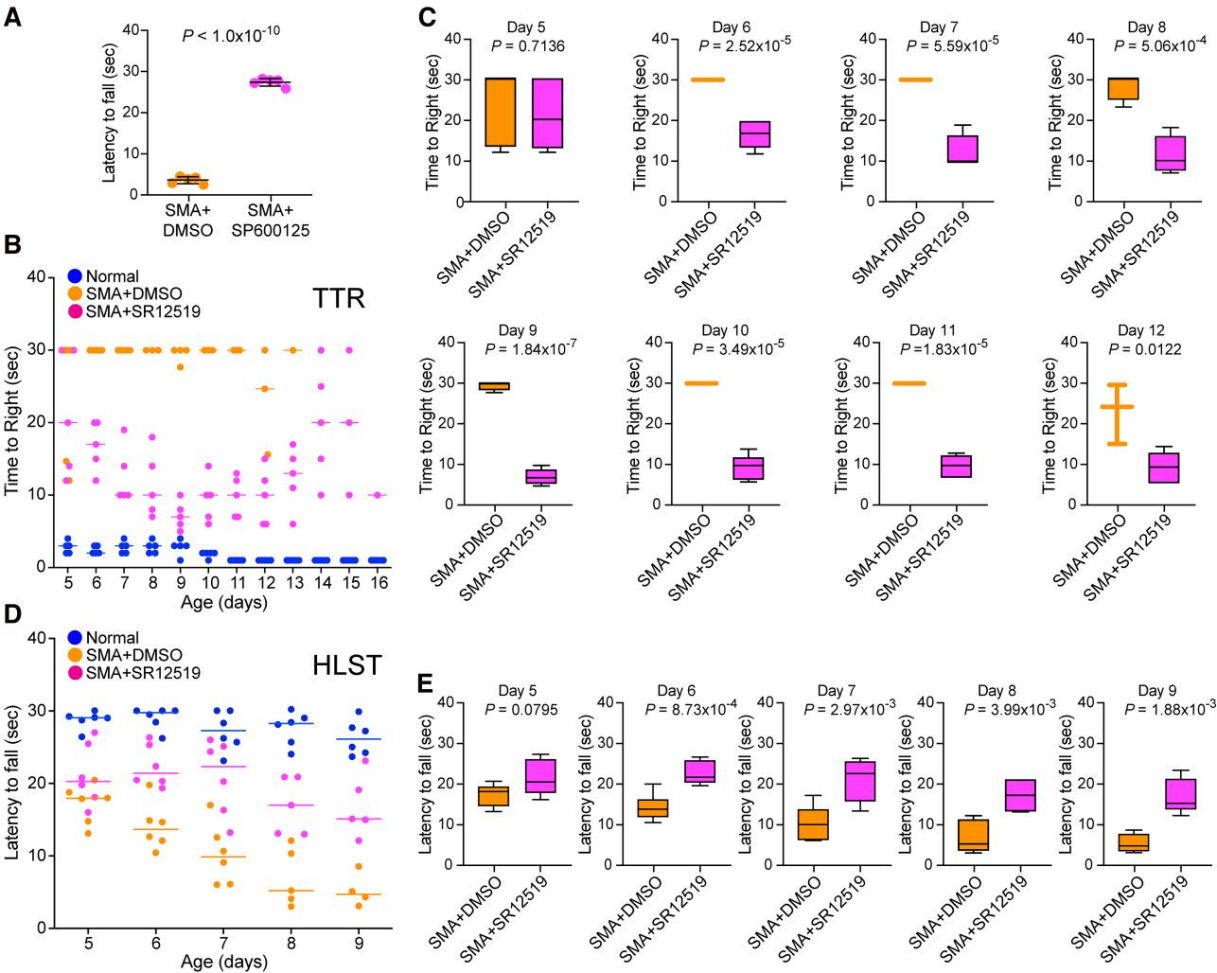
**Pharmacological JNK inhibition with SR12519 improves the gross motor function and muscle strength of SMA mice.** (**A**) JNK inhibition improves ability to stand on paws and walk. Improvement in the ability of SMA mice to stand on paws and walk was determined by measuring the time of falling after walking on four paws and was recorded in 10-day-old SMA + DMSO and SMA + SR12519 littermates. Comparison of latency to fall (seconds) from paws is presented as a scatter plot with individual data points that represent an average of three recordings per pup/group/day showing all pups treated with the drug compound were able to stand and walk for an average time of ∼27 s suggesting an improvement in the gross motor function. Statistical analysis of (mean ± SEM, *n* = 5 mice/group, male + female) (seconds) between SMA + DMSO (3.4 ± 0.38) and SMA + SR12519 (27.20 ± 0.41), *P* < 1.0 × 10^−10^) (unpaired *t*-test, two-tailed) for 10-day-old mice shows marked improvement in the ability of SMA mice treated with JNK inhibitor to stand on paws and walk (see Supplementary Videos 7–9). (**B**) Mice righting reflexes were examined for 5–16-day-old normal (blue), SMA + DMSO (orange), and SMA + SR12519 (magenta) littermates. TTR with a time limit of 30 s for the test and an average of 3 recordings per pup/day/group are plotted. Data were collected from three groups starting with *n* = 5 mice/group, which are presented as a scatter plot. JNK inhibitor treated mice show improvement in their ability to right until PND18. (**C**) Improvements in the motor function (righting) is demonstrated by an age-dependent increase in the ability of mice to right upon treatment with JNK inhibitor SMA + SR12519 compared with control SMA mice treated with DMSO (SMA + DMSO). The results are shown as box-and-whisker plots (median, min, max) starting from PND5 [SMA + DMSO (30.0, 12.0, 30.0) and SMA + SR12519 (20, 12.0, 30.0)], to PND12 [SMA + DMSO (24.67, 15.60, 30.0) and SMA + SR12519 (10.0, 6.0, 15.0)]. Statistical analysis (mean ± SEM) shows slight improvement in TTR at PND5 [SMA + SR12519 (21.20 ± 3.8, *n* = 5)] compared with control [SMA + DMSO (23.33 ± 4.10)] but it is not statistically significant (unpaired *t*-test, *P* = 0.7136). Analysis of righting ability from PND6 to PND12 shows statistically significant improvement in TTR of JNK inhibitor treated mice; PND6 [SMA + SR12519 (16.80 ± 1.53, *n* = 5), SMA + DMSO (30.0 ± 0.0, *n* = 5) unpaired *t*-test, *P* = 2.52 × 10^−5^], PND7 (12.60 ± 1.77), *P* = 5.59 × 10^−5^], PND8 (11.40 ± 2.04), *P* = 5.06 × 10^−4^, PND9 (7.20 ± 0.86), *P* = 1.84 × 10^−7^, PND10 (9.40 ± 1.40), *P* = 3.49 × 10^−5^, PND11 (9.80 ± 1.24), *P* = 1.83 × 10^−5^ and PND12 (9.80 ± 1.74), *P* = 0.0122 compared with SMA + DMSO (30.0 ± 0.0) from PND7-12. (**D**) The HLST shows that JNK inhibition increases muscle strength in mice with SMA. Littermates of 5–9 days of age were hung on both hind legs on the edge of a 50 ml plastic conical tube and time (seconds) was recorded until fall from the edge of the tube. Latency to fall (mean ± SEM, 6 mice/group, male + female) is shown as a scatter plot and (**E**) as box-and-whisker plots with median with interquartile range (median, min, max) for each time point (day), PND5 [SMA + DMSO (17.93, 13.09, 20.43) and SMA + SR12519 (20.26, 15.98, 26.98)], PND6 [SMA + DMSO (13.65, 10.43, 19.76) and SMA + SR12519 (21.40, 19.34, 26.30)], PND7 [SMA + DMSO (9.87, 6.02, 16.98) and SMA + SR12519 (22.30, 13.20, 25.98)], PND8 [SMA + DMSO (5.21, 3.02, 12.09) and SMA + SR12519 (16.98, 12.98, 20.87)], and PND9 [SMA + DMSO (4.70, 3.09, 8.54) and SMA + SR12519 (15.09, 12.09, 23.09)]. Statistical analysis (*t*-test, unpaired) shows marked and statistically significant increase in hanging time (seconds) for JNK inhibitor treated compared with DMSO treated SMA mice with increasing age; PND5 [SMA + SR12519 (21.17 ± 1.73) and SMA + DMSO (17.15 ± 1.10), *P* = 0.0795], PND6 [SMA + SR12519 (22.34 ± 1.17) and SMA + DMSO (14.08 ± 1.32), *P* = 8.73 × 10^−4^], PND7 [SMA + SR12519 (20.86 ± 2.13) and SMA + DMSO (10.23 ± 1.70), *P* = 2.97 × 10^−3^], PND8 [SMA + SR12519 (16.96 ± 1.75) and SMA + DMSO (6.94 ± 1.79), *P* = 3.99 × 10^−3^], and PND9 [SMA + SR12519 (16.87 ± 1.91) and SMA + DMSO (5.26 ± 1.16), *P* = 1.88 × 10^−3^] shows gradual increase in the muscle strength of JNK inhibitor treated mice compared with control SMA mice.

The ability to stand on four paws and walk was improved in 10-day-old SMA + SR12519 mice (27.20 ± 0.41, *P* < 1.0 × 10^−10^) compared with control (SMA + DMSO) mice (2.80 ± 0.17) (Fig. 8A). Video clips of 8-, 10-, and 13-day-old littermates treated with DMSO and SR12519 (Supplementary Videos 7–9) show that SMA + SR12519 mice were able to walk and were able to right themselves within a few seconds and continued to walk compared with control SMA + DMSO mice that were unable to right themselves and walk. These data show combined JNK1 and JNK3 inhibition results in marked improvements in the gross motor function of SMA mice.

Analysis of –16-day-old mice TTR did not show statistically significant improvement at PND5 between SMA + DMSO (23.33 ± 4.104, *n* = 6) and SMA + SR12519 (21.20 ± 3.826, *n* = 6), *P* = 0.7136 (Fig. 8B and C). However, comparison of TTR from PND6 to PND12 shows improvement in the gross motor function of SMA + SR12519 compared with SMA + DMSO mice PND6 [SMA (30.00 ± 0.00) and SMA + SR12519 (16.80 ± 1.53, *P* = 2.52 × 10^−5^] to PND12 [SMA (30.00 ± 0.00) and SMA + SR12519 (9.80 ± 1.74), *P* = 0.0122], which is statistically significant (Fig. 8B and C). All SMA pups were dead by the Day 14, however, JNK inhibitor treated mice continued to right until PND16 (Fig. 8C). Increase in the ability to right with reduction in TTR of JNK inhibitor-treated mice compared with control SMA mice with increasing age suggests gradual improvement with aging in the gross motor function and notable reduction in the disease severity of SMA + SR12519 mice with a single dose (10 mg/kg) of treatment on alternate days with this drug compound.

Representative video clips of 8-, 10-, 13-day-old SMA + DMSO and JNK inhibitor treated littermates show that SMA + SR12519 mice were able to right and walk and remained standing on four paws after walk (Supplementary Videos 7–9). Improvement in multiple motor functions such as righting, standing on four paws and walking in the late stages of lifespan likely due to increase in muscular strength with JNK inhibition. The analysis of HLST data at PND5 [SMA + SR12519 (21.17 ± 1.73, *n* = 6) versus SMA + DMSO (17.17 ± 1.10, *n* = 6)] did not show statistically significant difference (*P* = 0.0795) (Fig. 8D and E). However, analysis of SMA littermates from PND6 to PND9 showed marked and statistically significant increase in muscle strength of SMA mice treated with SR12519 compared with SMA + DMSO mice at PND6 [SMA + DMSO (14.08 ± 1.32) and SMA + SR12519 (22.34 ± 1.17, *P* = 0.0009)], PND7 [SMA + DMSO (10.23 ± 1.70) and SMA + SR12519 (20.86 ± 2.13, *P* = 0.0030)]. The hanging time at PND8 [SMA + DMSO (6.94 ± 1.79) and SMA + SR12519 (16.96 ± 1.75, *P* = 0.0040)] and PND9 [SMA + DMSO (5.26 ± 1.16) and SMA + SR12519 (16.87 ± 1.91, *P* = 0.0019)] for JNK inhibitor treated mice reduced but showed statistically significant increase in muscle strength (Fig. 8D and E). Increase in hind-limb hanging time suggests improvement in hind-limb muscle strength of JNK inhibitor treated mice compared with control SMA mice. Together, these data suggest that potent and selective inhibitor of JNK3 and JNK1 improved gross motor function and muscle strength of SMA mice. Notably, amelioration of SMA disease severity with pharmacological JNK inhibition using compound SR12519 with high potency for JNK3 is consistent with the findings of genetic inhibition of JNK3 in SMA mice.^26^

### Analysis of outcomes of the combination of data from three JNK inhibitors

To gain insight into overall beneficial impact of JNK inhibition on amelioration of disease severity in SMA mice, we combined data from three JNK inhibitors (SP600125, AS601245, SR12519) and analysed increase in the overall growth, survival and motor functions of SMA mice treated with JNK inhibitors compared with control SMA mice. The data from three independent experiments with inhibitors were pooled without any trimming or exclusion of data points. The comparison of overall growth of mice treated with individual inhibitors and combined data show highly statistically significant improvement in the growth of males (*P* = 1.73 × 10^−7^), females (*P* = 2.38 × 10^−7^), and male + females (*P* = 2.11 × 10^−7^) (Supplementary Fig. 4). The Kaplan–Meier analysis of mice treated with JNK inhibitors shows >2-fold increase in the survival with very high statistical significance for males (*P* < 1.0 × 10^−10^), females (*P* < 1.0 × 10^−10^) and male + females (*P* < 1.0 × 10^−10^) (Supplementary Fig. 5). Tables for comparison of statistical significance of outcomes of individual and all inhibitors are included in Supplementary Figs 4 and 5. The statistical analysis (ANOVA) of combined data of TTR also shows remarkable improvement in the righting reflexes of mice treated with JNK inhibitors with aging (5–14 days) and statistical significance ranges from *P* = 4.11 × 10^−7^ to *P* < 1.0 × 10^−10^ (Supplementary Fig. 6). Notably, SMA mice treated with JNK inhibitors continued to right up to 18 days of age. Analysis of muscle strength (HLST) also shows marked increase in hindlimb strength of 5–9-day-old SMA mice treated with JNK inhibitors compared with control SMA mice with muscle strength improving steadily during the period of 7–9 days of age with statistical significance (ANOVA) ranging from *P* = 3.68 × 10^−7^ to *P* < 1.0 × 10^−10^ (Supplementary Fig. 7). Together, the analysis of individual and combined data from JNK inhibitors suggests that pharmacological JNK inhibition shows statistically significant improvements in overall growth, gross motor functions and muscle strength, and increase (∼2.0-fold) in the lifespan that contribute to the amelioration of disease severity in SMA mice.

Analysis of overall data from *in vivo* testing of three JNK inhibitors with unique chemical scaffolds shows amelioration of SMA disease severity by improvement in overall growth, motor function and muscle strength and increase in the lifespan of SMA mice. Detailed comparison of similarities and differences in the effects of these three drug compounds and sex-based outcomes are discussed in the ‘Discussion’ section and presented in a tabular form in Supplementary Table 3. Importantly, the finding that *in vivo* inhibition of JNK with three drug compounds with different chemical structures resulted in similar improvements and amelioration of the SMA disease suggest that pharmacological JNK inhibition is a viable option for the treatment of SMA.

### Effect of JNK inhibition on SMN levels in SMA mice

**Figure 9:**
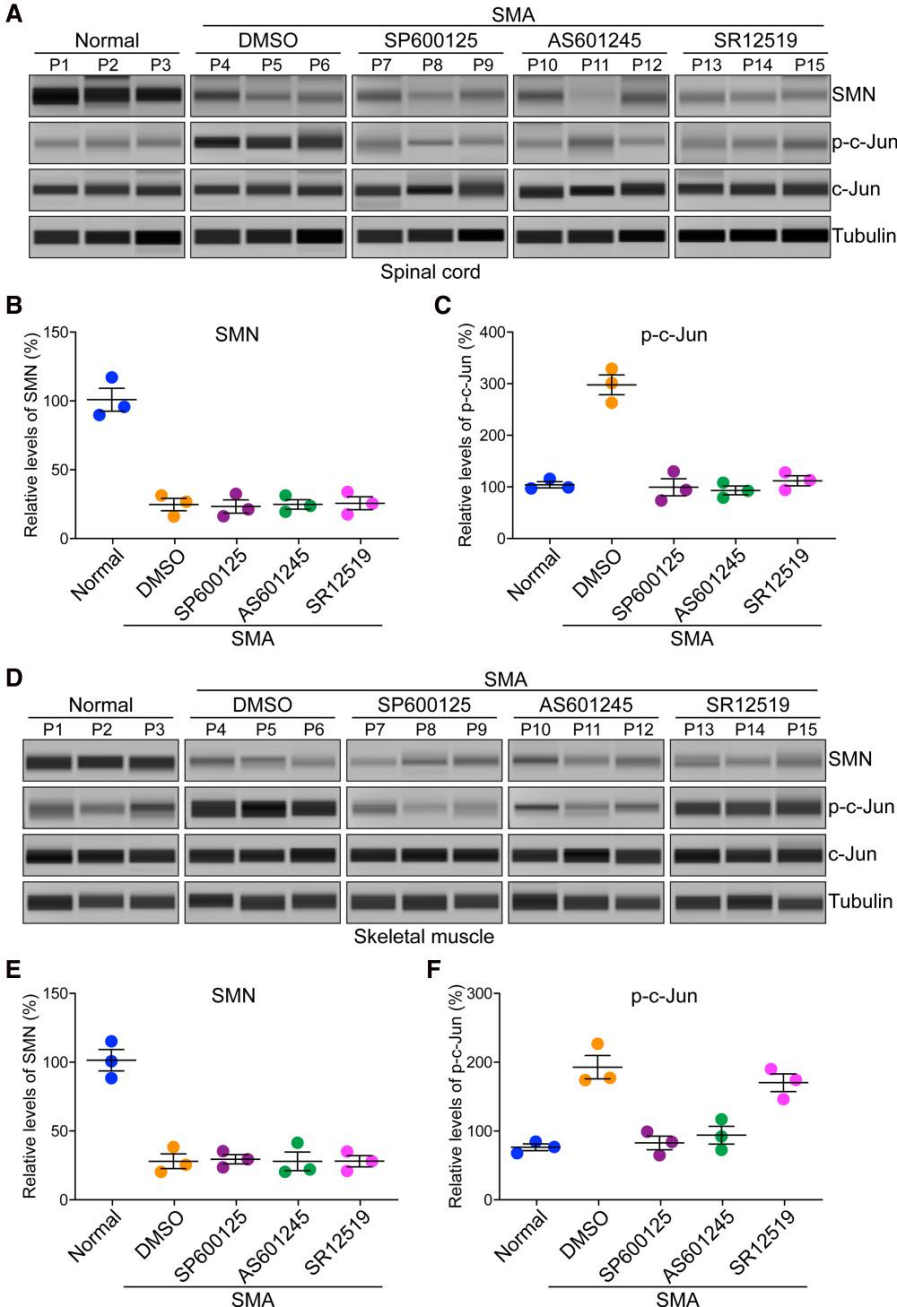
**Effect of pharmacological JNK inhibition with drug compounds on the levels of p-c-Jun and SMN proteins in SMA mice.** Proteins levels of SMN, p-c-Jun, SMN, total c-Jun (c-Jun) and tubulin were examined by IB analysis of mouse tissues, spinal cord, and skeletal muscle from 10-day-old normal, SMA mice treated with DMSO and with JNK inhibitors SP600125, AS601245, and SR12519 using automated capillary western blot system (ProteinSimple). (**A**) Representative capillary-IB images of SMN, p-c-Jun, c-Jun and tubulin proteins from the spinal cord tissues of mice are shown (uncropped blots are included in Supplementary Figs 8 and 9). Each group of mice, normal and SMA mice treated with DMSO or JNK inhibitors show individual pups numbered from P1-15 with *n* = 3 mice/group. Quantitative analysis was performed by normalization against tubulin as a loading control and using Compass software. Quantitative data are shown as scatter plots, with individual points representing the average densitometric measurement of each blot for various protein markers (SMN, p-c-Jun, total c-Jun, and tubulin). Each data point corresponds to a single mouse within the group (3 mice/group). The plots illustrate the relative change in protein levels (%) normalized to tubulin *in vivo* in the spinal cords following treatment with JNK inhibitors: (**B**) Statistical analysis (ANOVA) of protein levels (mean ± SEM, *n* = 3 mice/group) of four groups SMA + DMSO, SMA + SP600125, SMA + AS601245, and SMA + SR12519 did not show significant change (*P* = 0.9913) in SMN levels upon treatment with JNK inhibitors. The unpaired *t-*test with Welch’s correction analysis shows that *in vivo* treatment with JNK inhibitors did not cause statistically significant change in SMN levels of spinal cords from SMA + SP600125 (22.50 ± 4.80, *P* = 0.8368), SMA + AS601245 (24.02 ± 3.47, *P* = 0.9913) and SMA + SR12519 (24.84 ± 4.70, *P* = 0.8985) compared with SMA + DMSO (23.95 ± 4.51) mice. (**C**) Statistical analysis (ANOVA) of p-c-Jun levels (mean ± SEM, *n* = 3 mice/group) of three groups (i) normal, SMA + DMSO, SMA + SP600125 (1.24 × 10^−4^), (ii) normal, SMA + DMSO, SMA + AS601245 (4.13 × 10^−5^), and (iii) normal, SMA + DMSO, SMA + SR12519 (6.38 × 10^−5^) shows significant change in p-c-Jun levels upon treatment with each drug compound and indicates marked *in vivo* JNK inhibition in the spinal cords. The unpaired *t*-test with Welch’s correction of protein levels (mean ± SEM, *n* = 3 mice/group) shows that *in vivo* treatment with JNK inhibitors caused statistically significant decrease in the p-c-Jun levels from the spinal cord of SMA + SP600125 (95.74 ± 16.45, *P* = 0.0016), SMA + AS601245 (89.65 ± 8.39, *P* = 0.0033), and SMA + SR12519 (108.20 ± 9.83, *P* = 0.0034) compared with SMA + DMSO (294.20 ± 19.21). Comparison of p-c-Jun levels in the spinal cords of inhibitor treated mice with normal mice (100.50 ± 5.92) shows JNK inhibition effectively decreased levels of p-c-Jun in SMA mice. (**D**) IB images of SMN, p-c-Jun, c-Jun and tubulin proteins from the skeletal tissues from hind limbs of mice are shown (uncropped blots are included in Supplementary Figs 10 and 11). Quantitative data are shown as scatter plots with individual points showing average densitometric measurement of blots representing relative change in protein levels (%) normalized to tubulin *in vivo* in skeletal muscle after treatment with JNK inhibitors: (**E**) Statistical analysis (ANOVA) of protein levels (mean ± SEM, *n* = 3 mice/group) of four groups SMA + DMSO, SMA + SP600125, SMA + AS601245, and SMA + SR12519 did not show significant change (*P* = 0.9956) in SMN levels upon treatment with JNK inhibitors. The unpaired *t*-test with Welch’s correction of protein shows that *in vivo* treatment with JNK inhibitors did not cause statistically significant change in SMN levels of skeletal muscle from SMA + SP600125 (28.68 ± 3.36, *P* = 0.8325), SMA + AS601245 (27.18 ± 6.75, *P* = 0.9951), and SMA + SR12519 (27.28 ± 4.08, *P* = 0.9952) compared with SMA + DMSO (27.24 ± 5.34) mice. (**F**) Statistical analysis (ANOVA) of p-c-Jun levels (mean ± SEM, *n* = 3 mice/group) of three groups (i) normal, SMA + DMSO, SMA + SP600125 (6.80 × 10^−4^), (ii) normal, SMA + DMSO, SMA + AS601245 (1.29 × 10^−3^), and (iii) normal, SMA + DMSO, SMA + SR12519 (6.80 × 10^−4^) shows significant change in p-c-Jun levels upon treatment with each drug compound and indicates marked *in vivo* JNK inhibition in the skeletal muscle. The unpaired *t-*test with Welch’s correction of protein levels shows that *in vivo* treatment with JNK inhibitors caused statistically significant decrease in the p-c-Jun levels from the skeletal muscle of SMA + SP600125 (108.40 ± 13.24, *P* = 0.0094) and SMA + AS601245 (123.40 ± 17.21, *P* = 0.0117) but not for SMA + SR12519 (225.0 ± 17.01, *P* = 0.3529) compared with SMA + DMSO (255.10 ± 22.73). Comparison of p-c-Jun levels in the skeletal muscle of inhibitor treated mice with normal mice (100.10 ± 6.45) shows JNK inhibition with SP600125 and AS601245 effectively decreased levels of p-c-Jun in SMA mice. Inhibition with SR12519, high potency for JNK3, did not decrease p-c-Jun levels significantly suggesting the lack of JNK3 in skeletal muscle and demonstrating the inhibition specificity of SR12519 towards JNK3 but not JNK1 and JNK2.

To test whether JNK inhibition-dependent rescue of SMA disease is SMN-dependent or independent, we examined the effect of drug compounds on the levels of SMN, using JNK inhibition in the spinal cords of littermates treated with DMSO or JNK inhibitors. The spinal cords were harvested from 10-day-old mice and total proteins extracts were examined by automated quantitative IB analysis. IBs of spinal cord tissues for SMN, p-c-Jun, total c-Jun (c-Jun), and alpha-tubulin (tubulin) from three mice per group are shown in Fig. 9A (uncropped blots are included in Supplementary Figs 8 and 9). Quantitative and statistical analyses (mean ± SEM, *n* = 3)% show that SMA mice treated with DMSO show low SMN levels (23.95 ± 4.51) compared with normal (100.0 ± 8.28) mice (Fig. 9A and B), conforming the cause of the SMA disease and consistent with previous findings.^16,26,27^ Interestingly, comparison of SMN levels in control SMA mice treated with DMSO (23.95 ± 4.51) and mice treated with JNK inhibitors SP600125 (22.50 ± 4.80, *P* = 0.8368), AS601245 (24.02 ± 3.47, *P* = 0.9913), and SR12519 (24.84 ± 4.70, *P* = 0.8985) did not show statistically significant differences suggesting that treatment with all three JNK inhibitor drug compounds did not influence *in vivo* SMN levels in the spinal cords of SMA mice. Analysis of JNK activation/inhibition by examining levels of p-c-Jun, the most studied downstream target of activated JNK,^82^ show that JNK is activated (∼3.0-fold) in the spinal cords of SMA mice treated with DMSO (294.2 ± 19.21) compared with normal (100.5 ± 5.92) mice (Fig. 9A and C). Notably, comparison of p-c-Jun levels in the spinal cords of SMA mice treated with JNK inhibitors SP600125 (95.74 ± 16.45, *P* = 0.0016), AS601245 (89.65 ± 8.39, *P* = 0.0033), and SR12519 (108.2 ± 9.83, *P* = 0.0034) with SMA + DMSO (294.2 ± 19.21) shows efficient inhibition of JNK resulting in statistically significant reduction in p-c-Jun levels that are close to p-c-Jun levels (100.5 ± 5.92) in normal mice (Fig. 9A and C). IBs of total c-Jun (c-Jun) show that JNK inhibitors did not change the levels of total c-Jun protein suggesting that the drug compounds did not alter the levels of c-Jun and demonstrate the specificity of these compounds for inhibiting enzyme activity of JNK towards c-Jun phosphorylation. Together, these data suggest that JNK inhibitors selected based on *in vitro* analysis of JNK inhibition in SMA motor neurons are equally potent in inhibiting JNK *in vivo* without altering the levels of the SMN protein in the spinal cords of SMA mice.

Skeletal muscle is another important tissue affected by SMA pathogenesis. Improvement in the gross motor function, ability to right, stand and walk of SMA mice treated with JNK inhibitors suggest amelioration of muscle weakness, which is also supported by the data from HLST test of SMA mice treated with JNK inhibitors (Figs 4, 6, and 8). Interestingly, JNK signalling is shown to play critical role in skeletal muscle remodelling and muscle wasting.^83-87^ However, the role of JNK in skeletal muscle atrophy of SMA is unknown. To gain insight into the contribution of JNK signalling in muscle atrophy associated with SMA pathogenesis, we examined JNK activation in skeletal muscle in hind limb skeletal muscle tissues from SMA mice and compared the effect of three JNK inhibitors on levels of SMN and p-c-Jun. IBs of skeletal muscle tissues for SMN, p-c-Jun, c-Jun and tubulin from three mice per group are shown in Fig. 9D (uncropped blots are included in Supplementary Figs 10 and 11). IBs quantitative and statistical analyses (mean ± SEM, *n* = 3)% of p-c-Jun levels show that the JNK is activated (∼2.5-fold) in the skeletal muscle of SMA mice (DMSO) (255.1 ± 22.73) compared with normal mice (100.1 ± 6.45) (Fig. 9D and F). Treatment with two pan-JNK inhibitors SP600125 and AS601245 resulted in the inhibition of JNK in skeletal muscle and caused significant decrease in p-c-Jun levels in SMA + SP600125 (108.4 ± 13.24%, *P* = 0.0094) and SMA + AS601245 (123.4 ± 17.21, *P* = 0.0117) that is close to the levels of p-c-Jun in normal mice (100.1 ± 6.45) (Fig. 9D and F). In contrast, treatment with JNK3-specific inhibitor did not reduce p-c-Jun levels (225.0 ± 17.01, *P* = 0.3529) significantly in SMA + SR12519 compared with control SMA mice treated with DMSO (255.1 ± 22.73) suggesting that JNK3 is not activated in skeletal muscle and is consistent with findings that the JNK3 isoform is mainly expressed in the CNS.^26,88,89^ Comparison of data from the spinal cord and skeletal muscle tissues of SMA mice treated with the SR12519 compound shows JNK inhibition in spinal cords but not in skeletal muscle, demonstrating the specificity and selectivity of SR12519 towards JNK3 isoform in the spinal cords (Fig. 8A and D). Quantitation and statistical analysis of SMN levels in skeletal muscle also show low SMN in SMA + DMSO mice (27.24 ± 5.34) compared with normal (100.6 ± 7.73) mice (Fig. 9D and E) and is consistent with data from the spinal cords (Fig. 9A and B). Notably, comparison of skeletal muscle SMN levels in control SMA mice (27.24 ± 5.34) treated with DMSO and mice treated with JNK inhibitors SP600125 (28.68 ± 3.36, *P* = 0.8325), AS601245 (27.18 ± 6.75, *P* = 0.9951), and SR12519 (27.28 ± 4.08, *P* = 0.9952) did not show statistically significant differences suggesting that JNK inhibition did not alter SMN levels in the skeletal muscle of SMA mice (Fig. 9D and E). Together, IB analysis of the spinal cord and skeletal muscle tissues shows that selected three JNK inhibitors can penetrate CNS and muscle tissue and effectively inhibit JNK without altering SMN levels in the target tissues that are primarily affected in SMA.

Overall, these data show that pharmacological inhibition of JNK resulted in the amelioration of disease severity by improving body growth, gross motor function (righting reflexes and ability to walk), muscle strength, and increased the lifespan of SMA mice without changing SMN levels in the spinal cords and skeletal muscle tissues of SMA mice. These findings suggest that amelioration of disease severity and partial rescue of disease phenotype in a severe SMA mouse model by JNK inhibition is SMN-independent.

## Discussion

SMA is a severe hereditary disorder of early childhood characterized by motor neuron degeneration, progressive muscle weakness and atrophy. The SMA disease has a distinctive genetic architecture that has not only expanded our understanding of its pathogenesis but also accelerated the development of targeted therapies. A particularly remarkable feature of SMA genetics is the presence of the *SMN2* gene, an invertedly duplicated homolog of the *SMN1* gene, which differs by a few critical point mutations. This unique genomic configuration has opened promising avenues for therapeutic intervention and even the potential for a cure. The tremendous amount of research done in the past two decades identified *SMN2* as most promising modifier gene to treat and cure SMA in an SMN-dependent manner and used to successfully develop currently available two splicing correction-based FDA approved treatments, Spinraza (nusinersen) and Evrysdi (risdiplam).^6^ However, recent research has also uncovered several SMN-independent protective modifier genes, including, *PLS3*, *NCALD*, *CHP1*, *CORO1C*, *STMN1*, *DOK7*, *Z*  *+*  *Agrin*, *JNK3*, and *SETX*. In addition, various molecular targets and biochemical pathways have been identified that show promising potential for the development of SMN-independent therapeutic strategies. Notably, data from clinical trials and post-treatment analyses indicating only marginal improvements in some SMA patients have led to a growing consensus within the scientific community on the need to develop more effective therapeutic approaches. This includes the pursuit of SMN-independent strategies, which could be employed either as standalone therapies or in combination with SMN-dependent treatments to address the needs of patients with mild, moderate and severe forms of SMA more effectively.^90-95^

### JNK inhibition prevents degeneration of motor neurons in SMA

The scientific premise of this study builds upon previous findings that demonstrated a role for the JNK signalling pathway in mediating neurodegeneration in SMA. These studies led to the identification of JNK3, a neuron-specific isoform,^88^ as a critical factor in preventing the degeneration of SMN-deficient neurons in SMA.^26^ Furthermore, genetic inhibition of JNK3 *in vivo* by ablation of the *Jnk3* gene resulted in systemic rescue of the SMA phenotype in mice.^26^ The genetic inhibition of JNK3 resulted in amelioration of disease severity demonstrated by statistically significant improvements in the overall growth, gross motor functions, muscle fibre size, muscle growth, muscle strength and NMJ innervation.^26^ These systemic improvements were due to prevention of motor neuron degeneration, which reduced muscle atrophy and contributed to increase in the average survival (>2.0-fold) and increase in the initial or minimum survival (4.20-fold) of SMA mice lacking the *Jnk3* gene.^26^ JNK inhibition using the JNK peptide inhibitor, D-JNKI-1 showed slight but significant increase in the survival of SMA mice.^30^ These findings provided a rationale for the investigation of pre-clinical aspects of pharmacological JNK inhibition on the rescue of the SMA disease and generate a proof-of-concept that JNK inhibition may represent a potent option for developing SMN-independent strategies to prevent neurodegeneration and reduce disease severity. The limited success with developing effective therapeutic treatments for neurological disorders is due to the lack of testing pharmacological agents on relevant neuronal cell types that are targets of disease pathogenesis. To overcome this challenge, we performed a rigorous *in vitro* testing of JNK inhibitors using primary cultured neurons, including spinal cord motor neurons that primarily degenerate in SMA. The *in vitro* analysis, using cultured primary spinal cord motor neurons derived from SMA mice, provided insight and allowed the selection of non-toxic and most potent pan- and JNK3-specific inhibitors for *in vivo* sex-based analysis using mice with a severe form of SMA disease. Prevention of *in vitro* degeneration of SMN-deficient CGN and spinal motor neurons derived from SMA mice using three drug compounds with distinct chemical scaffolds, SP600125 (Anthrapyrazolone), AS601245 (Pyrimidinyl), SR12519 (Pyridopyrimidine) and diverse potency, shows that JNK inhibition via binding to an ATP-binding site, a common feature among the drugs, is responsible for preventing neurodegeneration and not the chemical composition or 3D-conformation of the drug compounds. These findings suggest that inhibition of JNK signalling is a critical requirement for preventing the degeneration of SMN-deficient motor neurons in SMA.

### Pharmacological JNK inhibition ameliorates disease severity in severe SMA mice

To contribute to the unmet need of discovering and developing SMN-independent methods for the treatment of SMA,^90^ we set out to test the hypothesis that pharmacological JNK inhibition represents an option for preventing neurodegeneration and for ameliorating SMA disease severity. We investigated the impact of *in vivo* JNK inhibition using three drug compounds and SMAΔ7 mouse model with severe form of disease. Our *in vivo* data show that JNK inhibition with all three drug compounds resulted in the amelioration of the disease severity, including improvements in overall body growth, gross motor function and increase in the lifespan (>2-fold). Importantly, a marked increase in minimum (initial) survival (5-fold) of severe SMA mice by all three JNK inhibitors is remarkable and suggests that early postnatal intervention with JNK inhibition has high potential to stabilize and extend initial survival of severe SMA mice. This could provide a window for initiating combinatorial therapy with SMN-dependent methods especially for SMA patients that may not have immediate accesses to health care due to financial and geographical limitations.

The sex-based analysis of the effect of JNK inhibitors show notable similarities and differences in overall growth and survival of SMA mice. Treatment with pan-JNK inhibitor (SP600125) shows higher overall growth in male (∼71%) compared with female (∼59%) SMA mice. Comparison of the growth period (days) shows similar improvement in males (∼64%) and females (∼62%) mice. Treatment with AS601245 also shows higher overall growth in males (∼78%) compared with females (∼29%), which is similar to the effect of SP600125. Interestingly, the growth period of male mice (∼109%) is ∼2.8-fold higher than female mice (∼39%) treated with AS6001245, which is also higher than the increase (∼35%) in growth period of males treated with SP600125. Analysis of SR12519 with highest efficacy for JNK3 isoform shows that increase in overall growth of male (∼49%) and female (∼46%) SMA mice is similar. Comparison of growth periods shows better improvement in the growth period of SMA males (∼83%) than females (∼61%) treated with SR12519. The IC_50_ values for inhibition of JNK isoforms of three drug compounds are as follows: SP600125: JNK1 (40 nM), JNK2 (40 nM), and JNK3 (90 nM), AS601245: JNK1 (150 nM), JNK2 (220 nM), and JNK3 (70 nM) and SR12519: JNK1 (21 nM), JNK2 (66 nM), and JNK3 (15 nM).^56-59^ These IC_50_ values suggest that all drug compounds may fall in the category of pan-JNK inhibitors with diverse efficacy of inhibition for JNK isoforms. Interestingly, our *in vivo* data show that SR12519 with highest potency for JNK3 was able to effectively inhibit JNK activation in the spinal cords but not in skeletal muscle compared with SP600125 and AS601245. These data suggest that SR12519 may be a JNK3-specific inhibitor because skeletal muscle do not express JNK3 but express JNK1 and JNK2 and treatment with SR12519 was not effective in inhibiting JNK in skeletal muscle.^83-87^ Comparison of potency (IC_50_) of three JNK inhibitors, alongside sex-based analyses of overall growth in SMA mice, provided insight into the relationship between inhibitor efficacy across JNK isoforms and sex-specific improvements in disease phenotype. Comparison of sex-specific growth outcomes between SP600125 and AS601245 treatments shows a male-to-female improvement ratio of 1.2 with SP600125 and 2.69 with AS601245. This suggests that the reduced efficacy for JNK1/2 and enhanced selectivity for JNK3 in AS601245 confers a 2.24-fold greater growth improvement in males than females with severe SMA. Furthermore, analysis of the male-to-female growth period ratio reveals greater improvement in males treated with AS601245 (2.8) compared with SP600125 (1.03), suggesting that the isoform-selective JNK inhibition profile of AS601245 may offer enhanced therapeutic benefit for males. Interestingly, comparison of data from SMA mice treated with SR12519, which has highest efficacy for JNK3 and the second best for JNK1 among the three tested compounds, shows that the ratio of male-to-female growth is 1.06, indicating similar growth improvement among male and female SMA mice. These data suggest increased efficacy of JNK3 inhibition leads to greater improvement in females, resulting in a comparable reduction in disease severity between male and female SMA mice. This observation aligns with previous findings from genetic ablation of the *Jnk3* gene in SMAΔ7 mice.^26^ Sex-specific analysis of lifespan extension following treatment with JNK inhibitors revealed the following fold increases: SP600125, 2.13-fold (male) and 2.02-fold (female); AS601245, 2.36-fold (male) and 2.30-fold (female); and SR12519, 1.8-fold (male) and 2.06-fold (female). These results demonstrate an average increase in lifespan of 2-fold or more, consistent with the finding that genetic ablation of *Jnk3* similarly extended the lifespan of severe SMA mice 2-fold.^26^ A particularly notable finding is the 5-fold increase in the minimum (initial) survival of both male and female SMA mice treated with each of the three JNK inhibitors, compared with vehicle treated controls.

Tremendous research progress and efforts have been made to utilize the potential of the JNK signalling pathway in the treatment of neurodegenerative disorders, including Alzheimer’s disease, ALS, Huntington’s disease, and Parkinson’s disease.^32-43,96-99^ However, the complexity associated with the JNK group of kinases, including the 3 genes, *Jnk1*, *Jnk2*, and *Jnk3* that generate 10 isoforms due to alternative splicing and functional redundancy among the kinases with wide expression of *Jnk1* and *Jnk2* in adult tissues and *Jnk3* mainly expressed in the CNS^100-102^ has contributed to the limited success; however, the academia and pharmaceutical industry maintain high-interest in exploiting JNK kinases as therapeutic targets.^35,98,103^ To gain insight into the impact of inhibition of different JNK isoforms on the rescue of SMA phenotype, we analysed mice survival data in the context of efficacy (IC_50_) drug compounds towards different JNK isoforms. Inhibition with AS601245 shows highest average increase in the lifespan of severe SMA mice, 2.36-fold (male) and 2.30-fold (females), which may be due to a combination of ∼2.14-fold higher efficacy of JNK3 (70 nM) compared with JNK1 (150 nM). Inhibition with another pan-JNK inhibitor SP600125 with ∼2.25-fold higher efficacy for JNK1 (40 nM) compared with JNK3 (90 nM) shows similar increase in the lifespan of male (2.13-fold) and female (2.02-fold) SMA mice, which is comparable to ∼2.30-fold increase in the lifespan of SMA mice treated with AS601245. Furthermore, analysis of data from the treatment with SR12519, the inhibitor with the highest efficacy for JNK3 (15 nM) and JNK1 (21 nM) among the three drug compounds, shows slightly lower increase in the survival of males (1.8-fold) and similar increase in females (2.06-fold) compared with SP600125 and SR12519. These data suggest that drug compounds with very high potency such as SR12519 may not yield best results at the tested concentration and may require optimization. The notable outcomes of the analysis of severe SMA mice treated with AS601245, including significant increase in the survival and robust improvement in the gross motor function with the ability to walk up to 18 days of age, suggest that drug compounds with double inhibition efficacy for JNK3 compared with JNK1 maybe suitable for treatment because JNK3-deficiency can be complemented by JNK1 and partial inhibition of JNK1 will allow maintenance of critical functions of JNK1 in neuron growth and survival.^104-106^

### SMN-independent amelioration of SMA disease by pharmacological JNK inhibition

One of the goals of this study was to test and establish whether pharmacological inhibition of JNK is also SMN-independent. Analysis of SMN levels in the spinal cord and skeletal muscle tissues of SMA mice treated with JNK inhibitors did not show any statistically significant change in the SMN levels compared with control SMA mice treated with DMSO suggesting that the drug compounds did not alter SMN levels. However, the compounds demonstrated robust biological activity towards inhibiting JNK by preventing p-c-Jun and maintaining its levels close to p-c-Jun levels in normal mice. The drug compounds did not affect the levels of total c-Jun supporting the specificity of compounds for JNK inhibition. The specificity of the inhibitor SR12519 with highest potency for JNK3 was demonstrated in skeletal muscle tissue where the p-c-Jun levels were decreased by only ∼12% compared with control SMA mice treated with DMSO. JNK activation was shown in skeletal muscle cells and cultured primary muscle derived from SMA patients.^107,108^ However, what isoform of JNK is primarily activated in muscle is unclear. It is possible that both JNK1 and JNK2 are activated in muscle because JNK3 is mainly expressed in the CNS.^100-102^ The current data suggest that JNK2 may be mainly activated by SMN-deficiency in the skeletal muscle of SMA mice or an alternatively spliced form of either JNK1 or JNK2 that is not efficiently inhibited by SR12519. Interestingly, comparison of survival and motor function data from three inhibitors and levels of p-c-Jun in spinal cord and skeletal muscle shows that both SP600125 and AS601245 were able to efficiently inhibit JNK in the spinal cords and skeletal muscle in contrast to SR12519. The lower average survival of SMA mice treated with SR12519 ∼1.78-fold (males) and ∼2.0-fold (females) compared with average survival of either male or female mice (∼2.3-fold) treated with SP600125 and AS601245 is likely because of partial inhibition of JNK in skeletal muscle by SR12519 suggesting that JNK inhibition in skeletal muscle may also be important and contribute to muscle strength and improve motor function along with extending the lifespan of SMA mice. Together, these data suggest that pan-JNK inhibitors may be suitable for improvement in overall growth, motor function and lifespan. However, JNK1/JNK3-specific inhibitors may be useful in preventing neurodegeneration and JNK1/JNK2-specific drugs may be suitable for improving muscle strength and in ameliorating muscle atrophy. The present study demonstrates a proof-of-concept that pharmacologic inhibition of JNK is a viable option for the treatment of SMA. Further studies are required to gain insight into *in vivo* the molecular mechanisms and contributions of preservation of motor neurons and NMJs in disease rescue. The ongoing efforts towards the design and testing of new generation of JNK isoform specific inhibitors will allow better selection of JNK inhibitors for the treatment of JNK3-mediated pathogenesis associated with SMA and other neurodegenerative disorders, including ALS, Alzheimer’s disease, Huntington’s disease, and Parkinson’s disease.,^41,46,109-112^

Comparison of SMA rescue phenotype by genetic ablation of *Jnk3* and other SMN-independent modifiers, *PLS3* and *Z*  *+*  *Agrin* using the SMAΔ7 mouse model shows that deletion of *Jnk3* gene did not change SMN levels and resulted in systemic rescue, including increase in motor neuron survival, muscle fibre diameter and a 2-fold increase in the lifespan of SMA mice.^26^ In contrast, overexpression of *PLS3* and *Z*  *+*  *Agrin* in SMAΔ7 caused slight improvement in disease phenotype but did not extend the lifespan of SMA mice suggesting that JNK3-mediated rescue shows robust improvements and 2-fold increase in the survival compared with PLS3 and Z + Agrin-mediated rescue in SMAΔ7 mice.^24,113,114^ Furthermore, comparison of genetic and pharmacological inhibition of JNK reveals similar systemic rescue with >2-fold increase in the lifespan of SMAΔ7 mice by pharmacological inhibition, demonstrating the reproducibility of beneficial effects of JNK inhibition on the amelioration of the SMA phenotype with two independent approaches.^26^ The idea of using JNK inhibition in combination with SMN-dependent methods may also show beneficial effects as supported by studies of *NCALD* reduction (SMN-independent) and treatment with Nusinersen (SMN-dependent) using ASOs that ameliorates SMA in mice;^22^ moreover, a combination of myostation activity inhibition and increase in SMN levels with splice modulator SMN-C1 also ameliorates SMA in mice.^115^

An outstanding issue concerning the partial rescue with SMN-independent methods is related to chronic low levels of ubiquitously expressed SMN protein leading to a multisystem disease. Therefore, it may not be possible to achieve full rescue and cure SMA using only SMN-independent strategies and a combination of both SMN-dependent and SMN-independent methods may be most beneficial.^116,117^ The reasons for a partial rescue are unclear and may require further investigations. However, the possible reasons for the observed rescue alongside significant extension in the survival of SMA mice treated with JNK inhibitors may include: (i) functional redundancy of JNK isoforms. Genetic studies in mice have shown that JNK1 and JNK2 can complement each other and compensate for JNK3 deficiency,^101^ (ii) experimental evidences indicate that SMN may have distinct biological functions that may require elevation of SMN levels,^118^ (iii) SMN-deficiency may not result in the activation of the JNK pathway in all cell types, including fibroblasts,^119^ (iv) may cause activation of other MAPK signalling pathways in different cell types,^74,120,121^ (v) may contribute to either apoptosis or autophagy that cause neurodegeneration,^122,123^ (iv) contribution of JNK activation in other tissues and organs such as liver,^124,125^ heart,^126-128^ lung,^129^ pancreas,^130^ and vasculature,^131-133^ gastric^134^ and respiratory (diaphragm and phrenic nerve) systems^51^ that are affected in SMA, which may contribute to the systemic pathogenesis but warrants further studies. Nevertheless, highly reproducible data on improvements in the overall growth, motor functions, including righting reflexes, ability to walk in the late stages (age:15–18 days) of life, a 2-fold increase in median survival and a remarkable 5-fold increase in the initial survival of male and female SMA mice with three tested JNK inhibitors presenting distinct chemical composition and 3D-conformation support the idea of investing and investigating the beneficial effects of JNK inhibitors in future clinical trials with SMA patients.^135^ In addition, the data from this study and data from prior use of JNK inhibitors in the past and current clinical trials for the treatment of other human diseases will allow the development of suitable standalone and combinatorial strategies for clinical trials in SMA patients.^112,136-139^

## Conclusion

*In vitro* and *in vivo* testing of three JNK inhibitors with distinct chemical scaffolds and diverse efficacy and potency for different isoforms of JNK generated robust data that show similar amelioration of disease severity and the partial rescue of phenotype in mice with severe form of SMA. Overall, the findings of this study demonstrate the rigour of experimentation and analysis, which establish a proof-of-concept that the pharmacological inhibition of JNK is a viable SMN-independent method for neuroprotection and the treatment of SMA. These findings should allow the development of suitable standalone and combination therapeutic strategies involving SMN level enhancement for the treatment of mild to severe forms of SMA.

## Supplementary Material

fcag111_Supplementary_Data

## Data Availability

Most of the data are presented in the figures, text and Supplementary material of the manuscript. However, any other data referenced is available upon reasonable request.
