# Supplementary material for: Survival motor neuron protein-independent amelioration of spinal muscular atrophy by pharmacological inhibition of c-Jun-NH2 terminal kinase
Source: Brain Commun. 2026 Mar 26;8(2):fcag111. doi: 10.1093/braincomms/fcag111 (PMC13058730; doi:10.1093/braincomms/fcag111)
Supplement: fcag111_Supplementary_Data [file fcag111_supplementary_data.zip › Supplementary_material.pdf]

SUPPLEMENTARY MATERIAL

SMN-independent amelioration of spinal muscular atrophy by pharmacological inhibition of JNK

Annapoorna Kannan[1†], Kanchan Bhatia[2†], Xiaoting Jiang[3†], Olivia Lesnik[4, 5], Sabahat B. Asad[4, 5], Kailyn Fiocca[6], Saif Ahmad[7], Yangbo Feng[8], Dana Branzei[9,10] and Laxman Gangwani[4, 5*]

[†]Annapoorna Kannan, Kanchan Bhatia and Xiaoting Jiang contributed equally to this work.

Author affiliations:

[1]Center for Human Genetics, University of Oxford, Oxford OX3 7BN, United Kingdom.

[2]School of Mathematical and Natural sciences, ASU New College, Arizona State University - West campus, Phoenix, AZ 85069, USA.

[3]Department of Immunology, Houston Methodist Research Institute, Houston, Texas 77030, USA.

[4]Bond Life Sciences Center and [5]Department of Pathobiology and Integrative Biomedical Sciences, University of Missouri, Columbia, MO 65211, USA.

[6]Hunt School of Dental Medicine, Texas Tech University Health Sciences Center El Paso, El Paso, TX 79905 USA.

[7]Department of Neurosurgery, Barrow Neurological Institute, St. Joseph's Hospital and Medical Center, Dignity Health, Phoenix, AZ 85013, USA.

[8]Department of Molecular and Cellular Pharmacology, Sylvester Comprehensive Cancer Center, Miller School of Medicine, University of Miami, Miami, FL 33136, USA.

[9]The AIRC Institute of Molecular Oncology Foundation, IFOM ETS, Via Adamello 16, Milan 20139, Italy.

[10]University of Duisburg-Essen, Research Center One Health Ruhr, University Alliance Ruhr Universität Straße 2, 45141, Essen Germany.

*Correspondence to: Laxman Gangwani, M.Sc., M.Tech., Ph.D.

Bond Life Sciences Center and Department of Pathobiology and Integrative Biomedical Sciences
University of Missouri,
Columbia, MO 65211, USA

Email: laxman.gangwani@missouri.edu

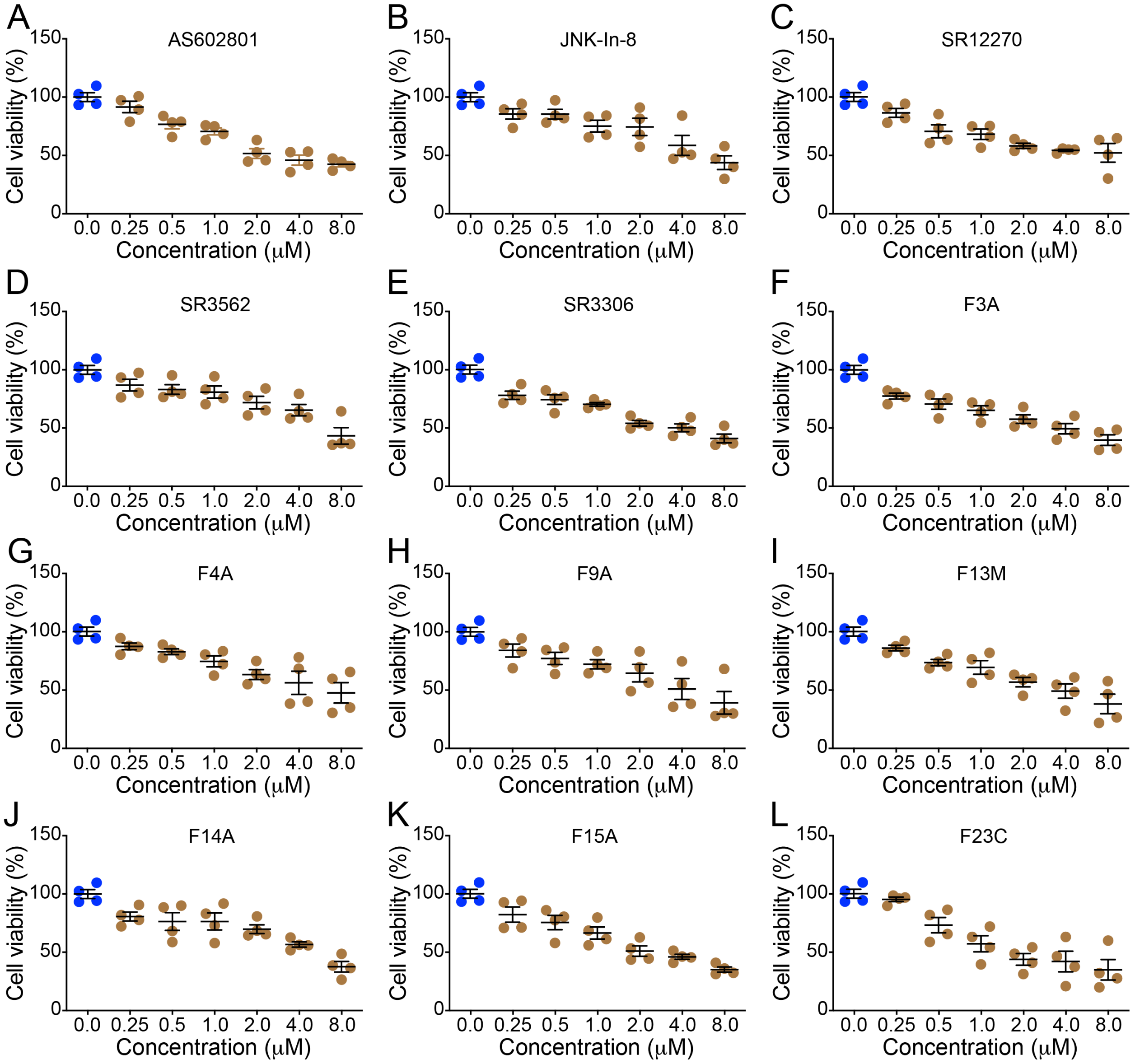

Supplementary Figure 1. The effect of JNK inhibitors on the cell viability of cultured primary neurons. Cerebellums were isolated from 7-day-old mice and cerebellar granule neurons (CGN) were purified and cultured *in vitro* for 8 days to from normal. Cell viability was determined using the MTT assay after treatment of neurons with drug compounds for 24 h. Twenty drug compounds were screened for cell viability and toxicity. The relative cell viability (%) is presented as scatter plots with individual points for different concentration of JNK inhibitors used for the treatment. Each data point represents an average of 3 technical replicates. The cell viability assay data for additional 12 compounds showing neuron survival (%) are presented as (mean ± s.e.m., n = 4) upon treatment with inhibitors at 1.0 mM, AS602801 (70.61 ± 2.98), JNK-In-8 (75.19 ± 4.94), SR12270 (68.14 ± 4.39), SR3562 (80.97 ± 5.17), SR3306 (70.27 ± 1.59), F3A (65.27 ± 3.88), F4A (74.48 ± 4.68), F9A (72.22 ± 4.02), F13M (69.31 ± 5.92), F14A (76.49 ± 7.28), F15A (66.39 ± 5.13) and F23C (57.19 ± 6.90). The cell viability data at other concentrations of drug compounds is presented in Supplementary Table 2.

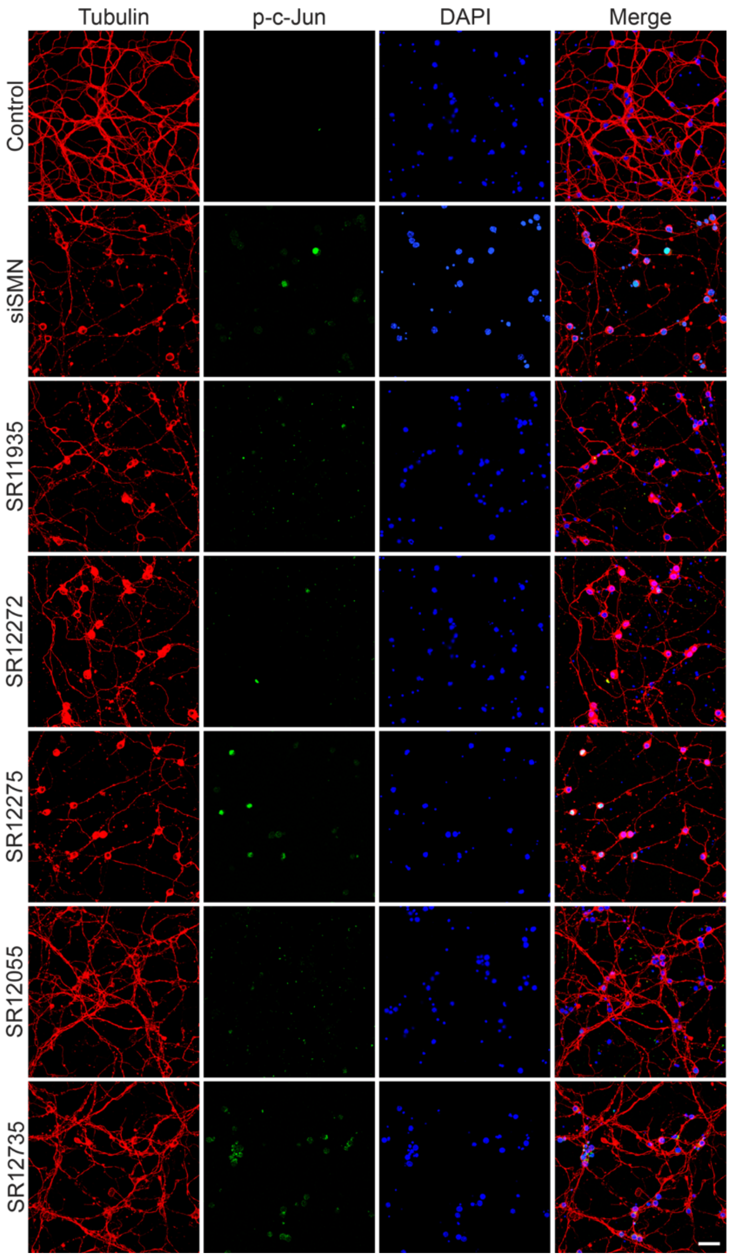

Supplementary Figure 2. JNK inhibition prevents degeneration of SMN-deficient cultured primary cerebellar granule neurons. Cultured primary neurons (CGN) were transfected with siRNA (siSMN) 100 nM and incubated for 24h before treatment with inhibitors (1.0 μM). Neurons were fixed after 24h treatment of inhibitor with 4% PFA and stained with antibodies for neuron-specific β-tubulin class III (red) and p-c-Jun (green) and IF examined by confocal laser scanning microscopy. The degeneration of neurons caused by SMN knockdown (siSMN) is shown by axonal degeneration compared to control neurons stained with tubulin (red). The nuclei are stained with DAPI (blue). Treatment with inhibitors, SR11935, SR12272, SR12275, SR12055, and SR12735 did not cause effective JNK inhibition and p-c-Jun (green) fluorescence was detectable. These JNK inhibitors did not prevent degeneration of neurons compared to SMN-deficient neurons (siSMN) and therefore were not selected for further testing. Scale bar is 50 μm.

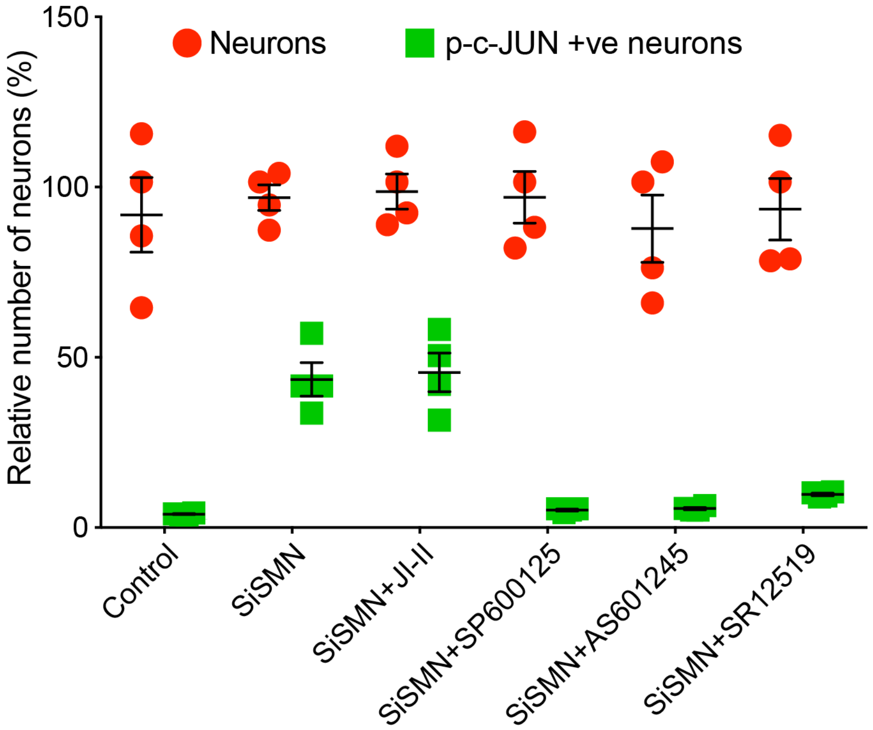

Supplementary Figure 3. Quantitation of neurons with JNK activation after SMN knockdown and treatment with JNK inhibitors. Cultured primary neurons (CGN) were transfected with siRNA (siSMN) 100 nM and incubated for 24h before treatment with inhibitors (1.0 µM). Neurons were fixed after 24h treatment of inhibitor with 4% PFA and stained with antibodies for neuron-specific β-tubulin class III (red) and p-c-Jun (green) and IF examined by confocal laser scanning microscopy. Total number of neurons were counted as Tubulin/DAPI positive cells (magenta) and neurons/nuclei with pc-Jun positive (green) in each group. The pc-Jun positive (green) cells were plotted against total number of neurons in each group as percent. Total of twenty images were counted, five 5 images from four experiments (n = 4) and the number of pc-Jun positive cells (mean ± s.e.m.)% are Control (2.41 ± 0.11) %, SiSMN (41.97 ± 4.90)%, JI-II (negative control) (44.05 ± 5.70)%, SP600125 (3.59 ± 0.25)%, AS601245 (4.05 ± 0.31)%, SR12519 (8.20 ± 0.36)%. The statistical significance measured using unpaired *t*-test with Welch's correction by comparing two groups Control vs SiSMN ($P = 0.0039$), SiSMN vs JI-II ($P = 0.7915$), SiSMN vs SP600125 ($P = 0.0042$), SiSMN vs AS601245 ($P = 0.0043$) and SiSMN vs SR12519 ($P = 0.0061$) show statistically significant reduction in the number of p-c-Jun positive (pc-Jun +ve) SMN-deficient neurons upon treatment with JNK inhibitors. Additional ANOVA analysis using three groups, Control, SiSMN, SP600125 ($P = 5.12 \times 10^{-6}$), Control, SiSMN, AS601245 ($P = 5.40 \times 10^{-6}$) and Control, SiSMN, SR12519 ($P = 8.02 \times 10^{-6}$) also confirms statistically significant reduction in the number of p-c-Jun positive SMN-deficient neurons upon treatment with three JNK inhibitors.

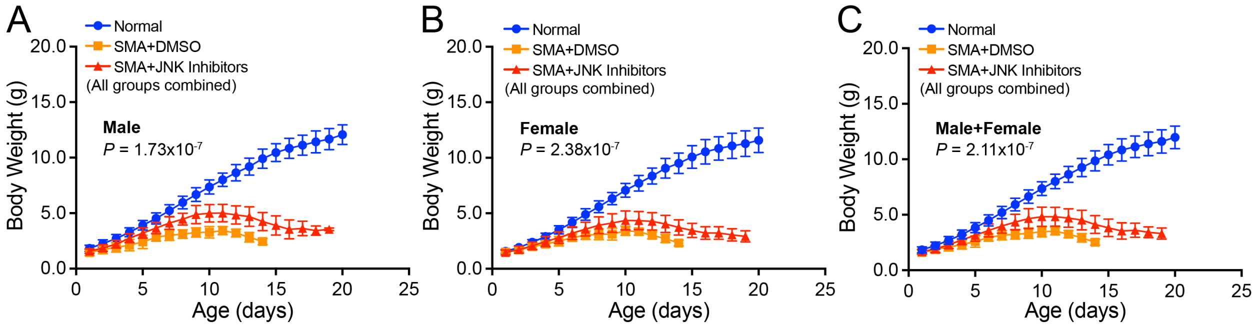

JNK Inhibitor	SP600125	AS601245	SR12519	Three inhibitors combined
Growth Analysis (overall)	Statistical Significance – (ANOVA) *P* value	Statistical Significance – (ANOVA) *P* value	Statistical Significance – (ANOVA) *P* value	Statistical Significance – (ANOVA) *P* value
Male	1.8×10^{-9}	7.08×10^{-7}	2.7×10^{-6}	1.73×10^{-7}
Female	5.5×10^{-8}	1.47×10^{-7}	2.9×10^{-6}	2.38×10^{-7}
Male+Female	3.2×10^{-9}	1.73×10^{-8}	6.5×10^{-7}	2.11×10^{-7}

Supplementary Figure 4. Pharmacological inhibition of JNK improves the overall growth and survival of SMA mice. SMA (SMAΔ7) mice littermates at postnatal day 2 (PND2) were injected intraperitoneally with a single dose of vehicle DMSO or either JNK inhibitor SP600125 (20 mg/kg) or AS601245 (20 mg/kg) or SR12519 (10 mg/kg). The data from three experiments with individual JNK inhibitors were combined to gain insight into the effect of JNK inhibition on overall growth of mice. Mice body weights recorded every day are presented as growth curves with mean ± SD, n = minimum 3 mice/group/inhibitor (n = 9) for male, female and combined (n = 18) (male+female) groups. Overall growth (body weight in g) curves of Normal (blue), SMA+DMSO (orange) and combined [(SMA+SP600125) + (SMA+AS601245) + (SMA+SR12519)] (red) mice littermates. Mice body weights recorded every day are presented as growth curves with mean ± SD. Statistical analysis was performed using one-way ANOVA and statistical significance (*P* values) of individual experiments with each inhibitor and three inhibitors combined are compared in the table. Both male and female SMA mice show significant improvement in the overall growth upon treatment with JNK inhibitors.

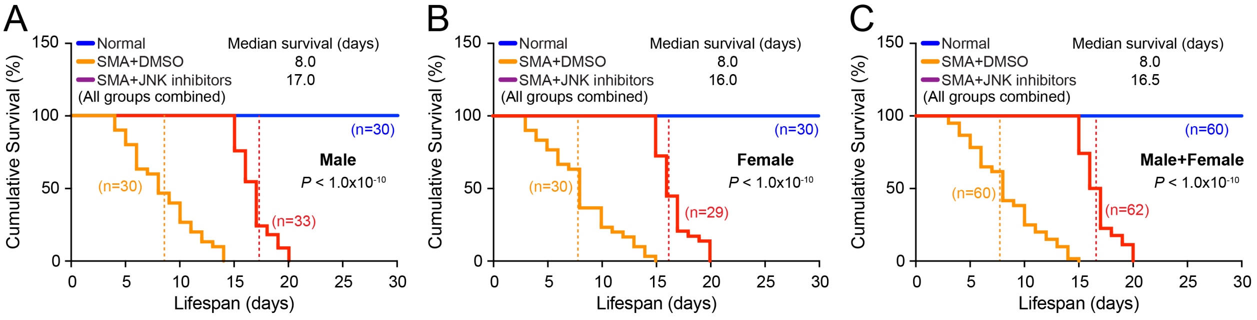

JNK Inhibitor	SP600125	AS601245	SR12519	Three inhibitors combined
Survival Analysis (Kaplan-Meier)	Statistical Significance – Log-rank (Mantel-Cox) test *P* value	Statistical Significance – Log-rank (Mantel-Cox) test *P* value	Statistical Significance – Log-rank (Mantel-Cox) test *P* value	Statistical Significance – Log-rank (Mantel-Cox) test *P* value
Male	$<1.0x10^{-10}$	$<1.0x10^{-10}$	$<1.0x10^{-10}$	$<1.0x10^{-10}$
Female	$<1.0x10^{-10}$	$<1.0x10^{-10}$	$<1.0x10^{-10}$	$<1.0x10^{-10}$
Male+Female	$<1.0x10^{-10}$	$<1.0x10^{-10}$	$<1.0x10^{-10}$	$<1.0x10^{-10}$

Supplementary Figure 5. Pharmacological inhibition of JNK improves the survival of SMA mice. SMA (SMAΔ7) mice littermates at postnatal day 2 (PND2) were injected intraperitoneally with a single dose of vehicle DMSO or either JNK inhibitor SP600125 (20 mg/kg) or AS601245 (20 mg/kg) or SR12519 (10 mg/kg). The data from three experiments with individual JNK inhibitors were combined to gain insight into the effect of JNK inhibition on the survival of mice. The Kaplan-Meier survival curves of Normal (blue), SMA+DMSO (orange) and combined [(SMA+SP600125)+(SMA+AS601245)+(SMA+SR12519)] (red) mice littermates. Dotted lines show median survival. Statistical analysis was performed using log-rank (Mantel-Cox) test and statistical significance (*P* values) of individual experiments with each inhibitor and three inhibitors combined are compared in the table. Both male and female SMA mice show significant increase in the survival upon treatment with JNK inhibitors.

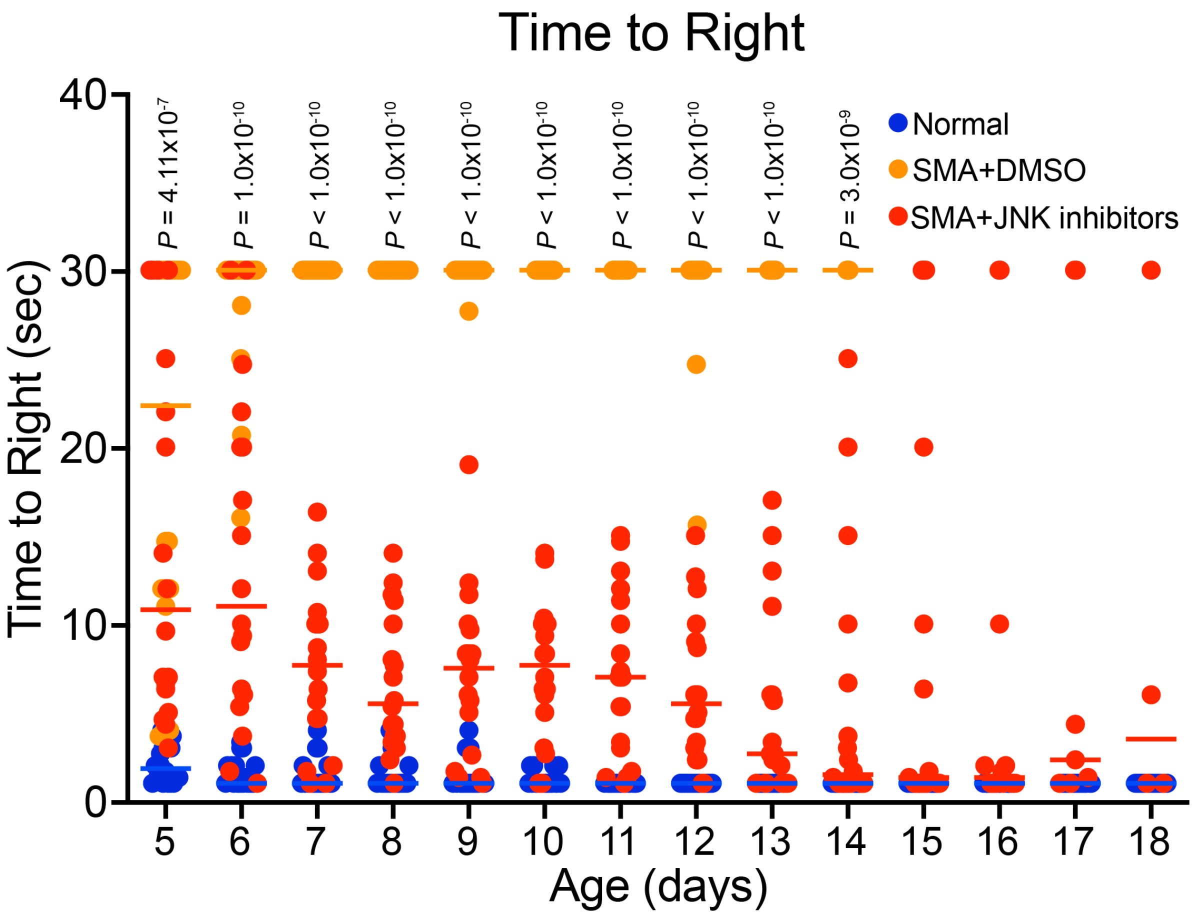

Supplementary Figure 6. Pharmacological JNK inhibition improves the gross motor function of SMA mice. SMA (SMAΔ7) mice littermates at postnatal day 2 (PND2) were injected intraperitoneally with a single dose of vehicle DMSO or either JNK inhibitor SP600125 (20 mg/kg) or AS601245 (20 mg/kg) or SR12519 (10 mg/kg). The data from three experiments with individual JNK inhibitors were combined to gain insight into the effect of JNK inhibition on the improvement of motor function. Mice righting reflexes were examined for 5 to18-day-old Normal (blue), SMA+DMSO (orange), and combined [(SMA+SP600125) + (SMA+AS601245) + (SMA+SR12519)] (red) mice littermates. Time-to-right (TTR) with a time limit of 30 seconds for testing and an average of 3 recordings per pup/day/group are plotted. Data were collected from three groups starting with n = 18 mice/group at PND5 [18 normal (blue), 18 SMA+DMSO (orange), 18 SMA+JNK inhibitors (red). Individual data points (mean ± s.e.m.) are average of three recordings/day/pup and presented as a scatter plot. Improvement in the motor function (righting) is demonstrated by an age-dependent increase in the ability of mice to right faster upon treatment with JNK inhibitors compared to control SMA mice treated with DMSO (SMA+DMSO). SMA mice were unable to right themselves by at the age of 12 days. Notably, SMA mice treated with JNK inhibitors were able to right themselves up to the age of 18 days. Statistical analysis was performed using ANOVA and statistical significance (*P* values) show JNK inhibition resulted in highly significant improvement in the motor function of SMA mice from the age of 5 to 14 days. SMA mice treated with JNK inhibitors were able to right themselves until 18 days of age.

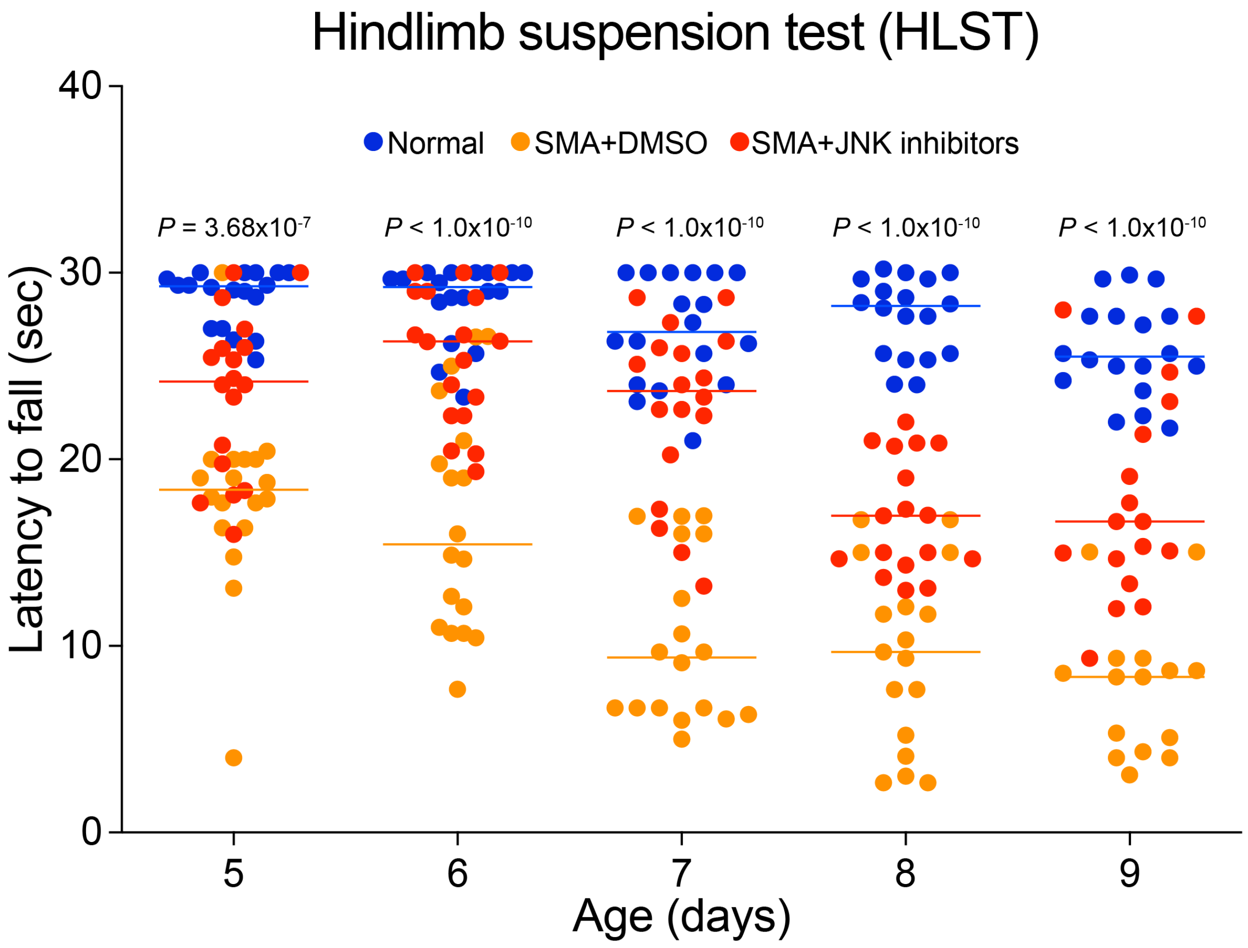

Supplementary Figure 7. Pharmacological JNK inhibition improves the muscle strength of SMA mice. SMA (SMAΔ7) mice littermates at postnatal day 2 (PND2) were injected intraperitoneally with a single dose of vehicle DMSO or either JNK inhibitor SP600125 (20 mg/kg) or AS601245 (20 mg/kg) or SR12519 (10 mg/kg). The data from three experiments with individual JNK inhibitors were combined to gain insight into the effect of JNK inhibition on increase in muscle strength. Mice Normal (blue), SMA+DMSO (orange), and combined [(SMA+SP600125) + (SMA+AS601245) +(SMA+SR12519)] (red) littermates were examined for muscle using the hind-limb suspension test (HLST). Littermates of 5 to 9-days of age were hung on both hind legs on the edge of a 50 ml plastic conical tube and time (sec) was recorded until fall from the edge of the tube with a time limit of 30 seconds for testing and an average of 3 recordings per pup/day/group (individual points) are plotted. Data were collected from three groups starting with n = 18 mice/group at PND5 [18 normal (blue), 18 SMA+DMSO (orange), 18 SMA+JNK inhibitors (red). Latency to fall (mean ± s.e.m.) is shown as a scatter plot. Improvement in the hindlimb muscle strength is demonstrated by an increase in the ability of mice to hang on their hindlimbs upon treatment with JNK inhibitors compared to control SMA mice treated with DMSO (SMA+DMSO). Statistical analysis (ANOVA) shows a marked and statistically significant increase in the hanging time for JNK inhibitor treated SMA mice compared to DMSO treated SMA mice and control; PND5 [SMA+JNK inhibitors (23.65±0.59) and SMA+DMSO (28.65±0.35), $P = 3.68 \times 10^{-7}$], PND6 [SMA+JNK inhibitors (25.56±0.84) and SMA+DMSO (16.74±1.42), P <

1.0×10^{-10}], PND7 [SMA+JNK inhibitors (22.73±1.08) and SMA+DMSO (10.26±1.05), $P < 1.0 \times 10^{-10}$], PND8 [SMA+JNK inhibitors (17.01±0.76) and SMA+DMSO (9.49±1.17), $P < 1.0 \times 10^{-10}$] and PND9 [SMA+JNK inhibitors (17.74±1.33) and SMA+DMSO (7.80±0.94), $P < 1.0 \times 10^{-10}$] shows a gradual increase in the muscle strength of JNK inhibitor treated mice compared to control SMA mice.

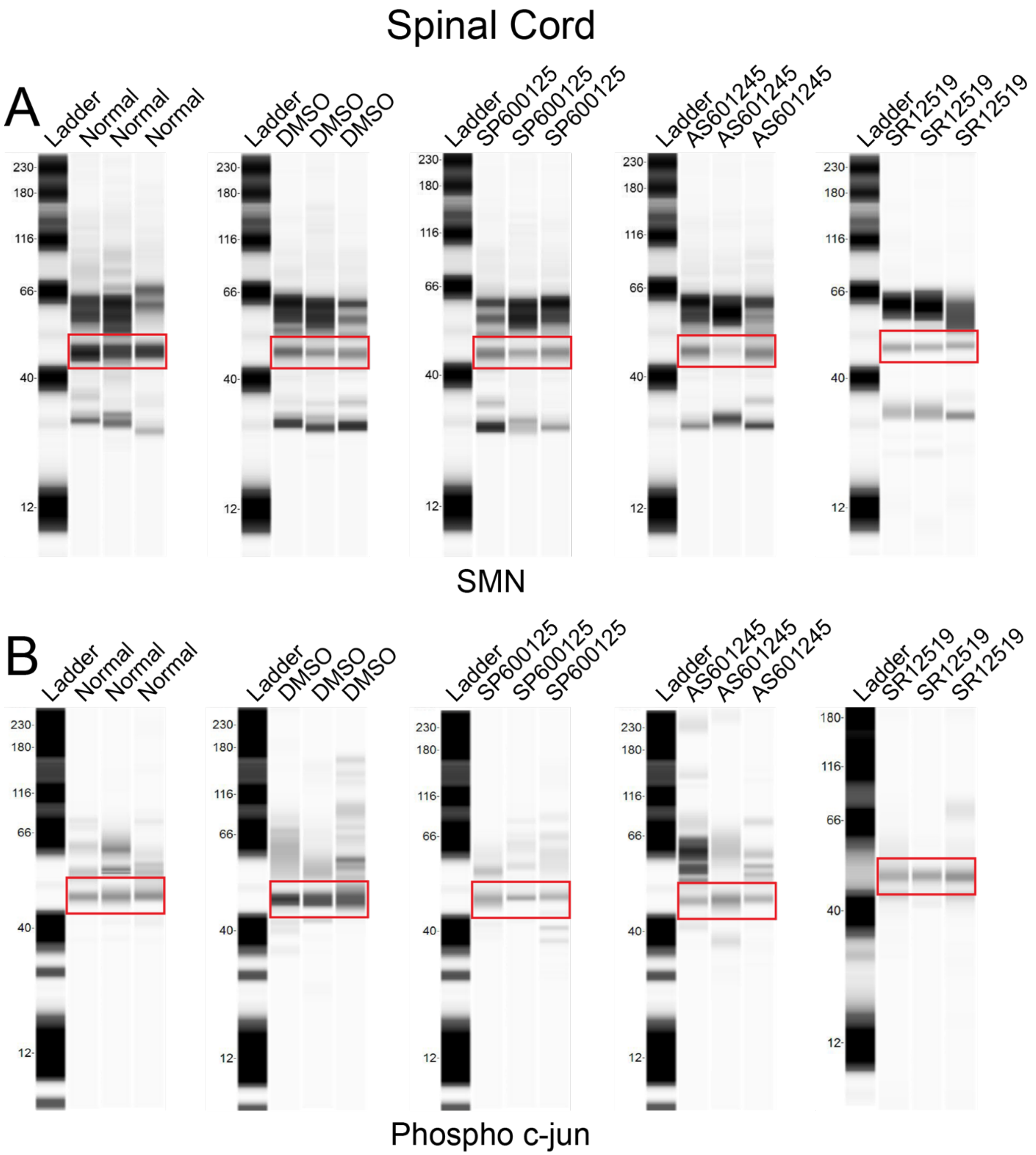

Supplementary Figure 8. The uncropped (full-length) blots of cropped images presented in manuscript Figure 9A. Gel bands cropped are highlighted with red color box.

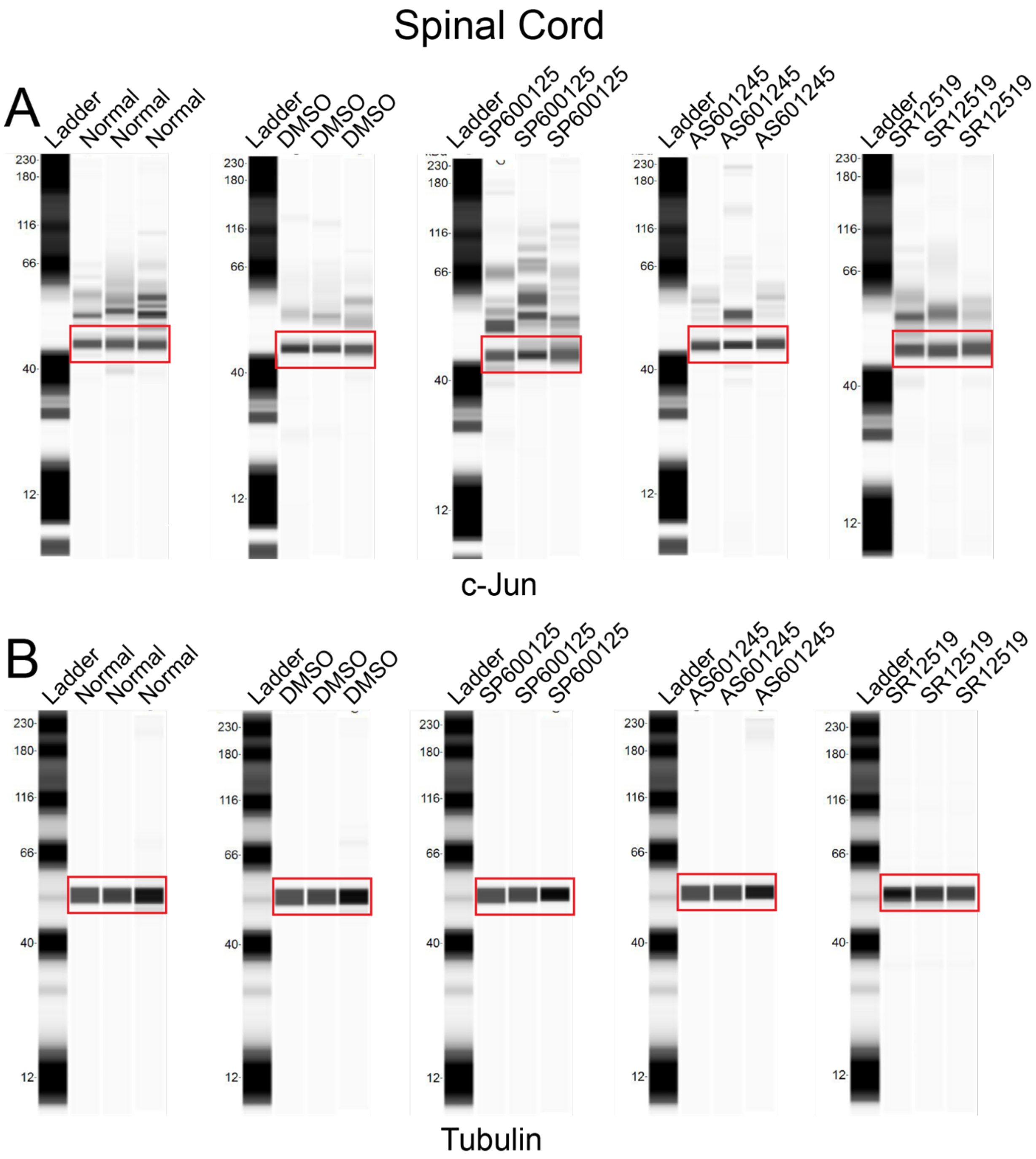

Supplementary Figure 9. The uncropped (full-length) blots of cropped images presented in manuscript Figure 9A. Gel bands cropped are highlighted with red color box.

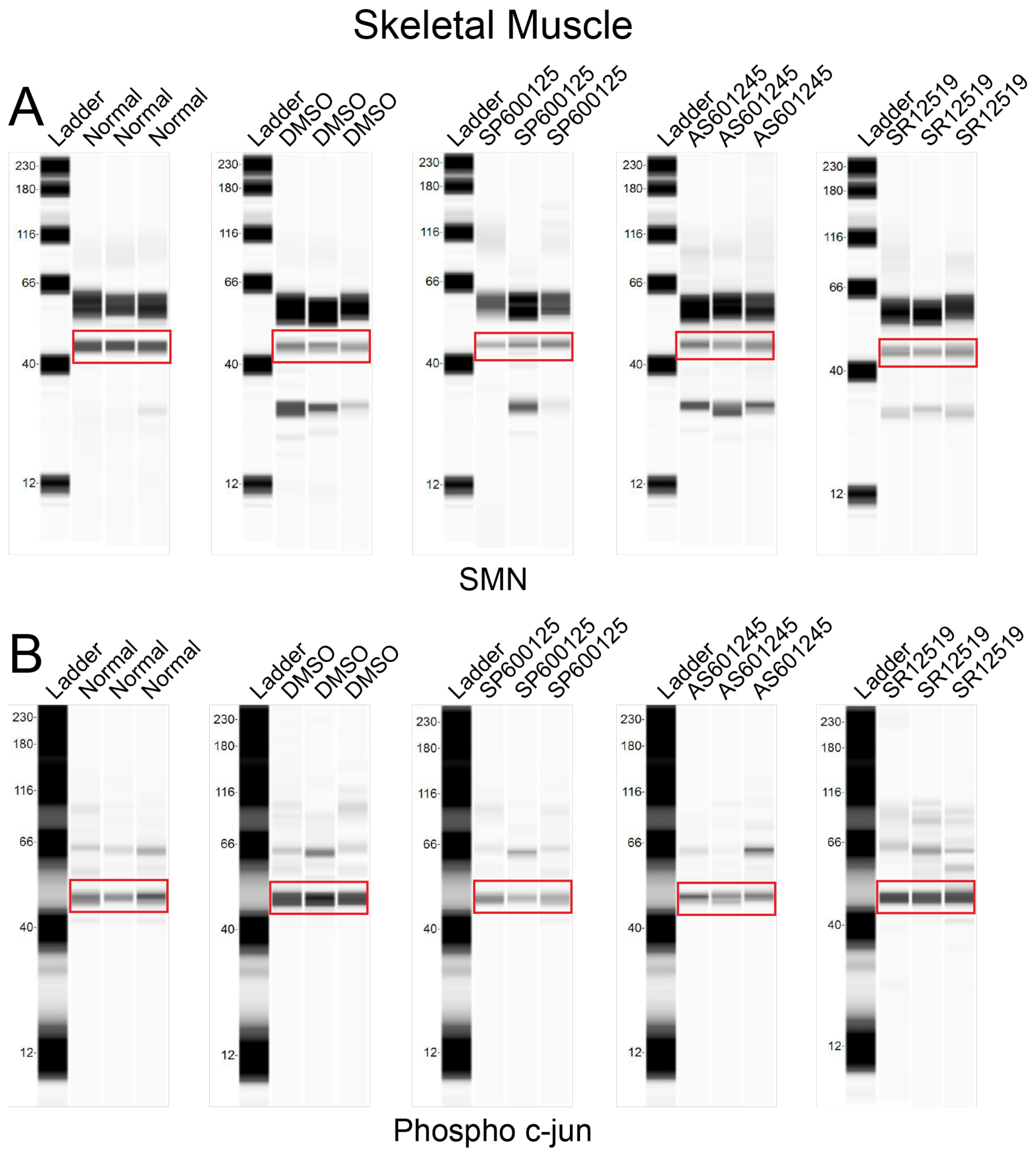

Supplementary Figure 10. The uncropped (full-length) blots of cropped images presented in manuscript Figure 9D. Gel bands cropped are highlighted with red color box.

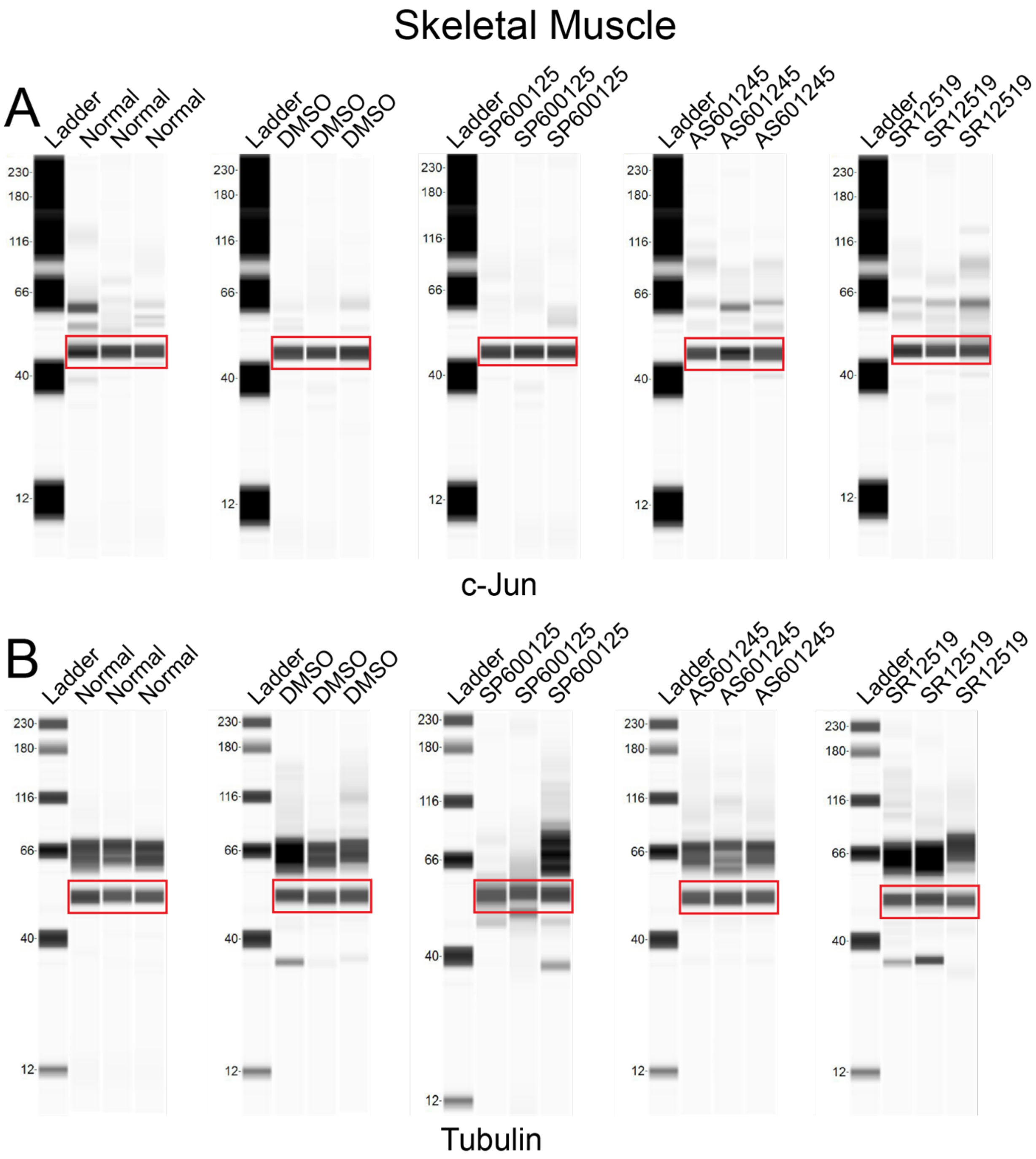

Supplementary Figure 11. The uncropped (full-length) blots of cropped images presented in manuscript Figure 9D. Gel bands cropped are highlighted with red color box.

Supplementary Table 1. The effect of JNK inhibitors on the cell viability of cultured primary neurons.

**Compound**	Cell viability (%)						
**Conc. (uM)**	**0.0**	**0.25**	**0.50**	**1.0**	**2.0**	**4.0**	**8.0**
**SP600125**	100.0 ± 3.85	103.60± 7.69	95.33 ± 3.23	91.32 ±7.48	87.21 ± 5.28	66.47 ± 4.40	40.53 ±9.49
**AS601245**	100.0 ± 3.85	97.01 ± 2.72	90.98 ± 1.07	89.14 ± 3.05	83.62 ± 6.98	71.98 ± 3.53	60.26 ± 2.67
**SR11935**	100.0 ± 3.85	92.38 ± 3.20	88.25 ± 5.76	82.37 ± 3.79	69.62 ± 4.23	63.76 ± 3.84	61.38 ± 5.17
**SR12735**	100.0 ± 3.85	85.33 ± 1.53	84.11 ± 9.67	71.90 ± 4.30	60.51 ± 6.01	58.43 ± 2.91	49.52 ± 5.91
**SR12272**	100.0 ± 3.85	94.06 ± 5.22	88.84 ± 6.37	82.10 ± 5.73	79.74 ± 2.41	64.97 ± 5.79	50.06 ± 7.77
**SR12275**	100.0 ± 3.85	85.13 ± 6.27	80.75 ± 1.65	74.75 ± 5.68	61.99 ± 2.87	52.26 ± 1.60	52.07 ± 8.63
**SR12519**	100.0 ± 3.85	94.52 ± 8.39	95.73 ± 8.75	80.02 ± 6.17	60.81 ± 2.60	49.44 ± 8.01	48.90 ± 6.35
**SR12055**	100.0 ± 3.85	91.53 ± 3.11	87.39 ± 2.05	81.41 ± 2.62	74.50 ± 3.95	61.87 ± 2.43	47.57 ± 4.77

These data (mean ± s.e.m.) are presented as scatter plots in Figure 1(A-H).

Supplementary Table 2. The effect of JNK inhibitors on the cell viability of cultured primary neurons.

**Compound**	Cell Viability (%)						
**Conc. (uM)**	**0.0**	**0.25**	**0.50**	**1.0**	**2.0**	**4.0**	**8.0**
**AS602801**	100.0 ± 3.85	91.56 ± 4.84	76.69 ± 3.83	70.61 ± 2.98	51.65 ± 4.20	45.99 ± 4.31	42.49 ± 2.28
**JNK-In-8**	100.0 ± 3.85	85.59 ± 4.42	85.42 ± 4.17	75.19 ± 4.94	74.50 ± 7.40	58.64 ± 8.60	43.85 ± 5.89
**SR12270**	100.0 ± 3.85	86.39 ± 3.85	70.61 ± 5.58	68.14 ± 4.39	58.21 ± 2.22	54.30 ± 0.94	52.16 ± 7.97
**SR3562**	100.0 ± 3.85	86.93± 5.04	83.16 ± 4.14	80.97 ± 5.17	71.97 ± 5.36	65.40 ± 4.87	43.41 ± 7.03
**SR3306**	100.0 ± 3.85	77.97 ± 3.56	74.30 ± 4.12	70.27 ± 1.59	54.04 ± 2.33	50.21 ± 3.38	41.08 ± 3.71
**F3A**	100.0 ± 3.85	77.51 ± 2.57	70.66 ± 4.46	65.27 ± 3.88	57.74 ± 3.68	49.48 ± 4.44	39.77 ± 4.56
**F4A**	100.0 ± 3.85	87.34 ± 2.90	82.75 ± 2.46	74.48 ± 4.68	63.24 ± 4.25	56.13 ± 9.98	47.58 ± 8.73
**F9A**	100.0 ± 3.85	84.02 ± 5.57	77.18 ± 5.24	72.22 ± 4.02	64.57 ± 7.53	50.97 ± 9.00	39.12 ± 9.71
**F13M**	100.0 ± 3.85	85.94 ± 2.40	73.44 ± 2.70	69.31 ± 5.92	56.73 ± 4.01	49.03 ± 6.13	38.11 ± 8.39
**F14A**	100.0 ± 3.85	80.68 ± 3.88	76.43 ± 7.66	76.49 ± 7.28	69.86 ± 3.73	56.72 ± 2.25	37.63 ± 4.53
**F15A**	100.0 ± 3.85	82.20 ± 6.46	75.43 ± 6.09	66.39 ± 5.13	51.04 ± 4.44	46.03 ± 2.12	35.16 ± 2.17
**F23C**	100.0 ± 3.85	95.21 ± 1.93	73.13 ± 6.52	57.19 ± 6.90	43.80 ± 4.96	42.01 ± 8.79	34.91 ± 8.71

These data (mean ± s.e.m.) are presented as scatter plots in supplementary Figure 1.

Supplementary Table 3. The comparison of beneficial effects of *in vivo* treatment with three JNK inhibitors on the amelioration of disease severity in SMA mice.

Treatment/ Improvement	DMSO	SP600125	DMSO	AS601245	DMSO	SR12519
**Survival (Male) (days)**		Days (fold change)*		Days (fold change)*		Days (fold change)*
Min	3	15 (5.0)	3	15 (5.0)	3	15 (5.0)
Max	14	19	11	20	14	17
Median	7.5	17 (2.27)	8	17 (2.12)	9	16 (1.78)
**Survival (Female) (days)**						
Min	3	15 (5.0)	3	15 (5.0)	3	15 (5.0)
Max	15	20	14	20	14	17
Median	9	17 (1.88)	8	16.5 (2.06)	7.5	15.5 (2.06)
**Survival (Male+Female) (days)**						
Min	3	15 (5.0)	3	15 (5.0)	3	15 (5.0)
Max	15	20	14	20	14	17
Median	8.5	17 (2.0)	8	17 (2.12)	8	16 (2.0)
**Growth (g)**						
Male	3.26 ± 0.24	5.55 ± 0.38	3.05 ± 0.31	5.43 ± 0.39	3.54 ± 0.15	5.28 ± 0.32
Female	3.37 ± 0.28	4.87 ± 0.49	3.47 ± 0.14	4.48 ± 0.13	3.19 ± 0.18	4.67 ± 0.17
Male+Female	3.31 ± 0.17	5.21 ± 0.31	3.26 ± 0.17	4.96 ± 0.26	3.36 ± 0.12	4.98 ± 0.20
**Growth Period (days)**						
Male	6.25 ± 1.10	10.25 ± 0.25	5.75 ± 0.75	12.00 ± 0.40	6.0 ± 0.57	11.00 ± 0.57
Female	6.50 ± 1.19	10.50 ± 0.28	7.75 ± 0.48	10.75 ± 0.47	7.66 ± 0.88	12.33 ± 0.33
Male+Female	6.37 ± 0.75	10.38 ± 0.18	6.75 ± 0.55	11.13 ± 0.39	6.83 ± 0.60	11.67 ± 0.42

*Numbers in parenthesis show fold change in survival of SMA mice treated with JNK inhibitor compared to DMSO treated SMA mice.

Supplementary Videos

Supplementary Video 1 (6 days, SP600125)

Supplementary Video 2 (10 days, SP600125)

Supplementary Video 3 (15 days, SP600125)

Supplementary Video 4 (6 days, AS601245)

Supplementary Video 5 (14 days, AS601245)

Supplementary Video 6 (17 days, AS601245)

Supplementary Video 7 (8 days, SR12519)

Supplementary Video 8 (10 days, SR12519)

Supplementary Video 9 (13 days, SR12519)
